# Large-Scale Quality Analysis of Published ChIP-seq Data

**DOI:** 10.1534/g3.113.008680

**Published:** 2013-12-17

**Authors:** Georgi K. Marinov, Anshul Kundaje, Peter J. Park, Barbara J. Wold

**Affiliations:** * Division of Biology, California Institute of Technology, Pasadena, California 91125; † Center for Biomedical Informatics, Harvard Medical School, Boston, Massachusetts 02115; ‡ Informatics Program, Children’s Hospital Boston, Boston, Massachusetts 02115; § Division of Genetics, Brigham and Women’s Hospital, Boston, Massachusetts 02115; ** Computer Science and Artificial Intelligence Laboratory, Massachusetts Institute of Technology, Cambridge, Massachusetts 02139; †† The Broad Institute of Massachusetts Institute of Technology and Harvard, Cambridge, Massachusetts 02142

**Keywords:** ChIP-seq, chromatin immunoprecipitation, cross-correlation, quality assessment, transcription factor

## Abstract

ChIP-seq has become the primary method for identifying *in vivo* protein–DNA interactions on a genome-wide scale, with nearly 800 publications involving the technique appearing in PubMed as of December 2012. Individually and in aggregate, these data are an important and information-rich resource. However, uncertainties about data quality confound their use by the wider research community. Recently, the Encyclopedia of DNA Elements (ENCODE) project developed and applied metrics to objectively measure ChIP-seq data quality. The ENCODE quality analysis was useful for flagging datasets for closer inspection, eliminating or replacing poor data, and for driving changes in experimental pipelines. There had been no similarly systematic quality analysis of the large and disparate body of published ChIP-seq profiles. Here, we report a uniform analysis of vertebrate transcription factor ChIP-seq datasets in the Gene Expression Omnibus (GEO) repository as of April 1, 2012. The majority (55%) of datasets scored as being highly successful, but a substantial minority (20%) were of apparently poor quality, and another ∼25% were of intermediate quality. We discuss how different uses of ChIP-seq data are affected by specific aspects of data quality, and we highlight exceptional instances for which the metric values should not be taken at face value. Unexpectedly, we discovered that a significant subset of control datasets (*i.e.*, no immunoprecipitation and mock immunoprecipitation samples) display an enrichment structure similar to successful ChIP-seq data. This can, in turn, affect peak calling and data interpretation. Published datasets identified here as high-quality comprise a large group that users can draw on for large-scale integrated analysis. In the future, ChIP-seq quality assessment similar to that used here could guide experimentalists at early stages in a study, provide useful input in the publication process, and be used to stratify ChIP-seq data for different community-wide uses.

Chromatin immunoprecipitation (ChIP) (Gilmour and Lis 1984; Gilmour and Lis 1985; Solomon *et al.* 1988) experiments identify sites of occupancy by specific transcription factors (TFs), cofactors, and other chromatin-associated proteins as well as histone modifications. Such proteins are concentrated at specific loci via direct binding to DNA or by indirect binding mediated by other proteins or RNA molecules. In most ChIP protocols, proteins are first cross-linked to DNA, most often using formaldehyde. The fixed chromatin is sheared, and an antibody specific for the protein or histone modification of interest is used to retrieve protein:DNA complexes from which the DNA segments are released and then assayed. The assay was first applied to individual TF/promoter complexes by using qPCR to detect enrichment over specific DNA segments (Hecht *et al.* 1996). Subsequent adaptations extended it to large sets of promoters or other genomic regions by using microarrays (ChIP-on-Chip/ChIP-Chip) (Ren *et al.* 2000; Iyer *et al.* 2001; Lieb *et al.* 2001; Horak and Snyder 2002; Weinmann *et al.* 2002). Ultimately, the entire genome became accessible with the advent of high-throughput sequencing and the development of ChIP-seq (Johnson *et al.* 2007; Barski *et al.* 2007; Mikkelsen *et al.* 2007; Robertson *et al.* 2007).

In all cases, preferential enrichment of a given immunoprecipitated DNA segment is detected and quantified by comparing it with a control experiment in which there is no specific antibody enrichment step. These controls can be generated from sonicated DNA before immunoprecipitation (input) or a mock immunoprecipitation with an unrelated antibody (IgG). Sequencing-based ChIP has become the method of choice because it enables genome-wide coverage, even for large genomes, and because of its superior signal-to-noise characteristics compared to alternative methods. Since its initial development, ChIP-seq has been used in hundreds of publications (778 in PubMed as of December 18, 2012), including by the ENCODE consortium (ENCODE Project Consortium 2011; ENCODE Project Consortium 2012), to map occupancy over 100 human TFs and cofactors in a diverse collection of cell lines (Gerstein *et al.* 2012; Wang *et al.* 2012).

A basic question for any ChIP-seq experiment is, how successful was it? It has taken several years for the field to develop objective ways to quantify key aspects of success in immunoprecipitation enrichment, library building, and final sequencing. Poor datasets that have high false-negative rates in peak calling are a predictable pitfall that has significant downstream consequences for some kinds of biological and computational analyses. For example, when lower-quality datasets are used for integrative analyses that are sensitive to false-negative rates, incorrect inferences and conclusions become likely (see *Discussion*). In estimating data quality, the traditional approach of visual inspection at a limited number of sites (often previously well-characterized using low-throughput approaches) is inefficient, subjective, and ultimately can be deceptive. It is also possible (and commonly observed in practice) that sites, the biological importance of which has been defined by independent functional assays, can decrease to below the sensitivity threshold of a poor or mediocre ChIP-seq experiment. Moreover, there is no current way to predict, *a priori*, the number of sites in the genome that should be detectable for a given factor and cell type. Most TFs studied thus far reproducibly occupy thousands to tens of thousands of sites (ENCODE Project Consortium 2012; Landt *et al.* 2012). Thus, a dataset for which several thousand sites have been called might in fact be capturing a minority of true positive interactions, or it might encompass virtually all biologically pertinent sites. To help address the problem of data assessment as part of the ENCODE project, we and others developed a set of ChIP-seq quality control (QC) metrics and guidelines (Landt *et al.* 2012) that were adopted and applied to all of its datasets. Substandard datasets were consequently replaced, flagged as substandard, and/or removed from analysis (ENCODE Project Consortium 2012; Landt *et al.* 2012).

Incorporating published datasets into an ongoing study can bring new biological insights and avoid unnecessary duplication of work. Variable quality of published data can be a significant barrier to these uses of existing data. They are the products of work from many different laboratories with invaluable expertise in specific biological systems, but they also use many variations of ChIP-seq experimental protocols and bioinformatics treatments. The extent and nature of the variations have not been assessed globally and systematically. In this work, we examined the GEO submission series containing vertebrate TF ChIP-seq datasets and found that ∼20% of datasets scored as being of low quality, with an additional ∼25% exhibiting intermediate ChIP enrichment. We also noticed that approximately one-third of studies have control datasets with a high degree of read clustering that is normally expected only in ChIP-seq datasets. This was observed more often for the IgG control design than for input DNA controls. These and related observations argue for data quality measures routine characterization and reporting of ChIP-seq data quality measures.

## Materials and Methods

### Sequencing read alignment

Raw sequencing reads for all non-ENCODE GEO series containing ChIP-seq datasets against TFs and chromatin-modifying proteins (submitted before April 1, 2012) were downloaded from GEO in SRA format and converted to FASTQ format using the fastq-dump program in the sratoolkit (version 2.1.9). Reads were aligned using Bowtie (Langmead *et al.* 2009) version 0.12.7 with the following setting: “-v 2 -t -k 2 -m 1 –best–strata,” which– allows for two mismatches relative to the reference and only retains unique alignments. Human datasets were mapped against the male set of chromosomes (excluding all random chromosomes and haplotypes) for version hg19 of the human genome; the mm9 version of the mouse genome was used for mouse data, rn5 was used for rat data, danRer7 was used for zebrafish data, susScr2 was used for pig data, and xenTro3 was used for the clawed frog *Xaenopus tropicalis* data, and all assemblies were downloaded from the UCSC genome browser (Kent at al. 2002).

### ChIP quality assessment

ChIP quality assessment was performed on both ChIP and input datasets using the general strategy described by Landt *et al.* (2012). Because a library may score as an “unsuccessful ChIP” for reasons other than IP failure (e.g. being performed in a knockout background, in si/shRNA-treated cells, or in conditions under which the factor is not expressed or not bound to DNA), the following additional criteria were used to determine whether each library is expected to score positively in the QC assessment:

1. All experiments claimed to be successful by authors are expected to exhibit high level of read clustering.
2. All inputs (sonicated DNA and IgG mock IPs) are expected to exhibit minimal read clustering (QC tag of −2 or −1).
3. All ChIP-seq experiments performed in a knockout background for the factor are expected to exhibit minimal read clustering (QC tag of −2 or −1).
4. Because knockdown efficiency varies and because it is unknown what protein levels would be sufficiently high for the factor to be successfully ChIP-ed, ChIP-seq experiments performed in cells treated with si/shRNAs targeting the factor are set aside as “unknown” and assessed for library complexity and sequencing depth but not for ChIP quality.
5. Experiments against factors known to bind to DNA on some stimulus performed in unstimulated cells are also tagged as “unknown” because lower-level binding in unstimulated cells cannot be ruled out (and is, in fact, often observed).
6. Experiments performed in conditions that may result in the factor not binding to DNA (time courses, knockdowns, or knockouts for other factors that may affect binding of the targeted factor) are also tagged as “unknown.”
7. Other experiments not matching any of these categories are expected to exhibit high levels of read clustering.

Cross-correlation analysis was performed using version 1.10.1 of SPP (Kharchenko *et al.* 2008) and the following parameter: “−s = 0:2:400.” QC scores were assigned based on the relative strand correlation (RSC) values (integers ranging from −2 to −2, *RSC* ∈ {0, 0.25} ⇒ *QC* ← −2, *RSC* ∈ {0.25, 0.50} ⇒ *QC* ← −1, *RSC* ∈ {0.50, 1.00} ⇒ *QC* ← 0, *RSC* ∈ {1, 1.50} ⇒ *QC* ← +1, *RSC* ≥ 1.5 ⇒ *QC* ← +2, with −2 corresponding to minimal read clustering and 2 corresponding to a highly clustered library) and used as a measure of ChIP quality. These scores capture the extent of read clustering in a ChIP-seq experiment in organisms whose genomes have similar size and structure to those of mammals. We point out that these scores may not be appropriate in genomes with very different size and/or structure. This motivated us to discard data from nonvertebrate model organisms for this analysis. Different values than those used here for RSC or normalized strand correlation (NSC) coefficients may be needed for such genomes, and this is a topic for future investigation. Cross-correlation plots were manually examined to ensure no artifactual QC scores were included because of size selection issues (such as, for example, a library being fragmented to an average size close to the read length and confusing the automated fragment peak assignment). In general, we recommend manual examination of cross-correlation plots in all cases. This presents a deeper and more detailed view of the characteristics of the dataset because the cross-correlation profile provides not only information regarding ChIP enrichment but also regarding the fragment length distribution in the datasets. For example, a dataset might exhibit periodicity in the distribution of fragment size lengths, presenting itself as numerous smaller peaks along the curve (often seen when chromatin is enzymatically digested rather than sonicated), or it can deviate from the standard unimodal pattern (aside from the phantom peak) indicating issues with size selection. The code for running SPP and assigning QC scores is available at https://code.google.com/p/phantompeakqualtools/.

### MyoD and myogenin ChIP-seq peak calling

MyoD and myogenin datasets were generated by the Wold laboratory and are available under GEO accession number GSE44824. We note that the apparent weakness of the “myogenin 2” ChIP dataset is most likely attributable to undersequencing and would be elevated to high-quality status if sequenced deeper; undersequencing is one possible reason for suboptimal quality metrics (A. Kundaje *et al.*, unpublished data). Reads were mapped as described above and peaks were called using ERANGE3.2 (Johnson *et al.* 2007) with the following settings: “−minimum 2 −ratio 3 −shift learn −revbackground −listPeak.” ChIP-seq peak calls were counted as overlapping if their summits were within 200 bp of each other. Read mapping statistics and QC metrics for these datasets can be found in Supporting Information, Table S2.

## Results

### Dataset collection, data processing, and quality metrics

We downloaded all GEO series containing ChIP-seq datasets for vertebrate TFs or chromatin-modifying and remodeling proteins, along with their corresponding control libraries, submitted before April 1, 2012. We excluded ENCODE datasets because they have previously been subjected to this quality assessment (ENCODE Project Consortium 2012). We provide here a summary of ENCODE TF ChIP-seq data quality from the two main production groups in Figure S9 and Figure S10 (Landt *et al.* 2012).

For several reasons, we also excluded histone modifications and RNA Polymerase II datasets. First, in our experience, ChIP-seq against these targets is very robust to experimental variation and the success rate is reliably high (provided the antibody reagents used are of high quality). Second, an especially large proportion of published data are for histone marks. The effect of including all of these in the survey is to obscure or skew what is happening in the information-rich sample set that includes diverse TFs and cofactors. Finally, the currently available QC metrics were designed and are best suited for TF data that produce highly localized “point-source” occupancy (as they quantify the extent of read clustering in the genome). This means that the metrics themselves need to be interpreted differently if they are applied to, for example, repressive histone marks such as H3K9me3 and H3K27me3, which form large “broad-source” regions of enrichment (Pepke *et al.* 2009). Arguably, these data will need their own metrics and this will be a challenge for the future.

The final collection of datasets contained 191 GEO series containing a total of 917 ChIP-seq and 292 control libraries. Except for a limited number of cases in which a GEO series was associated with multiple publications, two or three GEO series were associated with the same publication, or a GEO series has not yet been used in a publication, and there is a one-to-one relationship between GEO series and published articles in the literature (Robertson *et al.* 2007; Chen *et al.* 2008; Marson *et al.* 2008; Bilodeau *et al.* 2009; Cheng *et al.* 2009; De Santa *et al.* 2009; Lister *et al.* 2009; Nishiyama *et al.* 2009; Visel *et al.* 2009; Welboren *et al.* 2009; Wilson *et al.* 2009; Yu *et al.* 2009; Yuan *et al.* 2009; Barish *et al.* 2010; Blow *et al.* 2010; Blow *et al.* 2010; Cao *et al.* 2010; Chi *et al.* 2010; Chia *et al.* 2010; Chicas *et al.* 2010; Corbo *et al.* 2010; Cuddapah *et al.* 2009; Durant *et al.* 2010; Fortschegger *et al.* 2010; Gotea *et al.* 2010; Gu *et al.* 2010; Han *et al.* 2010; Heinz *et al.* 2010; Heng *et al.* 2010; Ho *et al.* 2009; Hollenhorst *et al.* 2009; Hu *et al.* 2010; Johannes *et al.* 2010; Jung *et al.* 2010; Kagey *et al.* 2010; Kassouf *et al.* 2010; Kim *et al.* 2010; Kong *et al.* 2010; Kouwenhoven *et al.* 2010; Krebs *et al.* 2010; Kunarso *et al.* 2010; Kwon *et al.* 2009; Law *et al.* 2010; Lee *et al.* 2010; Lefterova *et al.* 2010; Li *et al.* 2010; Lin *et al.* 2010; Liu *et al.* 2010; Ma *et al.* 2010; MacIsaac *et al.* 2010; Mahony *et al.* 2010; Martinez *et al.* 2010; Palii *et al.* 2010; Qi *et al.* 2010; Rada-Iglesias *et al.* 2010; Rahl *et al.* 2010; Ramagopalan *et al.* 2010; Ramos *et al.* 2010; Schlesinger *et al.* 2010; Schnetz *et al.* 2010; Sehat *et al.* 2010; Steger *et al.* 2010; Tallack *et al.* 2010; Tang *et al.* 2010; Vermeulen *et al.* 2010; Verzi *et al.* 2010; Vivar *et al.* 2010; Wei *et al.* 2010; Woodfield *et al.* 2010; Yang *et al.* 2010; Yao *et al.* 2010; Yu *et al.* 2010; An *et al.* 2011; Ang *et al.* 2011; Bergsland *et al.* 2011; Bernt *et al.* 2011; Botcheva *et al.* 2011; Brown *et al.* 2011; Bugge *et al.* 2011; Ceol *et al.* 2011; Ceschin *et al.* 2011; Costessi *et al.* 2011; Ebert *et al.* 2011; Fang *et al.* 2011; Handoko *et al.* 2011; He *et al.* 2011; Heikkinen *et al.* 2011; Holmstrom *et al.* 2011; Horiuchi *et al.* 2011; Hu *et al.* 2011; Joseph *et al.* 2010; Kim *et al.* 2011; Klisch *et al.* 2011; Koeppel *et al.* 2011; Kong *et al.* 2011; Little *et al.* 2011; Liu *et al.* 2011; Lo *et al.* 2011; Marban *et al.* 2011; Mazzoni *et al.* 2011; McManus *et al.* 2011; Mendoza-Parra *et al.* 2011; Meyer *et al.* 2012; Miyazaki *et al.* 2011; Mullen *et al.* 2011; Mullican *et al.* 2011; Nakayamada *et al.* 2011; Nitzsche *et al.* 2011; Norton *et al.* 2011; Novershtern *et al.* 2011; Quenneville *et al.* 2011; Rao *et al.* 2011; Rey *et al.* 2011; Sahu *et al.* 2011; Schmitz *et al.* 2011; Seitz *et al.* 2011; Shen *et al.* 2011; Shukla *et al.* 2011; Siersbæk *et al.* 2011; Smeenk *et al.* 2011; Smith *et al.* 2011; Soccio *et al.* 2011; Stadler *et al.* 2011; Sun *et al.* 2011; Tan *et al.* 2011a; Tan *et al.* 2011b; Teo *et al.* 2011; Tijssen *et al.* 2011; Tiwari *et al.* 2011a; Tiwari *et al.* 2011b; Trompouki *et al.* 2011; van Heeringen *et al.* 2011; Verzi *et al.* 2011; Wang *et al.* 2011a; Wang *et al.* 2011b; Wei *et al.* 2011; Whyte *et al.* 2011; Wu *et al.* 2011a; Wu *et al.* 2011b; Xu *et al.* 2011; Yang *et al.* 2011; Yildirim *et al.* 2011; Yoon *et al.* 2011; Zhang *et al.* 2011; Zhao *et al.* 2011a; Zhao *et al.* 2011b; Avvakumov *et al.* 2012; Barish *et al.* 2012; Boergesen *et al.* 2012; Bugge *et al.* 2012; Canella *et al.* 2012; Cardamone *et al.* 2012; Cheng *et al.* 2012; Chlon *et al.* 2012; Cho *et al.* 2012; Doré *et al.* 2012; Fan *et al.* 2012; Feng *et al.* 2011; Fong *et al.* 2012; Gao *et al.* 2012; Gowher *et al.* 2012; Hunkapiller *et al.* 2012; Hutchins *et al.* 2012; Li *et al.* 2012; Lu *et al.* 2012; Miller *et al.* 2011; Ntziachristos *et al.* 2012; Pehkonen *et al.* 2012; Ptasinska *et al.* 2012; Remeseiro *et al.* 2012; Sadasivam *et al.* 2012; Sakabe *et al.* 2012; Schödel *et al.* 2012; Trowbridge *et al.* 2012; Vilagos *et al.* 2012; Wu *et al.* 2012; Xiao *et al.* 2012; Yu *et al.* 2012; unpublished at the time of completion of this manuscript are the following GEO accession numbers: GSE33346, GSE33850, GSE36561, GSE30919, GSE33128, GSE35109, GSE25426, GSE31951, GSE26711, GSE23581, GSE26136, GSE26680, GSE15844, GSE21916, GSE22303, and GSE29180; direct links to all GEO series can be found in Table S1).

We discuss IgG and input controls separately because, to the best of our knowledge, any potential general differences between the two types of controls have not been investigated systematically in the context of ChIP-seq (Peng *et al.* 2007 addressed these questions for ChIP-Chip data; however, the nature of the background is substantially different for microarrays).

We mapped all reads with uniform settings (see *Materials and Methods* for details) and examined library and ChIP QC metrics for each dataset. These criteria have already been discussed by Landt *et al.* (2012), and a detailed treatment of cross-correlation is presented elsewhere (Kundaje *et al.*, unpublished data). Here, we provide a brief overview of each.

#### Sequencing depth:

If a ChIP-seq experiment achieves successful immune enrichment and the resulting library adequately represents the sample, then greater sequencing depth will produce a more complete map of TF occupancy (Landt *et al.* 2012). At a greater depth, the measurement will identify a larger number of reproducible sites containing the corresponding DNA-binding sequence motif. Undersequencing of an otherwise successful library will lead to false-negative results. It has been difficult to establish a universal minimal sequencing depth because of differences between factors. Any threshold is going to be somewhat arbitrary but, in general, the major cost/benefit trade-off is between sequencing individual samples more deeply and generating more replicates; for most contemporary purposes, an independent duplicate measurement of 12 million reads arguably adds greater overall value than a single determination with 24 million reads, even though the higher number of reads will increase sensitivity. The number of mapped reads less than 1–2 million for a typical TF will usually be inadequate for capturing the complexity of an interactome for a mammalian-size genome. Many datasets now in the public domain were generated when sequencing throughput was lower than it is now and costs were higher (between 2007 and 2013, sequencing throughput has increased by approximately two orders of magnitude). As a consequence, many early ChIP-seq libraries were sequenced to a depth of only a few million reads. We therefore divided datasets into sequencing bins by using thresholds of 1 million, 5 million, 12 million, and 24 million uniquely mapped reads (taking into account sequencing depths recommended in the past by the ENCODE consortium for TFs). Libraries having less than 1 million reads are considered severely undersequenced, and those with more than 12 million are considered reasonably deeply sequenced.

#### Library complexity:

A second characteristic that influences the quality of a ChIP-seq measurement is the sequence fragment diversity of the sequencing library. This is often referred to as library complexity, and low complexity is undesirable, although we note that much better IP enrichment than what is now obtained could, in the future, lead to very high-quality datasets with low library complexity. Currently, low-complexity libraries mainly result from experimental deficiencies: either too few starting molecules at the end of the immunoprecipitation step or inefficient steps in subsequent library building. As a result, the same starting molecules are sequenced repeatedly. Very-low-complexity libraries will not contain enough information to effectively sample the true positive occupancy sites and they distort the signal position and intensity. This can confuse peak callers (especially if the algorithm does not collapse presumptive PCR duplicates), leading to peak calling artifacts (Landt *et al.* 2012). We calculate the following metric as an indicator of library complexity (Landt *et al.* 2012):

$$\text{Library}\;\text{complexity}=\frac{\text{Number}\;\text{positions}\;\text{in}\;\text{the}\;\text{genome}\;\text{with}\;\text{uniquely}\;\text{mappable}\;\text{reads}\;\text{in}\;\text{dataset}}{\text{Number}\;\text{uniquely}\;\text{mappable}\;\text{reads}\;\text{in}\;\text{dataset}}$$ (1)

**Figure 1:**
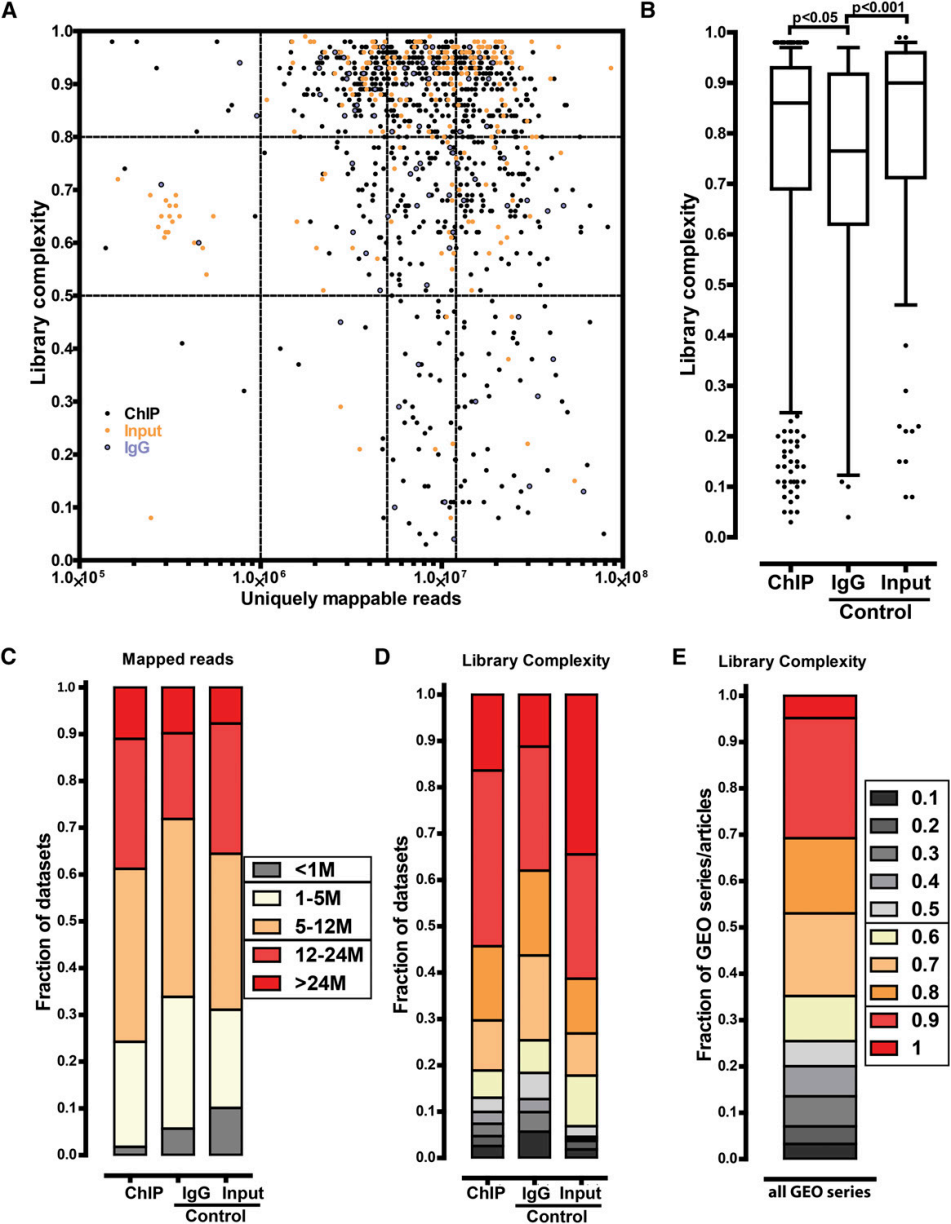
Sequencing library characteristics. (A) Joint distribution of library complexity and sequencing depth for all datasets examined. Vertical lines are drawn at 1 million, 5 million, and 12 million reads. Horizontal and vertical lines indicate quality classes discussed in the text. The upper right domain (number of uniquely mappable reads ≥12 million and library complexity ≥0.8) passes current quality thresholds. (B) Distribution of library complexity for ChIP-seq datasets, IgG controls, and inputs. (C) Distribution of sequencing depth for ChIP-seq datasets, IgG controls, and sonicated inputs. (D) Fraction of ChIP-seq, IgG, and input datasets exhibiting high, medium, and low complexity. (E) Fraction of studies containing libraries of high, medium, and low complexity (the distribution of the minimum library complexity observed is shown)

Estimated in this simple way, library complexity is expected to decrease eventually with increased sequencing depth because even highly complex libraries become exhausted by very deep sequencing. Reduced apparent complexity would also be observed with extremely successful ChIP-seq experiments for TFs that bind to the genome in a highly discriminative fashion to a limited number of locations. In such libraries, the majority of reads would originate from the limited genomic subspace around binding sites, resulting in low library complexity. With current methods, this is a largely theoretical consideration; in practice, in most ChIP-seq libraries only a minority of reads originates from factor-bound sites, with the rest (the majority) representing genomic background. Because the majority of libraries we examined were in the sequencing depth range over which these values represent library complexity reasonably well (Figure 1A and Figure S2), we separated datasets into the following complexity groups: high complexity (apparent library complexity ≥.8); medium to low complexity (apparent library complexity between 0.5 and 0.8); and very low complexity (apparent library complexity ≤.5). We also note that in substantially smaller genomes, the apparent library complexity is expected to be lower because the number of positions from which sequencing library fragments can originate is smaller.

#### Cross-correlation analysis of read clustering and ChIP enrichment:

Because the majority of sequencing reads in a ChIP-seq library represent nonspecific genomic backgrounds, these reads are expected to be distributed randomly over the genome, to a first approximation. In contrast, reads originating from specific occupancy events cluster around the sites of protein–DNA interactions, where they are distributed in a characteristic asymmetric pattern on the plus and minus strands (Kharchenko *et al.* 2008). Cross-correlation analysis is an effective way of measuring the extent of this clustering. It also captures additional global features of the data, such as the average fragment length and fragment length distribution (Kharchenko *et al.* 2008; Landt *et al.* 2012). Specifically, the read coverage profiles on the two strands are shifted relative to the other over a range of shift values and the correlation between the profiles is calculated at each shift (Kharchenko *et al.* 2008). The resulting plot has one (“phantom”) peak corresponding to the read length and another peak corresponding to the average fragment length; the height of the fragment-length peak is highly informative of the extent of read clustering in the library and, in turn, of the success of a ChIP-seq experiment. This feature is best captured by the NSC and RSC metrics discussed by Landt *et al.* (2012).

We applied SPP (Kharchenko *et al.* 2008) to perform cross-correlation analysis for all libraries in our survey. We then used the RSC cross-correlation metric to assign integer QC tag values in the {−2, 2} range to datasets, with QC values of 2 corresponding to very highly clustered (and most likely, also successful) datasets and QC values of −2 to datasets exhibiting no to minimal read clustering; negative values are expected for input datasets. The RSC metric captures well the extent of read enrichment in vertebrate genomes similar in size and structure to humans, which this study focuses on. We provide representative examples of cross-correlation plots for each of the five QC categories in Figure S1A, and we use these tags as convenient general proxies for ChIP quality throughout the following analysis. We note that the discretization thresholds are not intended to be absolute determinants of quality, but they do enable one to rapidly scan very large numbers of datasets. In practice, examining the cross-correlation plots and the continuously distributed NSC and RSC values and using those together with information about sequencing depth and library complexity are always more informative and can provide valuable nuances for understanding specific datasets. Direct examination of plots allows one to detect datasets with odd cross-correlation profiles (we show a few representative examples in Figure S11). It is possible in theory for low-complexity libraries to produce artificially high cross-correlation scores if stacks of reads on opposite strands are located close to each other in regions of enrichment; however, the Pearson correlation between library complexity scores and RSC values in the collection of ChIP datasets surveyed here was 0.0084, indicating that such cases do not feature significantly in this analysis.

An additional major component of the ChIP-seq QC pipeline developed by the ENCODE consortium is reproducibility analysis of replicates, based on the irreproducible discovery rate (IDR) statistic (Li *et al.* 2011). However, because many of the studies we surveyed did not have replicates, we only evaluated datasets on the level of individual experiments. Single dataset evaluation is almost always a valuable precursor to evaluation of replicates because, typically, a second replicate is generated after a successful first one. The full list of datasets, mapping, and QC statistics is provided in Table S1.

### Sequencing depth and library complexity

Figure 1A shows the distribution of sequencing depth and library complexity for ChIP-seq and control datasets. The upper right domain, bounded by 12 million reads per sample and a complexity value of 0.8, is an arbitrary but useful definition of high quality according to these measures. A majority of datasets had reasonably good complexity and severely undersequenced libraries were rare (Figure 1C). A minority (38.8%) of datasets had more than 12 million mapped reads; however, as discussed, this is not unexpected, because a large fraction of the datasets we surveyed were generated in times of significantly higher sequencing cost and lower throughput. Strikingly, the median complexity of IgG control datasets was less than 0.8 and considerably lower than that of either ChIP-seq or sonicated input libraries (Figure 1B). This is not a result of IgG datasets having been sequenced much more deeply than the other two groups; in fact, the median sequencing depth of IgG controls is lower (Figure S2). The concern that some individual IgG inputs might provide insufficient DNA mass to build highly complex libraries has been raised before (Landt *et al.* 2012), and our observations are consistent with this, although it is not a characteristic of all IgG controls.

Slightly more than half (54.3%) of ChIP-seq datasets had library complexity more than 0.8, whereas very-low-complexity (< 0.5) libraries comprised 12.9% of datasets; the fraction of very-low-complexity libraries was higher and lower for IgG and input datasets, respectively (Figure 1D). Because most GEO series contained multiple libraries, we also asked, how common is the presence of low-complexity libraries in individual studies? Figure 1E shows the distribution of the minimum library complexity in each such series (for all types of datasets). One-quarter (25.4%) of all studies contained very-low-complexity libraries.

### Cross-correlation quality assessment of ChIP-seq datasets

Next, we examined the distribution of SPP QC scores for ChIP-seq datasets. Before doing this, we excluded a minority of datasets for which there was a good reason to think high ChIP enrichment should not be expected. For example, experiments executed in knockouts, knockdowns, or settings in which the factor is not expressed are not expected to produce a high-scoring measurement. And in a few cases, the factor in question might be known to bind to only a small number of sites in the genome; this has been proposed, for example, for some ZNF TFs and Pol3 and its associated factors (Landt *et al.* 2012). Our detailed criteria for inclusion are described in *Materials and Methods*.

**Figure 2:**
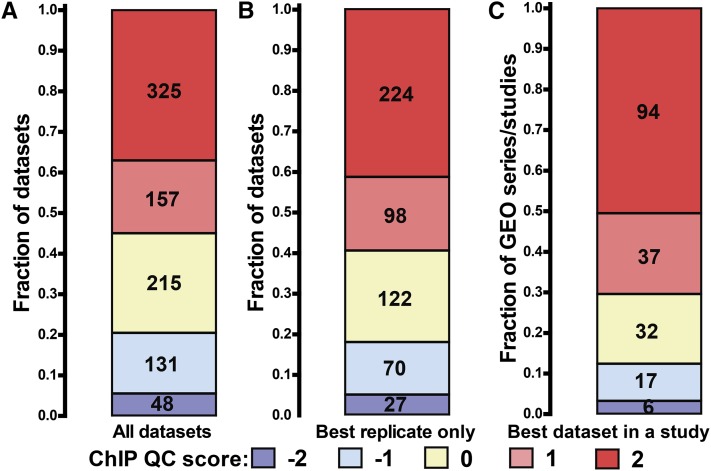
ChIP QC assessment summary. The numbers in each box indicate the total number of datasets/studies belonging to it. SPP QC scores of +1 and +2 indicate a high degree of read clustering in a dataset. (A) Distribution of SPP QC scores for all ChIP-seq datasets examined. (B) Distribution of SPP QC scores for the best replicates for a factor/condition combination in each study. (C) Distribution of the maximum SPP QC scores for all ChIP-seq datasets in a study.

Figure 2A shows the QC score distribution for all ChIP-seq datasets we retained. Strikingly, only 55% (482 out of 876) of datasets had QC scores of 1 or 2, *i.e.*, they were likely to be highly successful. An additional 24.5% (215 out of 876) had a score of 0, indicating that they were of intermediate quality, and 20.4% (179 out of 876) had low-quality scores of −1 and −2. Sometimes multiple replicates for a factor were submitted but only one scored poorly, so we also compiled a second set of ChIP-seq experiments that only included the best available replicate for each factor and condition (Figure 2B). This set included 322 datasets (59%) with QC scores of 2 or 1. The fraction of intermediate-quality or low-scoring datasets in this group decreased as expected. However, the decrease was modest with 18% (97 out of 541) of the best available replicates scoring −1 or −2, and 22.5% (122 out of 541) scoring 0.

**Figure 3:**
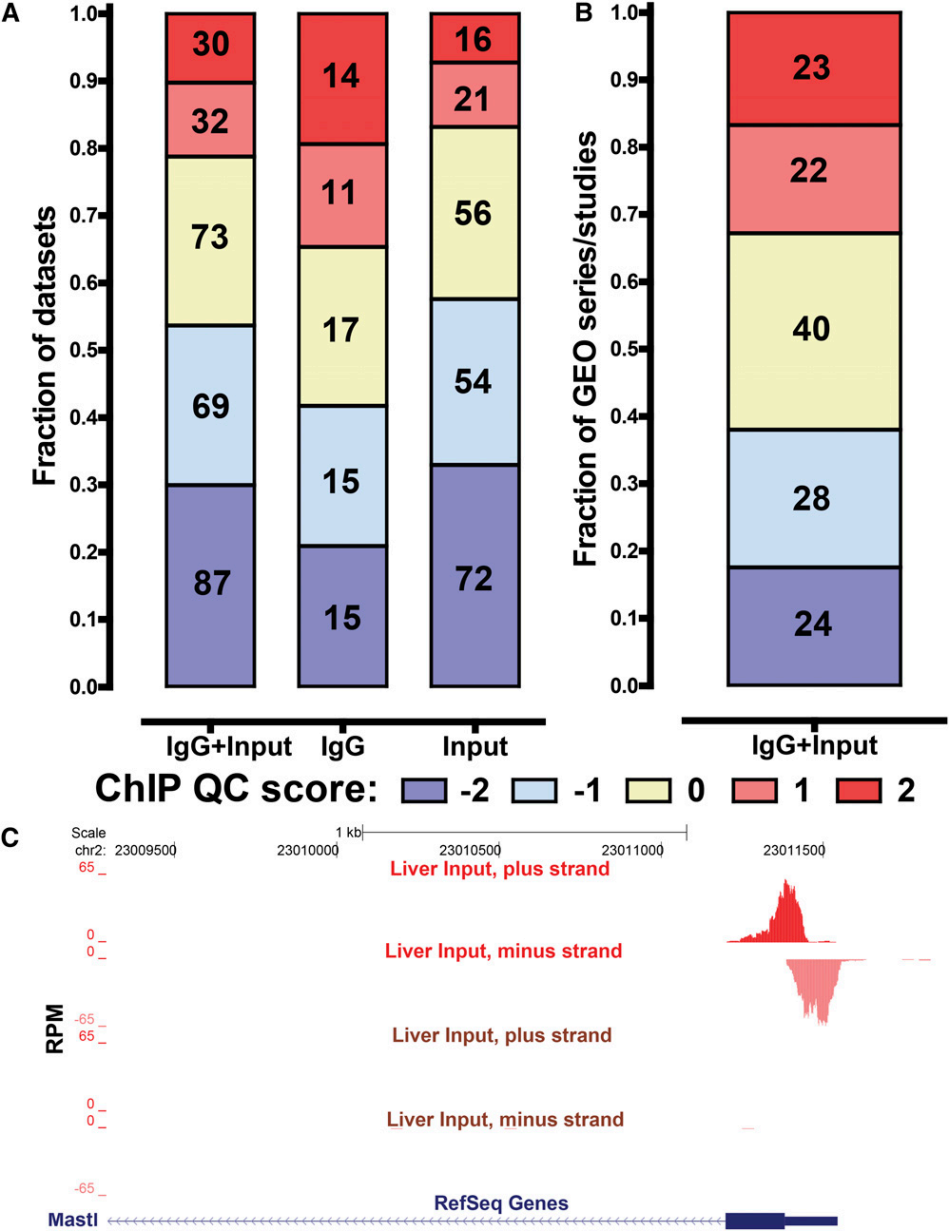
Assessment of read clustering in control datasets. The numbers in each box indicate the total number of datasets/studies belonging to it. SPP QC scores of 1 and 2 indicate a high degree of read clustering in a dataset. (A) Distribution of SPP QC scores for all control datasets (IgG + input), IgG/mock IP controls (IgG), and sonicated inputs (inputs). (B) Fraction of studies containing highly clustered inputs. The distribution of the maximum SPP QC score for all inputs in a dataset is shown. (C) Examples of a highly clustered input [mouse liver, upper two tracks, (MacIsaac *et al.* 2010), QC score of 2] and an input that does not show high extent of read clustering [mouse liver, lower two tracks (Soccio *et al.* 2011), QC score of −1). The promoter of the *MASTL* gene is shown. All tracks are shown to the same scale and reads mapping to the plus and minus strands are displayed separately for better visualization of the cross-correlation between the two.

We then examined the distribution of the maximum QC score for each study, regardless of the target identity (Figure 3C). The fraction of low scores decreased further, though only 70.4% of studies (131 out of 186) had a score of 1 or 2 for their best experiment. Finally, we compiled a list of the top-scoring datasets from all studies that assayed only a single TF; 19.7% (19 out of 96) of these studies had scores of −1 or −2, 25% (24 of 96) had a score of 0, and 55.2% (53 of 96) were marked as likely to be successful, with scores of 1 and 2 (Figure S3C).

### Read clustering in control datasets

Control datasets serve the important purpose of helping to distinguish read enrichment attributable to the immunoprecipitation step from artifactual read clustering attributable to other experimental factors, both known and unknown. It is, for example, well-appreciated that differential chromatin shearing efficiency can lead to the overrepresentation of areas of open chromatin (usually immediately surrounding transcribed promoters) in sequencing libraries. This has been termed the “Sono-seq” effect when attributed to sonication (Auerbach *et al.* 2009). In addition, unknown copy number variants relative to the reference genome or sequence composition biases can give false-positive occupancy calls. In particular, specifics of the amplification step in sequencing platforms can introduce bias due to GC content (Ho *et al.* 2011).

In general, control datasets are not expected to exhibit a pattern of significant read clustering similar in strength to that of successful ChIP-seq datasets. In our own practice, under standard cross-linking protocols, most do not. However, we noticed that a minority of control datasets produce positive ChIP QC metric scores along with prominent cross-correlation peaks. Figure S1B shows examples of cross-correlation plots for individual control datasets with all possible QC scores, from −2 to 2, and Figure 3C shows a browser snapshot of a region with strong read enrichment in a highly clustered (QC score of 2) input library. No such enrichment was observed in a different control library from a similar biological source having a QC score of −1.

We asked how general this phenomenon is by examining the distribution of QC scores of both IgG and input control datasets (Figure 3A). Surprisingly, only 53.6% (156 out of 291) of control datasets had QC scores of −2 or −1 and 25% (73 of 291) had a score of 0, whereas 21.3% (62 of 291) exhibited a very high degree of read clustering and received scores of 1 or 2. The highly clustered inputs were notably more common among IgG controls than among input chromatin controls (Figure 3A). Moreover, high read clustering was more often found in low-complexity libraries (which are themselves more common among IgG controls) (Figure S4, A and B).

We also examined how widespread control sample clustering is on the level of individual GEO series/studies to see if the phenomenon is restricted to a few larger studies. Figure 3B shows the distribution of the maximal control sample QC score for all studies. Of the studies for which control datasets were available, 32.8% (45 of 123) contained at least one highly clustered control with a score of 1 or 2, and 29.2% (40 of 123) contained a control with a score of 0. Thus, control datasets surprisingly often exhibit a high extent of read clustering similar to that of ChIP-seq datasets. This is even more striking considering that formaldehyde-assisted isolation of regulatory elements (FAIRE-seq) data (an assay that is based on the preferential enrichment of open chromatin in sonicated DNA and aims to achieve high read clustering) from ENCODE usually have QC scores between −2 and 0, Moreover, the Sono-seq datasets published by Auerbach *et al.* (2009) all have scores of −2.

We note that unless this effect is very strong and is associated with notable genomic features such as promoters of genes, it can be difficult to detect by the usual methods of visual inspection of signal tracks on a genome browser. It is, however, readily apparent in cross-correlation analysis and our results raise awareness of its existence. As mentioned, one candidate explanation for this phenomenon is the previously described “Sono-seq” effect. Using standard experimental protocols, this effect has been rare in our experience; however, under more aggressive cross-linking conditions, we have observed increased read clustering in control samples (Figure S5). Notably, the original “Sono-seq” description focused on promoter regions, but we have also observed it over distal regulatory elements, where its strength was even higher than at promoters (Figure S5). Thus, variation in the extent of fixation, as well as sonication, might be a substantial contributor to variation in read clustering across the broader data collection. Another potential contributing factor is sequencing depth. Although the average sequencing depth for highly clustered IgG and input controls is higher than that of controls with negative QC scores (Figure S4, C and D) this by no means explains all the clustering observed in controls. There are many examples of more deeply sequenced input and IgG libraries with no significant cross-correlation peaks and very few of them were sequenced especially deeply (only eight control libraries had >4 × 10^7^ reads not desirable. Finally, “Sono-seq” need not be the only explanation. Whereas a number of control datasets with QC scores of 2 exhibited higher read coverage around promoters, others did not (Figure S6), suggesting at least one additional source of unexplained read enrichment in control samples. Because rich annotation of functional genomic elements outside promoter regions was not available for many cell types in our survey, this phenomenon is a subject for future analyses.

## Discussion

We performed a systematic survey of ChIP quality for publicly available vertebrate ChIP-seq datasets and found that more than half score as high quality by our measures. This group comprises a set that we believe can be used with confidence for integrative analyses. This conclusion carries the important caveat that we could not assess the specificity of the immune reagents used to perform the experiments. which powerfully affects the biological meaning of the data.

A substantial minority of published datasets (between 20% and 45% of those examined) were of low or intermediate quality by our metrics. This was true not only for individual libraries but also for the best replicates from each study. In addition, we observed a substantial number of low-complexity datasets and an unexpected group of highly clustered control datasets. These observations underscore the widespread variation in published ChIP-seq data. They also raised questions about which kinds of conclusions in primary publications are more or less sensitive to these aspects of data quality. In particular, global quality analysis is useful for guiding subsequent re-use of published data that require higher quality than was needed or achieved in the source study.

Data quality varied widely across “impact” levels. We separated datasets into groups according to the 2011 Thomson Reuters Impact Factor for the journal in which the corresponding article was published and examined the distribution of QC scores in each group (Figure S8). The group with highest impact factor (≥25) contained the largest fraction of datasets with a low QC score of −2 or −1. We also examined the distribution of QC scores with respect to the year of publication and found that the fraction of datasets with low scores has stabilized in the past 3 yr at approximately 20% (Figure S7).

We emphasize that datasets scoring as low quality by the metrics used here can, nevertheless, produce important biological discoveries. For this reason, it would be an error to set a rigid “standard” that every published dataset must meet. Instead, routine QC analysis can make it easy to see when there is reason for concern about a given dataset. It can also provide a first tier of guidance about what uses are likely to be appropriate for a given dataset. As discussed previously, the appropriate level of QC stringency depends on the specific goals of the experiment and methods of analysis (Landt *et al.* 2012). In particular, some analyses that are sensitive to false-negative results are particularly vulnerable to inclusion of low-scoring datasets. For example, trying to derive combinatorial TF occupancy rules is seriously compromised and even misleading if a subset of the datasets included is suboptimal.

**Figure 4:**
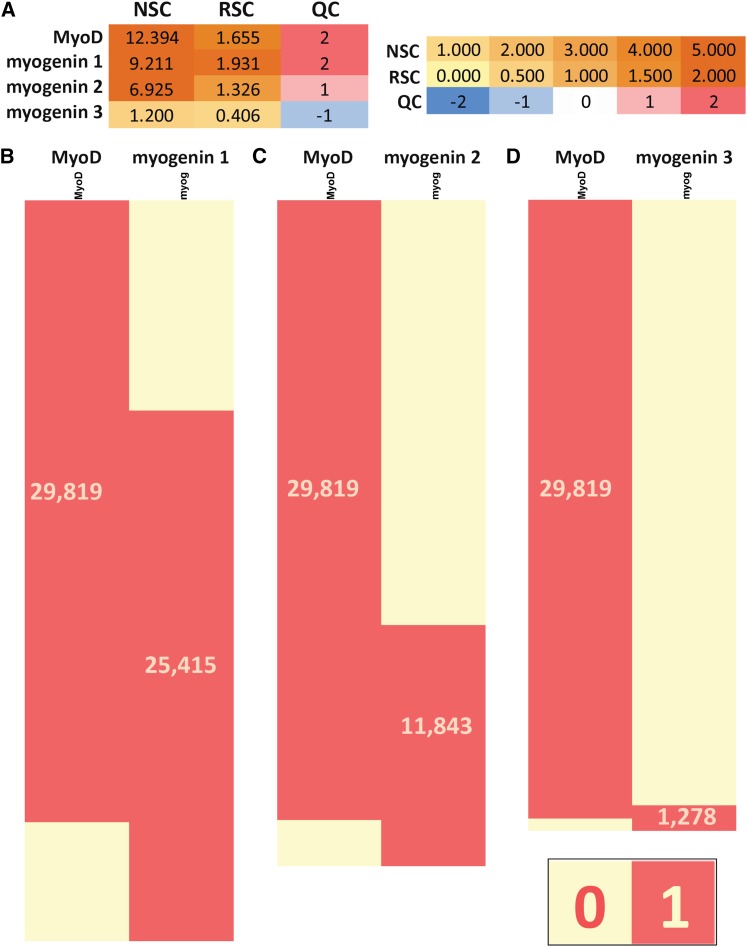
Effect of suboptimal datasets on combinatorial occupancy analysis. The muscle-regulatory factors MyoD and myogenin were assayed in C2C12 myocytes at 60 hr after differentiation. Shown are a single, highly successful MyoD ChIP-seq dataset and three myogenin ChIP-seq datasets, one of which is similarly highly successful (“myogenin 1”), a second weaker one (“myogenin 2”), and a third one that is an experimental failure (“myogenin 3”). (A) Quality control metrics. (B, C, D) The extent of overlap of MyoD and myogenin-binding sites as determined using each of the three myogenin datasets (see *Materials and Methods* for data processing details). MyoD and myogenin are mostly found to bind to the same sites when interactome determinations of comparable strength are used. (B) A sizable group of apparently MyoD-only sites emerges when the medium-strength myogenin dataset is used because of a large number of false-negative myogenin calls. (C) Finally, the unsuccessful myogenin ChIP reveals that most MyoD are not shared by myogenin. (D) Numbers listed in the red blocks corresponding to each set of peak calls indicate size.

We illustrate this with a simple example from our own experience (Figure 4). The MyoD and myogenin TFs are well-known regulators of muscle differentiation (Yun and Wold 1996) and C2C12 cells (Yaffe and Saxel 1977) have been widely used to study the process because they can be propagated in an undifferentiated myoblast state and easily induced to differentiate into myocytes and myotubes. We have performed several ChIP-seq experiments with these factors in differentiated and undifferentiated C2C12 cells (G. DeSalvo *et al.*, unpublished data; A. Kirilusha *et al.*, unpublished data; K. Fisher-Aylor *et al.*, unpublished data), some of which have been highly successful, whereas others were of poor or intermediate quality. Here, we examined the effect of weaker ChIP-seq datasets on combinatorial occupancy analysis using a MyoD ChIP-seq dataset with very high QC metrics and three myogenin datasets with very high, moderately good, and very low metrics (Figure 4A). Using the best myogenin dataset, we found a high degree of overlap between the binding sites of the two factors (Figure 4B). When the medium-quality myogenin dataset was used instead, a sizable group of MyoD-only sites emerged (Figure 4C) and the erroneous conclusion that a substantial number of MyoD sites lack myogenin binding could be reached if this was the only dataset available for analysis. Finally, the poor-quality myogenin dataset contains very few called peaks and, as a result, almost all MyoD sites show no myogenin binding when it is used for analysis (Figure 4D).

Recently, IDR analysis of replicate datasets (Li *et al.* 2011; ENCODE Project Consortium 2012; Landt *et al.* 2012) emerged as a robust method for deriving lists of reproducible occupancy sites from ChIP-seq datasets. IDR is based on differences in the consistency of ranking (usually by signal strength as measured by read enrichment or by statistical significance) for all identified peaks in a pair of ChIP-seq replicates. A virtue of this approach is that it allows a statistically robust set of binding sites to be derived largely independent of thresholds and settings specific to a particular peak-calling algorithm. Ideally, IDR would be used in conjunction with the quality metrics used here (ENCODE Project Consortium 2012; Landt *et al.* 2012). However, replicate measurements do not exist for many of the datasets in our survey of the historic. We expect that IDR will become common practice as sequencing costs decline. Even when that happens, measurements of the quality of individual datasets will remain important because they capture specific information in addition to reproducibility and because IDR analysis is sensitive to the presence of poor-quality replicates. An asymmetric pair consisting of one high-quality and one poorer-quality dataset is dominated in IDR by the weaker replicate, resulting in a shorter list of sites and a high false-negative rate. Care should be exercised in such cases. Although the best approach is to obtain a second high-quality replicate, but if this is not possible, special strategies for treating asymmetric replicates have been devised (Landt *et al.* 2012).

The most perplexing observation was that a subset of control datasets have extensive read clustering in the same range as successful ChIP-seq experiments. In our own practice, we have rarely encountered such libraries and, to the best of our knowledge, there has been no extensive treatment of this issue or its influence on data analysis in the literature. The phenomenon occurred more frequently in IgG controls than in input chromatin controls, although it is by no means limited to the former. In theory, an IgG control should be a superior representation of the true background noise in a ChIP-seq sample because it incorporates biases introduced by the entire immunoprecipitation process, in addition to any enrichments or biases created by chromatin shearing. Using this logic, a simple interpretation is that high read clustering in these controls correctly identifies artifacts in the IP process. When high background sample clustering is observed in control sample, we suggest that it merits immediate investigation of its replicability and its impact on peak-calling for the corresponding ChIP. samples. The fact that we also observed a large number of IgG controls (Figure 3A) that showed no such clustering, argues that this is not a general feature.

A crucial issue is the extent to which clustering in controls is also present as experimental noise in ChIP libraries from the same material. In other words, how well-matched are the control samples with the corrresponding experimental samples, and how robust are the controls? For example, a very strong Sono-seq effect in a control sample is expected to give ChIP-seq libraries with high read clustering that is a combination of true ChIP (antibody-specific) signal plus Sono-seq-derived noise that covers promotors and enhancers in a nonspecific manner. Whereas most contemporary peak callers normalize for enrichment in controls, very strong background noise will diminish the signal-to-noise ratio and adversely affect sensitivity. How severely this affects the results will depend on the overlap between true factor occupancy sites and regions of artifactual read enrichment (for some factors this overlap may be negligible because they do not bind to Sono-seq regions); on the magnitude of the Sono-seq effect; and on the strength of the ChIP itself (sufficiently strong determinations are not greatly affected). Conversely, if a ChIP-seq library has a strong Sono-seq component and peak calling is performed against an imperfectly matched “control” sample in which the Sono-seq effect is of significantly lower magnitude, false-positive peak calls will increase. Unfortunately, in practice such cases are difficult to detect. They are not flagged directly by current quality metrics and are best detected by analyses specific to each study and factor, including specific motif enrichment. especially when little is known about the expected true-positive rates. Similar reasoning applies if the noise source is something other than Sono-seq.

Uniform retrospective quality assessment is resource-intensive and will not be practically feasible because the number of ChIP-seq datasets is growing exponentially. Retrospective analysis also comes too late to influence the experiments themselves or to contribute to the review process. A reasonable path forward would be to incorporate routine data quality assessment into experimental analysis, review for publication, and submission to public repositories, as a matter of community practice. However, our results also strongly caution against the blind and arbitrary application of our metrics (or others) in the absence of experimental and biological context. The character of the metrics used here reflects contemporary technology and the quality scale has been calibrated based on factors and co-factors most studied to date. We have seen that it is possible for good datasets to receive low QC scores in certain special situations (*e.g.*, very few sites of occupancy in the genome). It is also possible for some poor or mediocre datasets to receive high QC scores. For example, this can happen as a side-product of strongly clustered backgrounds of the kind discussed above. Some examples of datasets in which this might be the case are shown in Figure S11. For factors that ChIP extremely well, even datasets that are substantially suboptimal score highly. For example, CTCF ChIP-seq datasets routinely identify 35,000–40,000 reproducible binding sites and have QC scores of 2; a dataset that identifies only 15,000 sites is suboptimal given that knowledge; yet it will still receive a positive QC score. For these reasons, the current quality metrics are best used in the context of what is known about the factor, the biological system, and the questions being asked.

Despite important nuances of interpretation, we suggest that using ChIP quality metrics and making the results readily accessible will facilitate better-informed data use by the wider community. An important adjunct to routine QC annotation would be the ability, in major public data repositories, to flag and explain the exceptional cases for which QC scores should not be taken at face value. Finally, quality metrics themselves will continue to improve as the field’s understanding of data structure, experimental artifacts, and the underlying biology all become more sophisticated. Provisions will be needed for incorporating such advances into routine dataset annotation while still achieving comparability through time.

## Supplementary Material

Supporting Information
