# Supplementary material for: Large-Scale Quality Analysis of Published ChIP-seq Data
Source: G3 (Bethesda). 2013 Dec 17;4(2):209–23. doi: 10.1534/g3.113.008680 (PMC3931556; doi:10.1534/g3.113.008680)
Supplement: Supporting Information [file supp_4_2_209__index.html]

Large-Scale Quality Analysis of Published ChIP-seq Data — Supporting Information 

# Large-Scale Quality Analysis of Published ChIP-seq Data

## Supporting Information for Marinov *et al.*, 2014

**Files in this Data Supplement:**

- Supporting Information - Figures S1-S11, Tables S1-S2, and References (PDF, 3 MB)
- Figure S1 - Examples of cross-correlation plots and QC score assignments for both ChIP- seq and control datasets. (PDF, 303 KB)
- Figure S2 - Distribution of the maximum SPP QC scores for studies in which only a single transcription factor was assayed. (PDF, 89 KB)
- Figure S3 - Sequencing depth distribution for ChIP-seq and IgG and Input control datasets. (PDF, 98 KB)
- Figure S4 - Distribution of library complexity values and sequencing depth for Input and IgG control datasets divided by QC scores. (PDF, 186 KB)
- Figure S5 - Relation between a well defined set of promoter-proximal and promoter-distal transcription factor binding sites and input datasets with minimal and significant read clustering. (PDF, 1 MB)
- Figure S6 - Distribution of signal around TSSs in control datasets. (PDF, 234 KB)
- Figure S7 - Distribution of dataset quality relative to year of publication. (PDF, 142 KB)
- Figure S8 - Distribution of dataset quality relative to the impact factor of the journal where an article was published. (PDF, 261 KB)
- Figure S9 - Distribution of the number of mapped reads and library complexity for data from the main two TF ChIP-seq production groups in ENCODE. (PDF, 183 KB)
- Figure S10 - Distribution of the discretized RSC QC scores for data from the main two TF ChIP-seq production groups in ENCODE. (PDF, 176 KB)
- Figure S11 - Examples of ChIP-seq datasets with high cross-correlation scores, which, however, seem to contain an unexplained source of read clustering other than specific immunoprecipitation enrichment of the targeted protein as measured by cross-correlation. (PDF, 390 KB)
- Table S1 - Dataset QC evaluation and mapping statistics. (PDF, 196 KB)
- Table S2 - Dataset QC evaluation and mapping statistics for MyoD and myogenin datasets. (PDF, 69 KB)
