# Supplementary material for: Large-Scale Quality Analysis of Published ChIP-seq Data
Source: G3 (Bethesda). 2013 Dec 17;4(2):209–23. doi: 10.1534/g3.113.008680 (PMC3931556; doi:10.1534/g3.113.008680)
Supplement: Supporting Information [file supp_g3.113.008680_FigureS1.pdf]

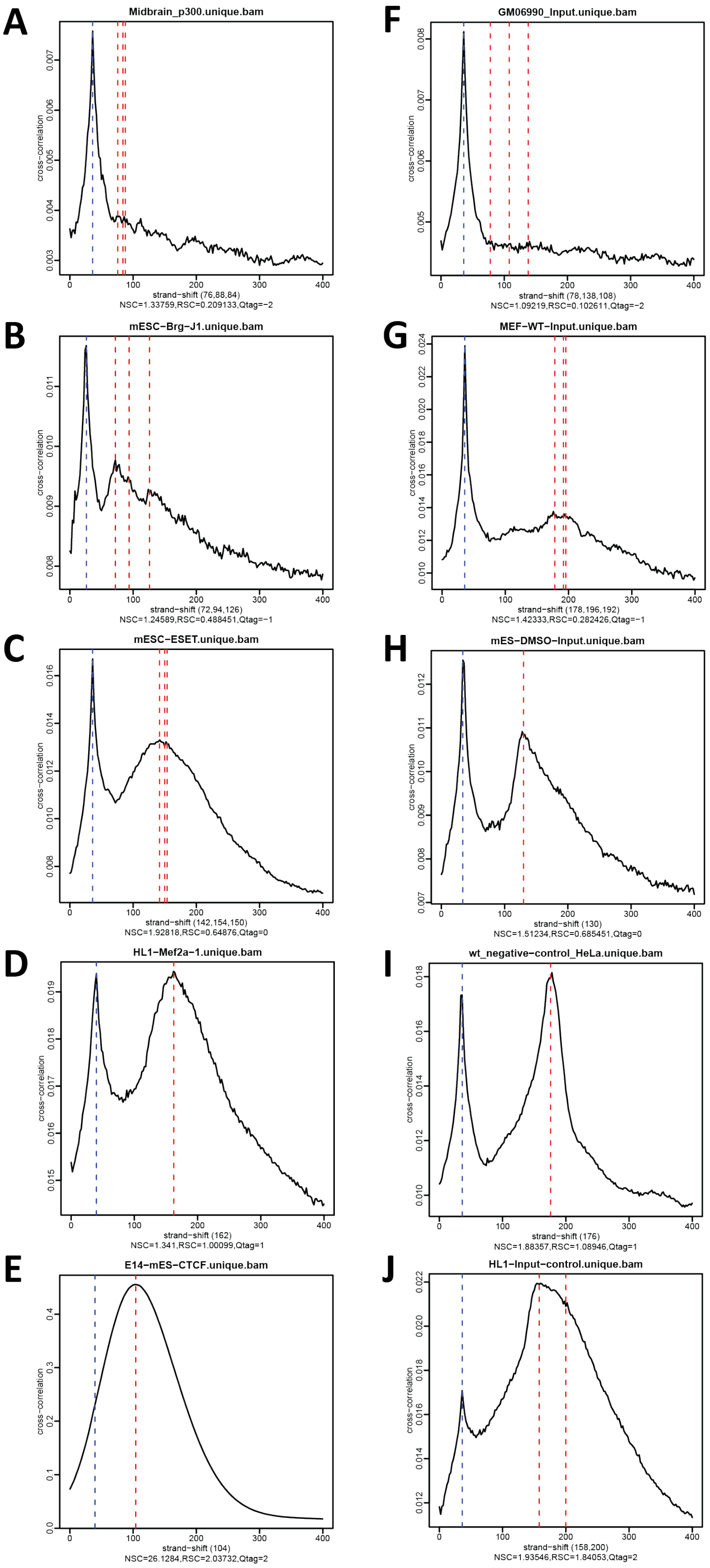
A
Midbrain_p300.unique.bam
cross−correlation
strand−shift (76,88,84)
NSC=1.33759,RSC=0.209133,Qtag=−2
F
GM06990_Input.unique.bam
strand−shift (78,138,108)
NSC=1.09219,RSC=0.102611,Qtag=−2
B
mESC−Brg−J1.unique.bam
strand−shift (72,94,126)
NSC=1.24589,RSC=0.488451,Qtag=−1
G
MEF−WT−Input.unique.bam
strand−shift (178,196,192)
NSC=1.42333,RSC=0.282426,Qtag=−1
C
mESC−ESET.unique.bam
strand−shift (142,154,150)
NSC=1.92818,RSC=0.64876,Qtag=0
H
mES−DMSO−Input.unique.bam
strand−shift (130)
NSC=1.51234,RSC=0.685451,Qtag=0
D
HL1−Mef2a−1.unique.bam
strand−shift (162)
NSC=1.341,RSC=1.00099,Qtag=1
I
wt_negative−control_HeLa.unique.bam
strand−shift (176)
NSC=1.88357,RSC=1.08946,Qtag=1
E
E14−mES−CTCF.unique.bam
strand−shift (104)
NSC=26.1284,RSC=2.03732,Qtag=2
J
HL1−Input−control.unique.bam
strand−shift (158,200)
NSC=1.93546,RSC=1.84053,Qtag=2

Figure S1 ***(preceding page)*****: Examples of cross-correlation plots and QC score assignments for both ChIP-seq and control datasets.** Successful ChIP-seq is expected to show a very high cross-correlation peak relative to the read length "phantom peak". Failed ChIP-seq experiments lack such a peak. Control libraries (sonicated inputs or IgG input) are also expected to lack this peak; the presence of a high cross-correlation peak is most likely due to a very strong Sono-seq effect (Auerbach et al. 2009). (A). Example of a ChIP-seq dataset with QC score of -2 (from Visel et al. 2009; Gotea et al. 2010; Blow et al. 2010). (B). Example of a ChIP-seq dataset with QC score of -1 (from Ho et al. 2009). (C). Example of a ChIP-seq dataset with QC score of 0 (from Yuan et al. 2009). (D). Example of a dataset with QC score of 1 (from He et al. 2011). (E). Example of a ChIP-seq dataset with QC score of 2 (from Handoko et al. 2011). (F). Example of a control dataset with QC score of -2 (from Lee et al. 2010). (G). Example of a control dataset with QC score of -1 (from GSE15844). (H). Example of a control dataset with QC score of 0 (from GSE23581). (I). Example of a dataset with QC score of 1 (from Vermeulen et al. 2010). (J). Example of a control dataset with QC score of 2 (from He et al. 2011).
