# Supplementary figures and images for: Large-Scale Quality Analysis of Published ChIP-seq Data
Source: G3 (Bethesda). 2013 Dec 17;4(2):209–23. doi: 10.1534/g3.113.008680 (PMC3931556; doi:10.1534/g3.113.008680)

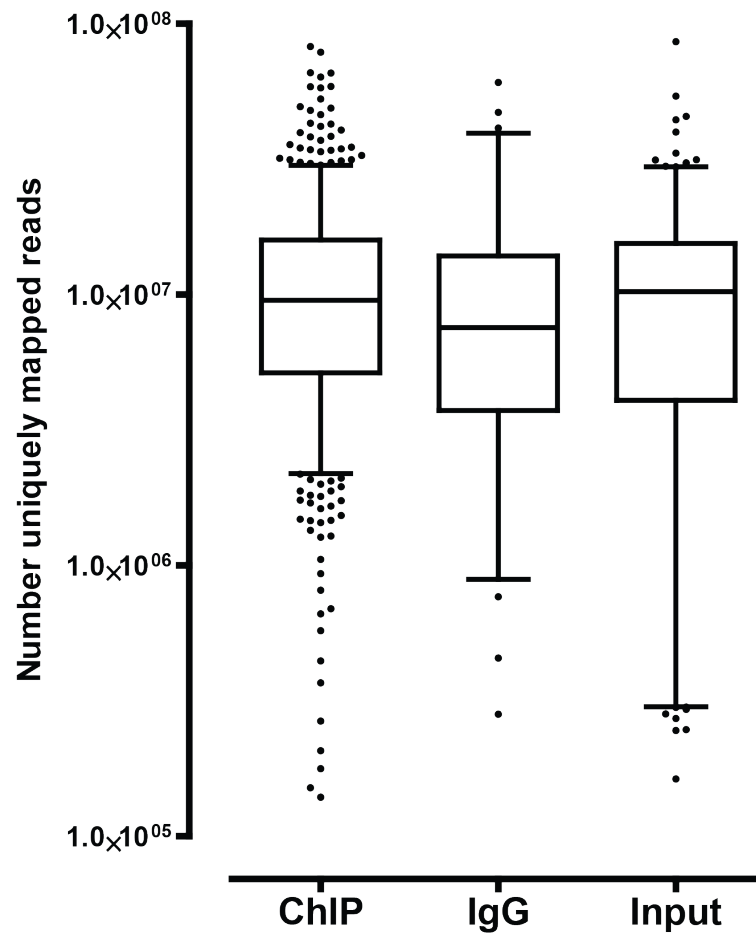

Figure S3: Sequencing depth distribution for ChIP-seq and IgG and Input control datasets.

Supplement: Supporting Information [file supp_g3.113.008680_FigureS3.pdf]

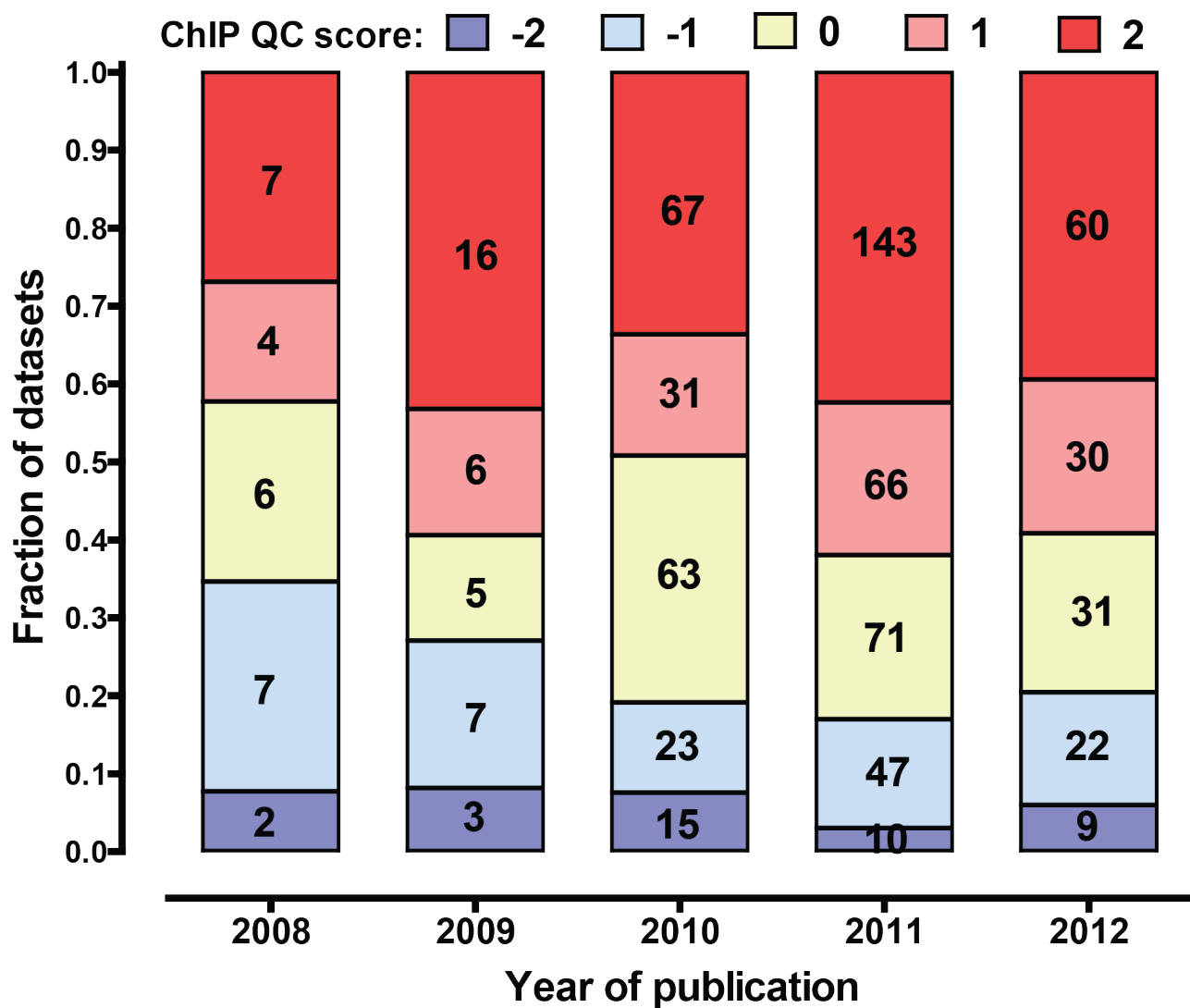

Figure S7: Distribution of dataset quality relative to year of publication.

Supplement: Supporting Information [file supp_g3.113.008680_FigureS7.pdf]
