# Supplementary material for: Large-Scale Quality Analysis of Published ChIP-seq Data
Source: G3 (Bethesda). 2013 Dec 17;4(2):209–23. doi: 10.1534/g3.113.008680 (PMC3931556; doi:10.1534/g3.113.008680)
Supplement: Supporting Information [file supp_g3.113.008680_FigureS4.pdf]

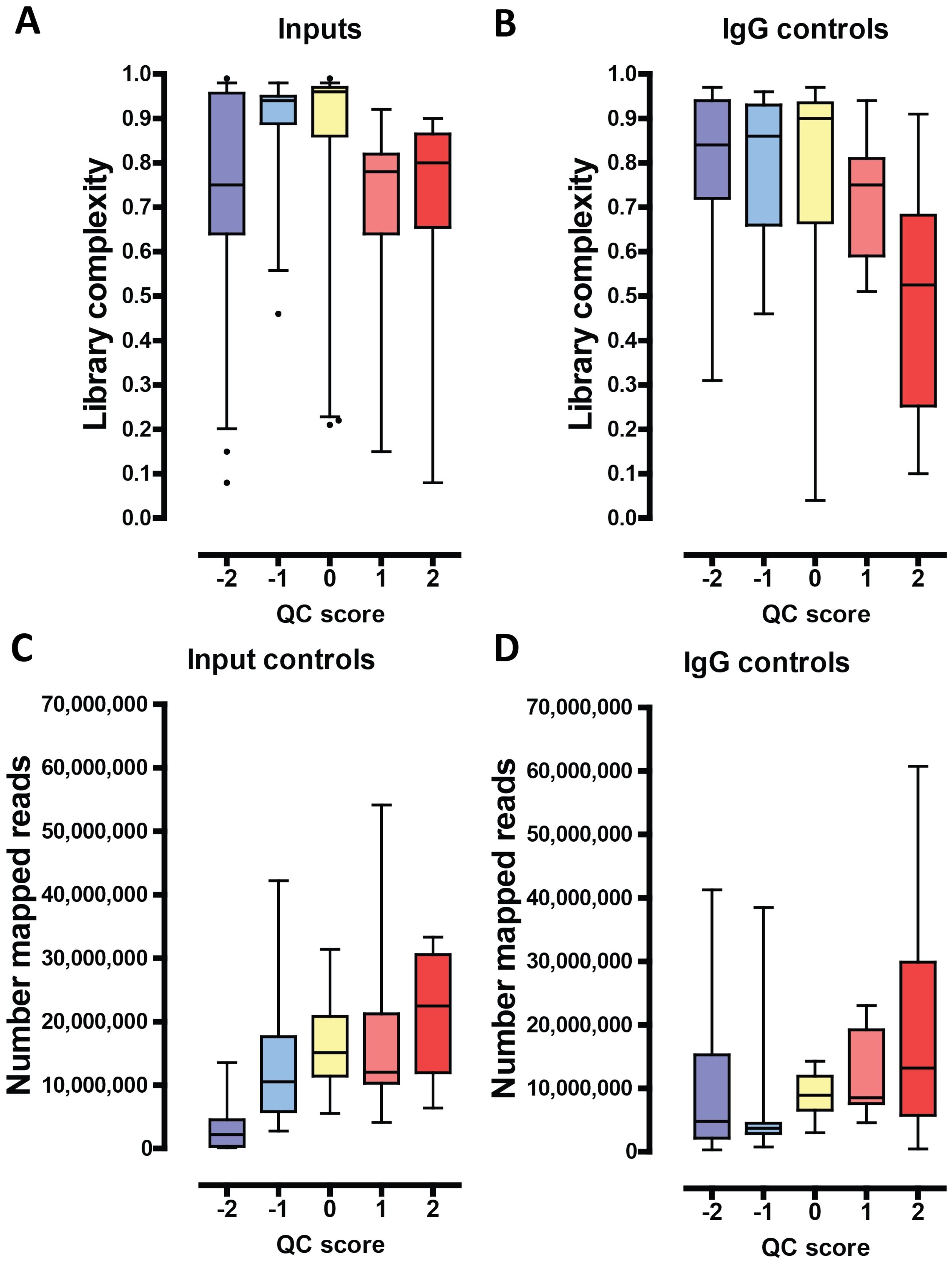


**Figure S4: Distribution of library complexity values and sequencing depth for Input and IgG control datasets divided by QC scores.** (A,B) Library complexity. (C,D) Sequencing depth.
