# Supplementary material for: Large-Scale Quality Analysis of Published ChIP-seq Data
Source: G3 (Bethesda). 2013 Dec 17;4(2):209–23. doi: 10.1534/g3.113.008680 (PMC3931556; doi:10.1534/g3.113.008680)
Supplement: Supporting Information [file supp_g3.113.008680_FigureS5.pdf]

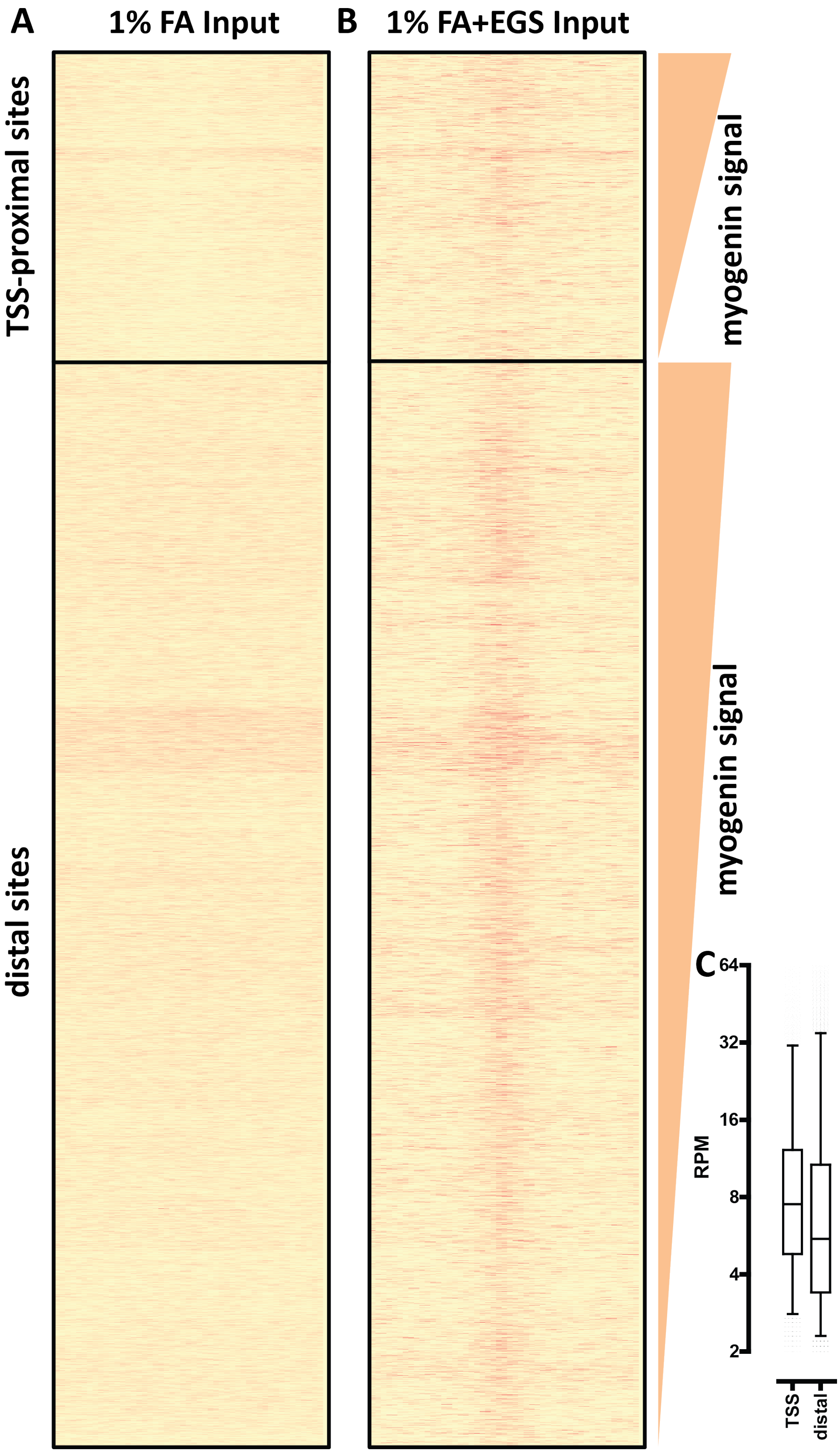
A
1% FA Input
B
1% FA+EGS Input
TSS-proximal sites
distal sites
myogenin signal
myogenin signal
C
RPM
64
32
16
8
4
2
TSS
distal

Figure S5 ***(preceding page)*****: Relation between a well defined set of promoter-proximal and promoter-distal transcription factor binding sites and input datasets with minimal and significant read clustering.** The high-quality C2C12 myogenin dataset shown in Figure 4 was used, ERANGE3.2 binding sites were separated into promoter promoter-proximal (sites for which the peak position, defined by the peak caller was within 1kb of a TSS present in the ENSEMBL63 annotation of the mm9 version of the mouse genome) and promoter-distal (sites for which the peak position was more than 1kb away from TSSs) groups, each group was ranked by decreasing myogenin signal and the distribution of input signal was plotted for the 1kb region around the peak position. (A) A C2C12 input dataset generated from cells fixed with the usually used 1% concentration of formaldehyde (FA) for 15 minutes, and showing little read clustering genome-wide (QC score of -1). (B) A C2C12 input dataset generated from cells fixed with a combination of 1% formaldehyde (for 10 minutes) and subsequent additional fixation with the long-arm crosslinker ethylene glycolbis(succinimidylsuccinate) (EGS) (Abdella et al. 1979) in order to enhance crosslinking between proteins and capture the interactions of factors more loosely associated with chromatin (Zeng et al. 2006). There are reason to expect that such more aggressive crosslinking conditions will results in a stronger Sono-seq effect and indeed this dataset exhibits significant amount of read clustering (QC score of 2). The 1%FA+EGS input signal around myogenin binding sites is considerably higher than the 1% FA input signal. Notably, the 1%FA+EGS signal signal is stronger for promoter-distal sites than it is for promoter-proximal sites even though promoter-proximal sites are generally stronger (C).
