# Supplementary material for: Large-Scale Quality Analysis of Published ChIP-seq Data
Source: G3 (Bethesda). 2013 Dec 17;4(2):209–23. doi: 10.1534/g3.113.008680 (PMC3931556; doi:10.1534/g3.113.008680)
Supplement: Supporting Information [file supp_g3.113.008680_FigureS6.pdf]

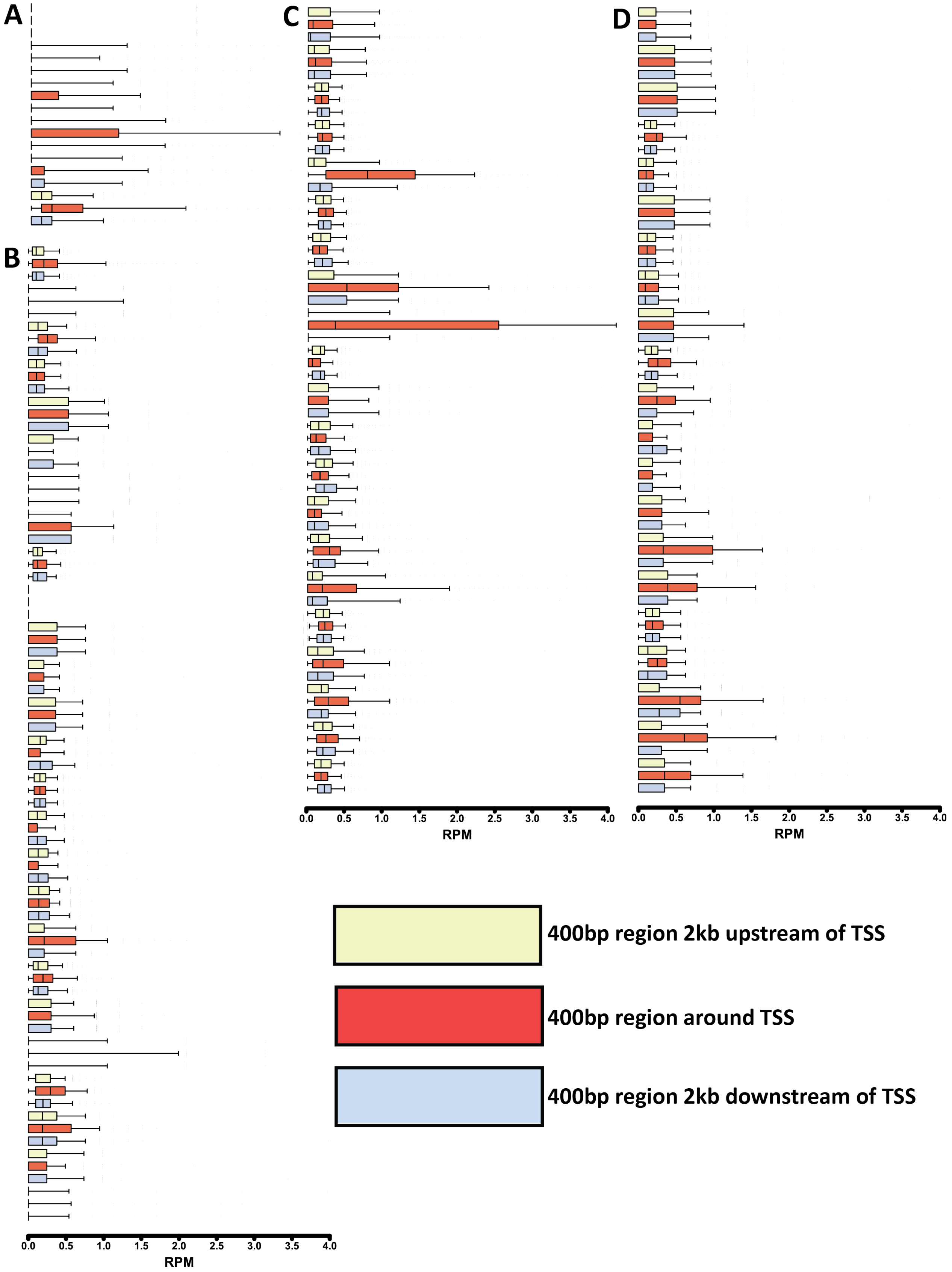
A
B
C
D
0.0
0.5
1.0
1.5
2.0
2.5
3.0
3.5
4.0
RPM
400bp region 2kb upstream of TSS
400bp region around TSS
400bp region 2kb downstream of TSS

Figure S6 *(preceding page)*: Distribution of signal around TSSs in control datasets. Each group of three blue, red and yellow boxplots represents to one dataset, with blue corresponding to a region 2kb upstream of TSSs, red to the region immediately surrounding the TSS, and yellow to a region 2kb downstream of TSS. Datasets in which signal over TSSs is considerably higher than the signal over flanking regions imply a possible "Sono-seq" overrepresentation effect; this, however, is not evident (at least over TSSs) in all highly clustered datasets. (A) Human control datasets with a QC score of +2. (B) Human control datasets with a QC score of -2. (C) Mouse control datasets with a QC score of +2. (D) Mouse control datasets with a QC score of -2.
