# Supplementary material for: Large-Scale Quality Analysis of Published ChIP-seq Data
Source: G3 (Bethesda). 2013 Dec 17;4(2):209–23. doi: 10.1534/g3.113.008680 (PMC3931556; doi:10.1534/g3.113.008680)
Supplement: Supporting Information [file supp_g3.113.008680_FigureS8.pdf]

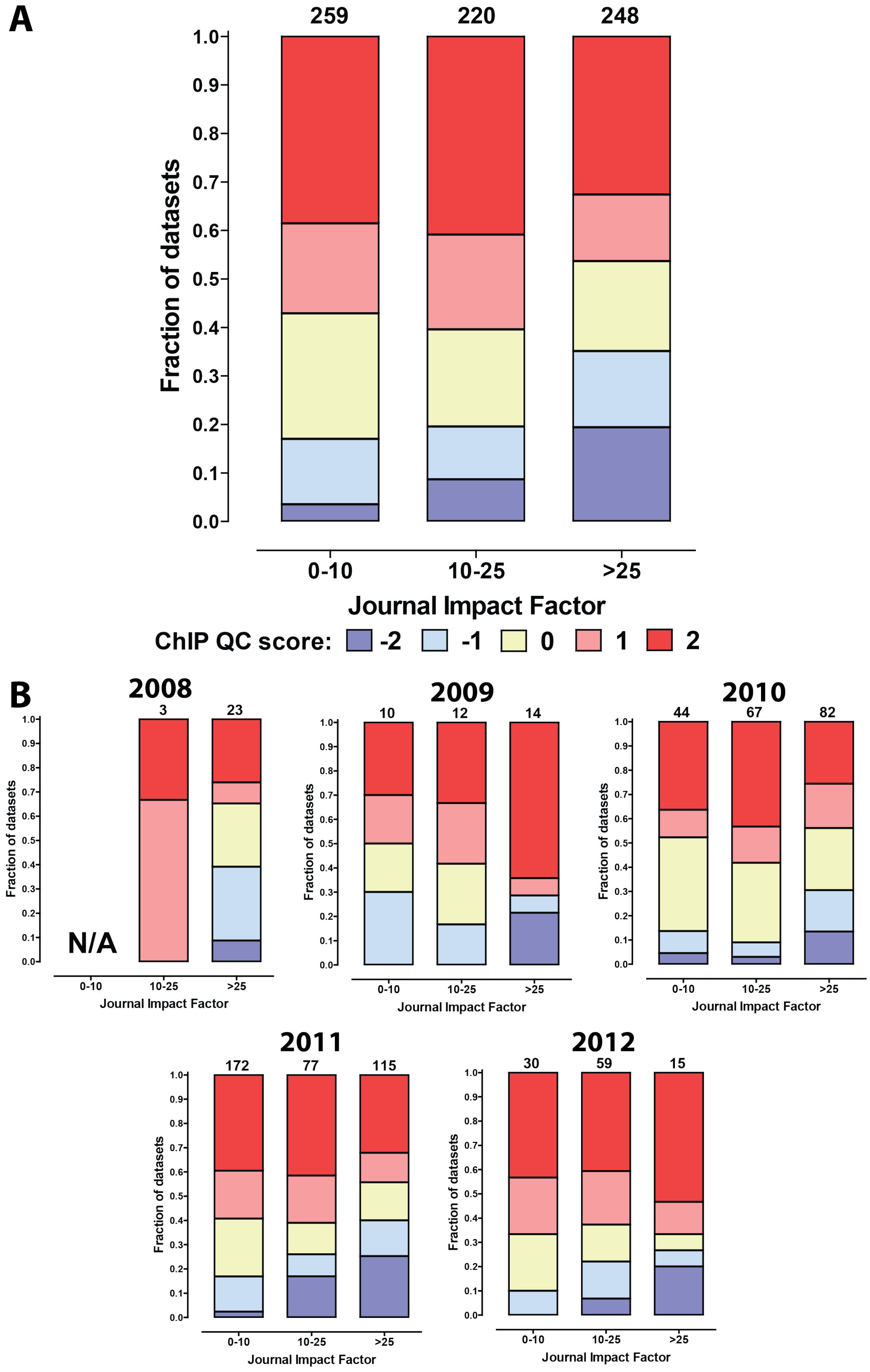

Figure S8: Distribution of dataset quality relative to the impact factor of the journal where an article was published. Shown are the 2011 Thompson-Reuters impact factor scores for the journals in which ChIP-seq datasets were published in. (A) All datasets. (B). Breakdown by year of publication.
