# Supplementary material for: Large-Scale Quality Analysis of Published ChIP-seq Data
Source: G3 (Bethesda). 2013 Dec 17;4(2):209–23. doi: 10.1534/g3.113.008680 (PMC3931556; doi:10.1534/g3.113.008680)
Supplement: Supporting Information [file supp_g3.113.008680_FigureS9.pdf]

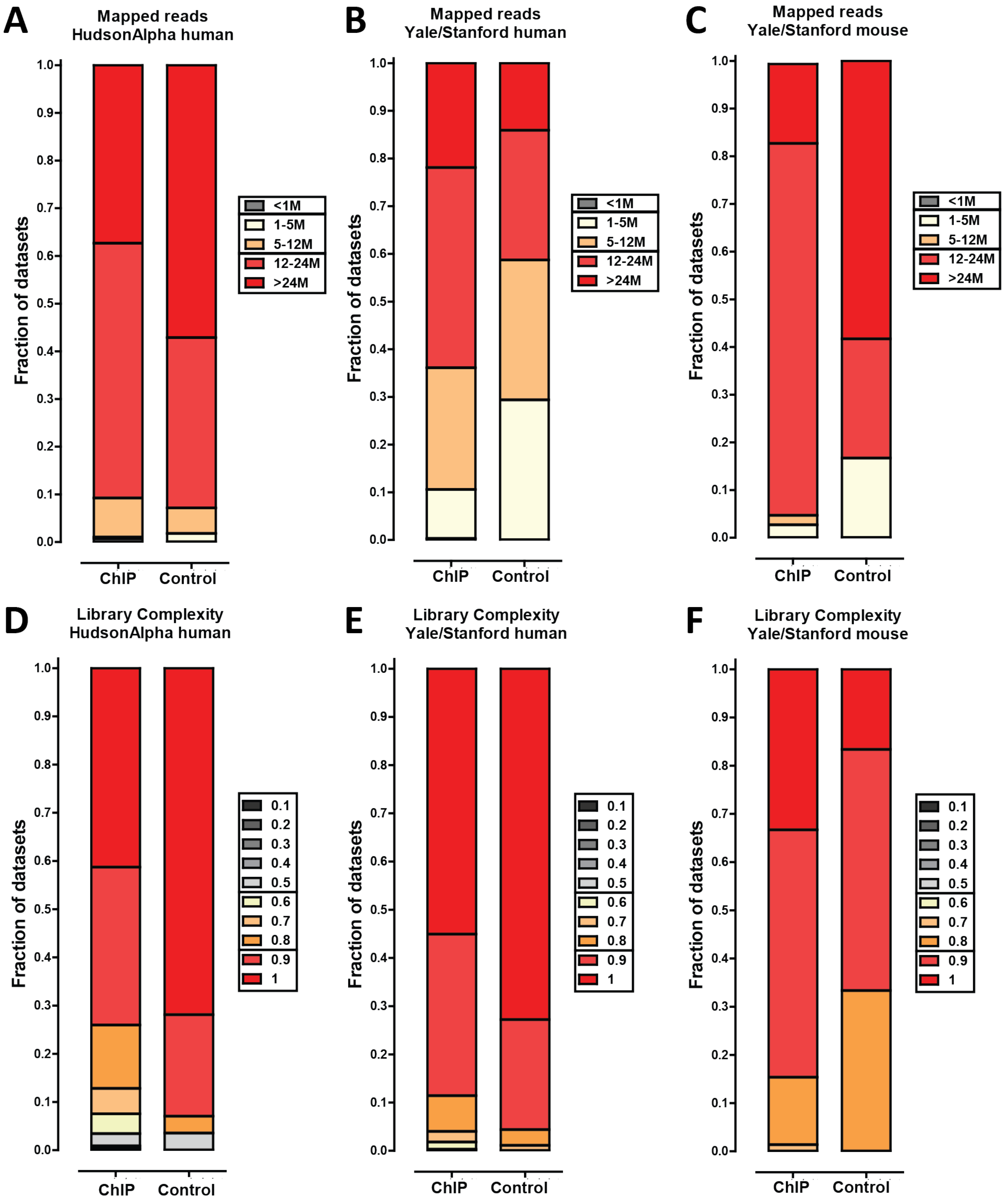

Figure S9: Distribution of the number of mapped reads and library complexity for data from the main two TF ChIP-seq production groups in ENCODE. (A,B,C) Number mapped reads. (D,E,F). Library complexity. Note that the same filters on the dataset inclusions that were used on publicly available data (see Methods section) were also applied to ENCODE datasets.
