# Supplementary material for: Large-Scale Quality Analysis of Published ChIP-seq Data
Source: G3 (Bethesda). 2013 Dec 17;4(2):209–23. doi: 10.1534/g3.113.008680 (PMC3931556; doi:10.1534/g3.113.008680)
Supplement: Supporting Information [file supp_g3.113.008680_FigureS10.pdf]

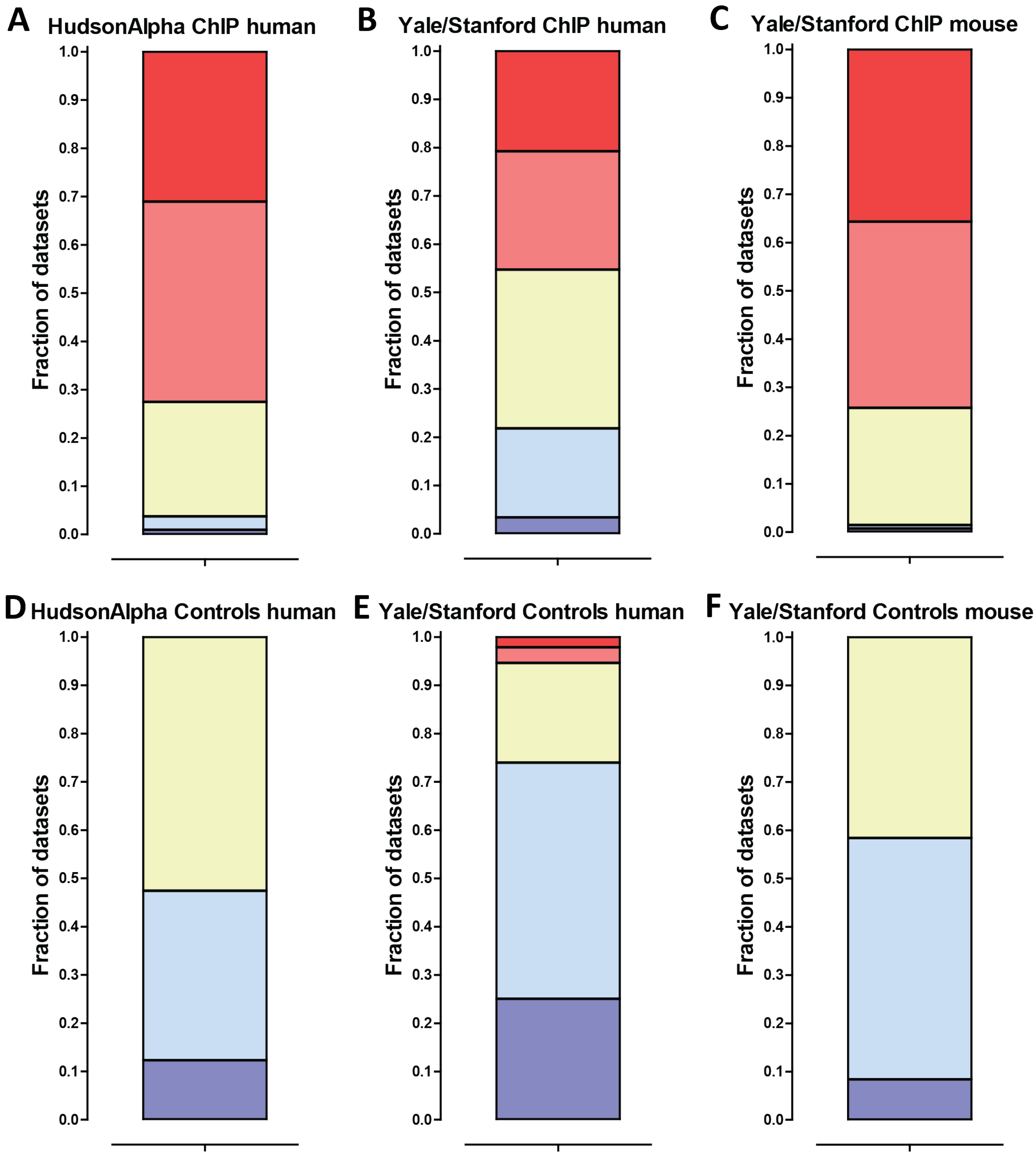

Figure S10: Distribution of the discretized RSC QC scores for data from the main two TF ChIP-seq production groups in ENCODE. (A,B,C) Transcription factor ChIP-seq data. (D,E,F). Control datasets (Input and IgG).
