# Supplementary material for: Large-Scale Quality Analysis of Published ChIP-seq Data
Source: G3 (Bethesda). 2013 Dec 17;4(2):209–23. doi: 10.1534/g3.113.008680 (PMC3931556; doi:10.1534/g3.113.008680)
Supplement: Supporting Information [file supp_g3.113.008680_FigureS11.pdf]

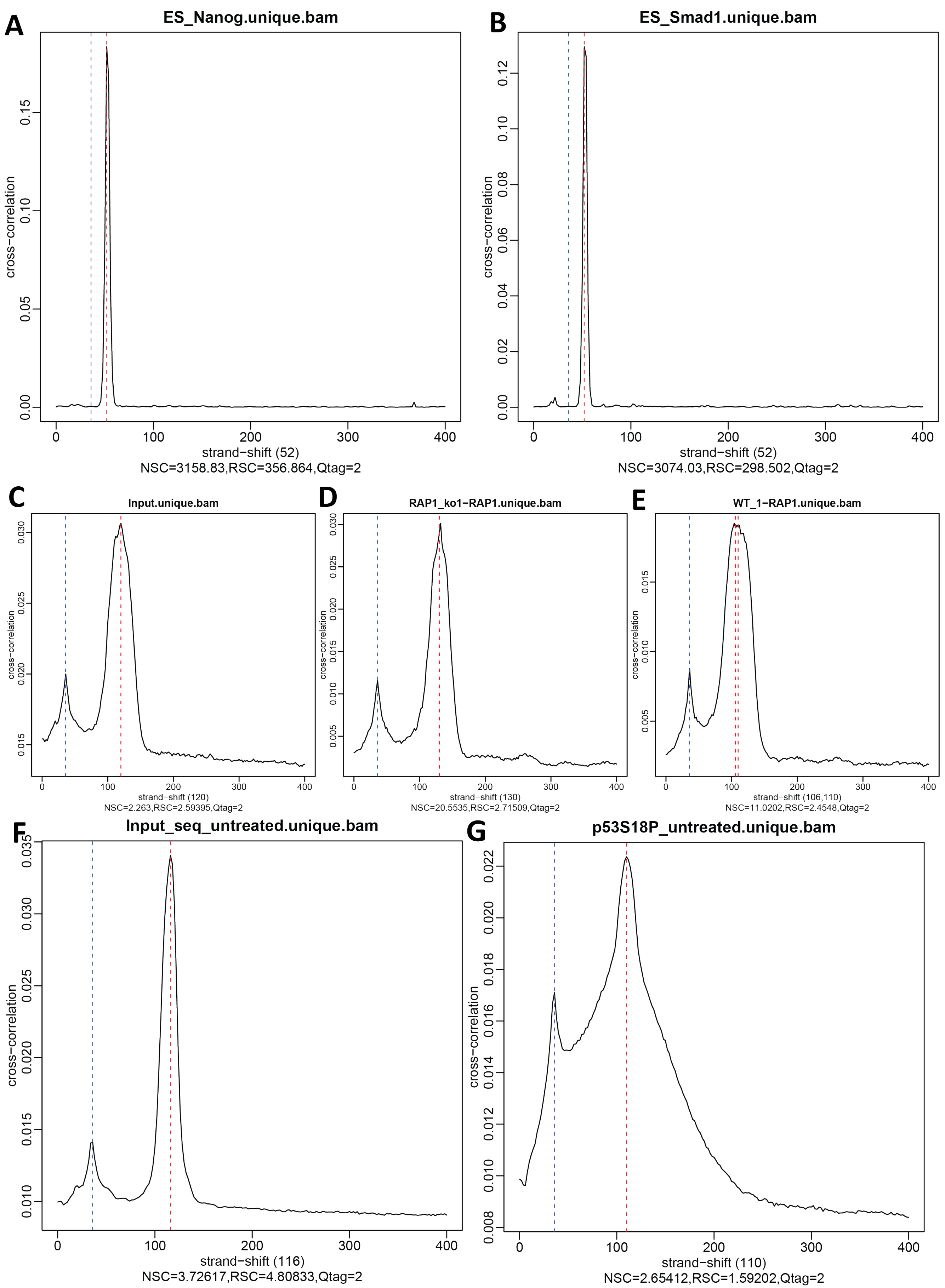
A
ES_Nanog.unique.bam
cross−correlation
strand−shift (52)
NSC=3158.83,RSC=356.864,Qtag=2
B
ES_Smad1.unique.bam
cross−correlation
strand−shift (52)
NSC=3074.03,RSC=298.502,Qtag=2
C
Input.unique.bam
cross−correlation
strand−shift (120)
NSC=2.263,RSC=2.59395,Qtag=2
D
RAP1_ko1−RAP1.unique.bam
cross−correlation
strand−shift (130)
NSC=20.5535,RSC=2.71509,Qtag=2
E
WT_1−RAP1.unique.bam
cross−correlation
strand−shift (106,110)
NSC=11.0202,RSC=2.4548,Qtag=2
F
Input_seq_untreated.unique.bam
cross−correlation
strand−shift (116)
NSC=3.72617,RSC=4.80833,Qtag=2
G
p53S18P_untreated.unique.bam
cross−correlation
strand−shift (110)
NSC=2.65412,RSC=1.59202,Qtag=2

**Figure S11** ***(preceding page)*****: Examples of ChIP-seq datasets with high cross-correlation scores, which, however, seem to contain an unexplained source of read clustering other than specific immunoprecipitation enrichment of the targeted protein as measured by cross-correlation**. (A,B) Cross-correlation plots for Nanog and Smad1 ChIP-seq in mESC cells from Chen et al. 2008. Each dataset receives extremely high RSC scores (356.86 and 298.50, respectively), but the cross-correlation profiles are very atypical, displaying an strikingly narrow peak and a very small phantom peak. (C,D,E). RAP1 ChIP-seq in wild type (WT) and RAP1 knock out (KO) cells as well as a corresponding input sample from Martinez et al. 2010. Here both the input and the KO sample ChIP (whcih should exhibit no immunoprecipitation enrichment) have a very high enrichment peak and high RSC scores. Thus the observed enrichment in the WT RAP1 ChIP-seq sample likely contains a significant contribution from a source different from actual RAP1 binding events. (F,G) A similar situation in a ChIP-seq dataset targeting p53 phosphorylated at Ser18 (p53-pS18) (Li et al. 2012). ).
