# Supplementary material for: Large-Scale Quality Analysis of Published ChIP-seq Data
Source: G3 (Bethesda). 2013 Dec 17;4(2):209–23. doi: 10.1534/g3.113.008680 (PMC3931556; doi:10.1534/g3.113.008680)
Supplement: Supporting Information [file supp_g3.113.008680_TableS1.pdf]

Table S1: Dataset QC evaluation and mapping statistics. A direct link to the GEO entry is provided in the. "Source" field

Source	Species	Library	Complexity	NSC	RSC	QC	Ave. Read Length	Max. Read Length	Min. Read Length	Mapped reads	Type	Should exhibit read clustering
Marson et al. 2008	mouse	Nanog-mES-rep1	0.94	4.37	1.67	2	26	26	26	4,305,381	ChIP	yes
Marson et al. 2008	mouse	Nanog-mES-rep2	0.94	4.32	1.67	2	26	26	26	4,396,374	ChIP	yes
Marson et al. 2008	mouse	oct4-mES-rep1	0.95	6.54	0.34	-1	26	26	26	4,341,147	ChIP	yes
Marson et al. 2008	mouse	sox2-mES-rep1	0.96	4.03	1.21	1	26	26	26	4,033,101	ChIP	yes
Marson et al. 2008	mouse	sox2-mES-rep2	0.97	4.07	1.19	1	26	26	26	3,287,130	ChIP	yes
Marson et al. 2008	mouse	suz12-mES-repl	0.97	1.63	0.15	-2	26	26	26	3,624,473	ChIP	yes
Marson et al. 2008	mouse	suz12-rep2-1	0.98	1.24	0.41	-1	26	26	26	207,308	ChIP	yes
Marson et al. 2008	mouse	Tcf3-mES-rep1	0.96	3	0.72	0	26	26	26	5,247,274	ChIP	yes
Marson et al. 2008	mouse	Tcf3-mES-rep2	0.96	2.96	0.66	0	26	26	26	5,388,916	ChIP	yes
Marson et al. 2008	mouse	WCE-mES-rep1	0.94	1.37	0.2	-2	26	26	26	1,507,157	Input	no
Marson et al. 2008	mouse	WCE-mES-rep2	0.9	1.35	0.21	-2	26	26	26	3,770,502	Input	no
Chen et al. 2008	mouse	ES-c-Myc	0.86	1.51	0.48	-1	26	26	26	11,714,595	ChIP	yes
Chen et al. 2008	mouse	ES-E2f1	0.84	1.43	0.92	0	26	26	26	13,374,901	ChIP	yes
Chen et al. 2008	mouse	ES-Esrrb	0.88	4.5	1.69	2	26	26	26	7,982,162	ChIP	yes
Chen et al. 2008	mouse	ES-GFP	0.82	1.26	0.15	-2	26	26	26	7,520,858	IgG	no
Chen et al. 2008	mouse	ES-Klf4	0.41	1.96	0.62	0	36	36	36	368,908	ChIP	yes
Chen et al. 2008	mouse	ES-Nanog	0.81	3158	356	2	26	26	26	9,166,834	ChIP	yes
Chen et al. 2008	mouse	ES-n-Myc	0.79	1.74	0.41	-1	26	26	26	10,099,160	ChIP	yes
Chen et al. 2008	mouse	ES-Oct4	0.59	1.61	0.46	-1	36	36	36	139,512	ChIP	yes
Chen et al. 2008	mouse	ES-p300	0.77	1.26	0.23	-2	26	26	26	9,396,456	ChIP	yes
Chen et al. 2008	mouse	ES-Smad1	0.96	3074	298	2	26	26	26	9,681,328	ChIP	yes
Chen et al. 2008	mouse	ES-Sox2	0.86	1.94	0.62	0	26	26	26	12,489,175	ChIP	yes
Chen et al. 2008	mouse	ES-STAT3	0.77	1.68	0.38	-1	26	26	26	8,384,452	ChIP	yes
Chen et al. 2008	mouse	ES-Suz12	0.87	1.21	0.27	-1	26	26	26	12,378,715	ChIP	yes
Chen et al. 2008	mouse	ES-Tcfcp2I1	0.81	13.53	2.42	2	26	26	26	8,800,970	ChIP	yes
Chen et al. 2008	mouse	ES-Zfx	0.92	1.9	0.71	0	31	31	31	9,543,774	ChIP	yes
Kwon et al. 2009	mouse	GIgG-post-IL21-in-B-cells	0.84	1.4	0.47	-1	25	25	25	2,915,090	IgG	no
Kwon et al. 2009	mouse	GIgG-post-IL6	0.88	1.49	0.36	-1	25	25	25	2,129,448	IgG	no
Kwon et al. 2009	mouse	IgG-post-IL21-dup	0.89	1.26	0.34	-1	25	25	25	4,286,349	IgG	no
Kwon et al. 2009	mouse	IgG-post-IL21-in-B-cells	0.91	1.33	0.52	0	25	25	25	2,993,063	IgG	no
Kwon et al. 2009	mouse	IgG-post-IL21-in-WT-quar	0.74	1.89	0.7	0	25	25	25	7,228,784	IgG	no
Kwon et al. 2009	mouse	IgG-post-IL21-ter	0.92	1.35	0.11	-2	25	25	25	3,080,974	IgG	no
Kwon et al. 2009	mouse	IgG-post-IL2-dup	0.86	1.31	0.4	-1	25	25	25	3,406,531	IgG	no
Kwon et al. 2009	mouse	IgG-post-IL6	0.84	1.31	0.35	-1	25	25	25	4,247,181	IgG	no
Kwon et al. 2009	mouse	IRF4-post-IL21-dup-seq-1	0.86	2.08	0.89	0	25	25	25	3,295,774	ChIP	yes
Kwon et al. 2009	mouse	IRF4-post-IL21-dup-seq-2	0.66	2.21	0.92	0	25	25	25	5,496,827	ChIP	yes
Kwon et al. 2009	mouse	IRF4-post-IL21-in-IRF4-KO-mice	0.94	1.9	0.66	0	25	25	25	3,613,839	ChIP	no
Kwon et al. 2009	mouse	IRF4-post-IL21-seq-1	0.9	1.88	0.72	0	25	25	25	3,560,575	ChIP	yes
Kwon et al. 2009	mouse	IRF4-post-IL21-seq-2	0.93	2.14	0.73	0	25	25	25	1,273,441	ChIP	yes
Kwon et al. 2009	mouse	IRF4-pre-IL21-dup-seq-1	0.89	1.76	0.7	0	25	25	25	3,996,341	ChIP	yes
Kwon et al. 2009	mouse	IRF4-pre-IL21-dup-seq-2	0.9	1.83	0.72	0	25	25	25	3,308,064	ChIP	yes
Kwon et al. 2009	mouse	IRF4-pre-IL21-in-IRF4-KO-mice	0.81	1.5	0.42	-1	25	25	25	5,741,089	ChIP	no
Kwon et al. 2009	mouse	IRF4-pre-IL21-seq-1	0.89	2.19	0.9	0	25	25	25	2,882,656	ChIP	yes
Kwon et al. 2009	mouse	IRF4-pre-IL21-seq-2	0.73	2.44	0.84	0	25	25	25	4,304,206	ChIP	yes
Kwon et al. 2009	mouse	STAT3-post-IL21-dup-seq-1	0.93	1.47	0.44	-1	25	25	25	266,685	ChIP	yes
Kwon et al. 2009	mouse	STAT3-post-IL21-dup-seq-2	0.89	1.47	0.46	-1	25	25	25	1,818,900	ChIP	yes
Kwon et al. 2009	mouse	STAT3-post-IL21-in-IRF4-KO-mice	0.79	1.42	0.28	-1	25	25	25	5,334,084	ChIP	unknown
Kwon et al. 2009	mouse	STAT3-post-IL21-in-IRF4-KO-mice-second-exp	0.81	1.52	0.52	0	25	25	25	5,744,414	ChIP	unknown
Kwon et al. 2009	mouse	STAT3-post-IL21-in-IRF4-KO-mice-third-exp	0.82	1.5	0.48	-1	25	25	25	4,041,874	ChIP	unknown
Kwon et al. 2009	mouse	STAT3-post-IL21-in-WT-cinq	0.77	1.87	0.58	0	25	25	25	5,281,605	ChIP	yes
Kwon et al. 2009	mouse	STAT3-post-IL21-in-WT-quar	0.76	1.88	0.63	0	25	25	25	5,450,390	ChIP	yes
Kwon et al. 2009	mouse	STAT3-post-IL21-in-WT-ter	0.85	1.46	0.43	-1	25	25	25	3,416,726	ChIP	yes
Kwon et al. 2009	mouse	STAT3-post-IL21-seq-1	0.88	2.75	0.86	0	25	25	25	3,446,457	ChIP	yes
Kwon et al. 2009	mouse	STAT3-post-IL21-seq-2	0.84	2.89	0.81	0	25	25	25	3,340,925	ChIP	yes
Kwon et al. 2009	mouse	STAT3-pre-IL21-dup-seq-1	0.82	1.37	0.41	-1	25	25	25	3,736,863	ChIP	unknown
Kwon et al. 2009	mouse	STAT3-pre-IL21-dup-seq-2	0.86	1.42	0.4	-1	25	25	25	2,709,584	ChIP	unknown
Kwon et al. 2009	mouse	STAT3-pre-IL21-in-IRF4-KO-mice-first-exp	0.85	1.56	0.45	-1	25	25	25	3,709,343	ChIP	unknown
Kwon et al. 2009	mouse	STAT3-pre-IL21-in-IRF4-KO-mice-second-exp	0.79	1.83	0.71	0	25	25	25	6,924,787	ChIP	unknown
Kwon et al. 2009	mouse	STAT3-pre-IL21-in-WT-4th-experiment	0.82	1.51	0.37	-1	25	25	25	4,257,111	ChIP	unknown
Kwon et al. 2009	mouse	STAT3-pre-IL21-in-WT-ter	0.81	1.41	0.14	-2	25	25	25	3,395,506	ChIP	unknown
Kwon et al. 2009	mouse	STAT3-pre-IL21-seq-1	0.78	3.1	0.76	0	25	25	25	3,700,560	ChIP	unknown
Kwon et al. 2009	mouse	STAT3-pre-IL21-seq-2	0.76	3.1	0.67	0	25	25	25	3,667,278	ChIP	unknown
Hollenhorst et al. 2009	human	Jurkat-CBP-1	0.86	2.22	1.38	1	36	36	36	9,275,556	ChIP	yes

Continued on next page

Table S1 – *Continued from previous page*

Source	Species	Library	Complexity	NSC	RSC	QC	Ave. Read Length	Max. Read Length	Min. Read Length	Mapped reads	Type	Should exhibit read clustering
Hollenhorst et al. 2009	human	Jurkat-ETS1-1	0.85	2.41	2.58	2	26	26	26	7,562,377	ChIP	yes
Hollenhorst et al. 2009	human	Jurkat-Input-1	0.93	1.16	0.15	-2	26	26	26	15,389,799	Input	no
Hollenhorst et al. 2009	human	Jurkat-RUNX-1	0.63	2.55	0.61	0	36	36	36	10,337,694	ChIP	yes
Han et al. 2010	mouse	mESC-Input	0.96	1.17	0.18	-2	37	37	37	9,567,449	ChIP	no
Han et al. 2010	mouse	mESC-Tbx3	0.92	1.46	0.29	-1	35	35	35	7,526,549	ChIP	yes
Yu et al. 2009	mouse	MEL86-GATA1	0.94	1.34	0.28	-1	33.87	36	28	5,866,520	ChIP	yes
De et al. 2009	mouse	Macrophages-JMJD3	0.95	1.32	0.36	-1	36	36	36	8,731,417	ChIP	yes
Yuan et al. 2009	mouse	mESC-ESET	0.92	1.93	0.65	0	36	36	36	11,607,868	ChIP	yes
Bilodeau et al. 2009	mouse	mESC-SetDB1-rep-1	0.92	1.68	0.54	0	36	36	36	3,620,404	ChIP	yes
Bilodeau et al. 2009	mouse	mESC-SetDB1-rep-2	0.93	1.64	0.42	-1	36	36	36	3,301,043	ChIP	yes
Bilodeau et al. 2009	mouse	mESC-SetDB1-rep-3	0.92	1.65	0.51	0	36	36	36	4,251,421	ChIP	yes
Bilodeau et al. 2009	mouse	mESC-WCE-mES-rep-1	0.94	1.38	0.31	-1	36	36	36	3,966,359	Input	no
Lister et al. 2009	human	hESC-NANOG-1a	0.65	18.86	4.47	2	36	36	36	3,701,686	ChIP	yes
Lister et al. 2009	human	hESC-NANOG-1b	0.61	17.44	4.43	2	36	36	36	4,523,040	ChIP	yes
Lister et al. 2009	human	hESC-SOX2-1a	0.82	9.01	4.94	2	36	36	36	4,591,769	ChIP	yes
Lister et al. 2009	human	hESC-KLF4-1a	0.32	46.06	24.39	2	36	36	36	810,796	ChIP	yes
Lister et al. 2009	human	hESC-MYC-1a	0.58	4.15	2.02	2	36	36	36	2,391,782	ChIP	yes
Lister et al. 2009	human	hESC-Oct4-1a	0.98	2.46	1.04	1	36	36	36	574,662	ChIP	yes
Lister et al. 2009	human	hESC-Oct4-2a	0.98	4.37	1.81	2	36	36	36	151,346	ChIP	yes
Lister et al. 2009	human	hESC-P300-1a	0.57	7.51	2.52	2	36	36	36	3,490,165	ChIP	yes
Lister et al. 2009	human	hESC-TAFII-1a	0.64	2.93	1.96	2	36	36	36	4,031,316	ChIP	yes
Lister et al. 2009	human	hESC-TAFII-1b	0.67	2.9	1.72	2	36	36	36	3,507,401	ChIP	yes
Nishiyama et al. 2009	mouse	mESC-Cdx2	0.94	1.14	0.32	-1	36	36	36	7,347,351	ChIP	yes
Cheng et al. 2009	mouse	G1E-ER4-GATA1	0.96	1.75	1.2	1	36	36	36	24,281,091	ChIP	yes
Cheng et al. 2009	mouse	G1E-ER4-Input	0.97	1.28	0.58	0	36	36	36	15,990,494	Input	no
Wilson et al. 2009	mouse	HPC-7-Scl-1	0.95	1.86	0.88	0	45	45	45	5,563,933	ChIP	yes
Wilson et al. 2009	mouse	HPC-7-Scl-2	0.96	1.83	0.47	-1	36	36	36	3,637,766	ChIP	yes
Robertson et al. 2007	human	HeLaS3-IFNgamma-1-STAT1	0.86	4.41	1.1	1	27	27	27	693,473	ChIP	yes
Robertson et al. 2007	human	HeLaS3-IFNgamma-2-STAT1	0.85	3.67	1.04	1	27	27	27	663,874	ChIP	yes
Robertson et al. 2007	human	HeLaS3-IFNgamma-3-STAT1	0.94	4.07	1.58	2	36	36	36	3,079,284	ChIP	yes
Robertson et al. 2007	human	HeLaS3-IFNgamma-4-STAT1	0.94	3.97	1.59	2	27	27	27	2,176,985	ChIP	yes
Robertson et al. 2007	human	HeLaS3-IFNgamma-5-STAT1	0.95	4	1.96	2	27	27	27	2,808,038	ChIP	yes
Robertson et al. 2007	human	HeLaS3-IFNgamma-6-STAT1	0.95	4.17	1.95	2	27	27	27	2,718,185	ChIP	yes
Robertson et al. 2007	human	HeLaS3-unstimulated-1-STAT1	0.86	1.98	0.33	-1	27	27	27	478,619	ChIP	unknown
Robertson et al. 2007	human	HeLaS3-unstimulated-2-STAT1	0.84	2.6	0.34	-1	27	27	27	500,638	ChIP	unknown
Robertson et al. 2007	human	HeLaS3-unstimulated-3-STAT1	0.82	2.2	0.29	-1	27	27	27	496,979	ChIP	unknown
Robertson et al. 2007	human	HeLaS3-unstimulated-4-STAT1	0.92	1.45	0.23	-2	36	36	36	2,746,723	ChIP	unknown
Robertson et al. 2007	human	HeLaS3-unstimulated-5-STAT1	0.93	2.17	0.3	-1	27	27	27	1,447,320	ChIP	unknown
Robertson et al. 2007	human	HeLaS3-unstimulated-6-STAT1	0.93	2.05	0.28	-1	27	27	27	1,425,741	ChIP	unknown
Robertson et al. 2007	human	HeLaS3-unstimulated-7-STAT1	0.95	1.47	0.34	-1	27	27	27	2,452,058	ChIP	unknown
Welboren et al. 2009	human	MCF7-E2-ERa	0.7	12.72	1.74	2	32	32	32	9,428,987	ChIP	yes
Welboren et al. 2009	human	MCF7-Fulvestrant-ERa	0.83	5.17	1.22	1	32	32	32	6,243,484	ChIP	yes
Welboren et al. 2009	human	MCF7-mock-treated-ERa	0.67	6.52	2.9	2	32	32	32	1,722,599	ChIP	unknown
Welboren et al. 2009	human	MCF7-Tamoxifen-ERa	0.82	8.86	1.55	2	32	32	32	5,836,314	ChIP	yes
Visel et al. 2009; Gotea et al. 2010; Blow et al. 2010	mouse	Forebrain-p300	0.57	1.72	0.15	-2	36.32	38	36	4,842,793	ChIP	yes
Visel et al. 2009; Gotea et al. 2010; Blow et al. 2010	mouse	Limb-p300	0.73	2.16	0.15	-2	36	36	35	2,209,017	ChIP	yes
Visel et al. 2009; Gotea et al. 2010; Blow et al. 2010	mouse	Midbrain-p300	0.49	1.97	0.18	-2	36.25	38	36	5,942,773	ChIP	yes
Ho et al. 2009	mouse	mESC-Brg-J1	0.86	1.25	0.49	-1	25	25	25	12,146,582	ChIP	yes
Ho et al. 2009	mouse	mESC-IgG	0.91	1.17	0.51	0	25	25	25	14,118,667	IgG	no
Cuddapah et al. 2009	human	CD4+-CTCF	0.88	31.05	2.29	2	24	24	24	2,942,119	ChIP	yes
Cuddapah et al. 2009	human	HeLa-CTCF	0.93	5.22	1.29	1	24	24	24	3,294,793	ChIP	yes
Cuddapah et al. 2009	human	Jurkat-CTCF	0.91	4.22	1.01	1	25	25	25	4,367,791	ChIP	yes
Krebs et al. 2010	mouse	mESC-LUZP1	0.62	16.78	4.02	2	36	36	36	7,021,192	ChIP	yes
Krebs et al. 2010	mouse	mESC-mock	0.37	16.17	3.94	2	36	36	36	7,446,009	IgG	no
Corbo et al. 2010	mouse	NRL-KO-Crx-Rep1	0.69	1.81	1.18	1	36	36	36	12,527,332	ChIP	yes
Corbo et al. 2010	mouse	NRL-KO-Crx-Rep2	0.8	1.8	1.24	1	36	36	36	10,488,445	ChIP	yes
Corbo et al. 2010	mouse	NRL-KO-IgG-Rep1	0.75	1.58	0.9	0	36	36	36	12,160,830	IgG	no
Corbo et al. 2010	mouse	NRL-KO-IgG-Rep2	0.69	2.43	1.51	2	36	36	36	11,005,528	IgG	no
Corbo et al. 2010	mouse	WT-Crx-Rep1	0.89	3.69	0.63	0	36	36	36	4,302,798	ChIP	yes
Corbo et al. 2010	mouse	WT-Crx-Rep2	0.9	4.07	0.89	0	36	36	36	4,308,655	ChIP	yes
Corbo et al. 2010	mouse	WT-IgG-Rep1	0.92	2.53	0.42	-1	36	36	36	3,707,696	IgG	no
Ramagopalan et al. 2010	human	GM10855-Input	0.94	1.16	0.27	-1	36	36	36	11,412,903	Input	no
Ramagopalan et al. 2010	human	GM10855-unstimulated-rep1	0.87	1.41	0.56	0	36	36	36	13,520,376	ChIP	unknown
Ramagopalan et al. 2010	human	GM10855-unstimulated-rep2	0.88	1.47	0.55	0	36	36	36	10,791,763	ChIP	unknown
Ramagopalan et al. 2010	human	GM10855-vitaminD-rep1	0.89	1.76	0.83	0	36	36	36	13,970,589	ChIP	yes
Ramagopalan et al. 2010	human	GM10855-vitaminD-rep2	0.89	1.67	0.82	0	36	36	36	14,642,572	ChIP	yes
Ramagopalan et al. 2010	human	GM10861-Input	0.95	1.19	0.35	-1	36	36	36	11,404,257	Input	no
Ramagopalan et al. 2010	human	GM10861-unstimulated-rep1	0.93	1.39	0.56	0	36	36	36	10,157,583	ChIP	unknown

Continued on next page

Table S1 – *Continued from previous page*

Source	Species	Library	Complexity	NSC	RSC	QC	Ave. Read Length	Max. Read Length	Min. Read Length	Mapped reads	Type	Should exhibit read clustering
Ramagopalan et al. 2010	human	GM10861-unstimulated-rep2	0.93	1.52	0.67	0	36	36	36	7,922,208	ChIP	unknown
Ramagopalan et al. 2010	human	GM10861-vitaminD-rep1	0.92	1.88	0.95	0	36	36	36	10,649,722	ChIP	yes
Ramagopalan et al. 2010	human	GM10861-vitaminD-rep2	0.93	1.88	0.95	0	36	36	36	11,754,302	ChIP	yes
Wei et al. 2010	mouse	Th1-STAT4-KO-STAT4	0.84	2.05	1.25	1	36	36	36	9,339,036	ChIP	no
Wei et al. 2010	mouse	Th1-WT-STAT4	0.88	7.69	2.19	2	36	36	36	10,525,607	ChIP	yes
Wei et al. 2010	mouse	Th2-Normal-Rabbit-Serum	0.75	2.42	1.31	1	36	36	36	7,610,146	IgG	no
Wei et al. 2010	mouse	Th2-STAT6-KO-STAT6	0.89	2.38	1.37	1	36	36	36	9,734,600	ChIP	no
Wei et al. 2010	mouse	Th2-WT-STAT6	0.83	6.45	1.62	2	36	36	36	9,139,067	ChIP	yes
Schnetz et al. 2010	mouse	mES-CHD7	0.91	1.56	0.55	0	37	37	37	8,269,486	ChIP	yes
Schnetz et al. 2010	mouse	mES-p300	0.96	1.35	0.73	0	37	37	37	17,677,307	ChIP	yes
GSE22303	mouse	mES-B2-TBP	0.92	2.43	1.21	1	36	36	36	18,683,322	ChIP	yes
GSE22303	mouse	mES-B6-TBP	0.91	1.94	0.73	0	26	26	26	3,617,586	ChIP	yes
Lin et al. 2010	mouse	A12-E2A-6h-E47ER	0.93	3.41	0.61	0	36	36	36	2,776,323	ChIP	yes
Lin et al. 2010	mouse	E2AKO-E2A-1h-E47ER	0.68	9.12	1.13	1	36	36	36	5,948,823	ChIP	yes
Lin et al. 2010	mouse	E2AKO-E2A-6h-E47ER	0.96	4.34	0.59	0	36	36	36	2,196,108	ChIP	yes
Lin et al. 2010	mouse	EBFKO-E2A	0.92	1.72	0.53	0	36	36	36	9,159,853	ChIP	yes
Lin et al. 2010	mouse	Input2	0.93	1.15	0.11	-2	36	36	36	10,675,120	Input	no
Lin et al. 2010	mouse	RAG1KO-CTCF	0.91	15.22	2.31	2	36	36	36	4,804,275	ChIP	yes
Lin et al. 2010	mouse	RAG1KO-E2A	0.85	4.13	1.39	1	30	36	25	7,601,861	ChIP	yes
Lin et al. 2010	mouse	RAG1KO-EBF	0.81	10.08	1.23	1	36	36	36	2,935,481	ChIP	yes
Lin et al. 2010	mouse	RAG1KO-FOXO1-1	0.91	6.82	1.06	1	36	36	36	15,561,578	ChIP	yes
Durant et al. 2010	mouse	Th17-Stat3fl-fl-FoxP3-GFP-STAT3	0.81	2.61	1.36	1	36	36	36	12,871,479	ChIP	yes
Heinz et al. 2010	mouse	Bcell-input-ChIP-Seq	0.68	1.75	0.12	-2	36	36	36	11,410,688	Input	no
Heinz et al. 2010	mouse	Bcell-Oct2-ChIP-Seq	0.95	2.16	0.17	-2	36	36	36	2,296,228	ChIP	yes
Heinz et al. 2010	mouse	Bcell-PU.1-ChIP-Seq	0.92	5.56	4.62	2	36	36	36	8,207,220	ChIP	yes
Heinz et al. 2010	mouse	BirA-input-GW-ChIP-Seq	0.96	1.24	0.54	0	23	23	23	2,263,641	Input	no
Heinz et al. 2010	mouse	BLRP-LXRb-GW-ChIP-Seq	0.8	2.02	0.68	0	22.45	25	22	9,426,604	ChIP	yes
Heinz et al. 2010	mouse	BMDM.LXRDKO-PU.1-ChIP-Seq	0.97	9.26	2.72	2	23	23	23	2,410,527	ChIP	yes
Heinz et al. 2010	mouse	BMDM-PU.1-ChIP-Seq	0.93	10.78	1.97	2	36	36	36	9,617,221	ChIP	yes
Heinz et al. 2010	mouse	E2AKO-PU.1-bHLH-ER-ChIP-Seq	0.91	7.77	2.76	2	23	23	23	5,093,144	ChIP	yes
Heinz et al. 2010	mouse	E2AKO-PU.1-ChIP-Seq	0.89	10.21	2.94	2	23	23	23	3,615,197	ChIP	yes
Heinz et al. 2010	mouse	E2AKO-PU.1-E2A-ER-ChIP-Seq	0.88	8.28	2.67	2	23	23	23	4,724,664	ChIP	yes
Heinz et al. 2010	mouse	EBFKO-PU.1-ChIP-Seq	0.94	11.94	2.4	2	23	23	23	3,058,714	ChIP	yes
Heinz et al. 2010	mouse	PU.1KO-CEBPb-ChIP-Seq	0.89	4.52	1.42	1	23	23	23	4,179,430	ChIP	yes
Heinz et al. 2010	mouse	PU.1KO-PU.1-ChIP-Seq	0.88	2.79	0.53	0	23	23	23	4,615,899	ChIP	no
Heinz et al. 2010	mouse	PUER-CEBPb-0h-ChIP-Seq	0.92	8.61	2.14	2	23	23	23	4,672,159	ChIP	yes
Heinz et al. 2010	mouse	PUER-CEBPb-1h-ChIP-Seq	0.92	9.02	2.1	2	23	23	23	3,790,612	ChIP	yes
Heinz et al. 2010	mouse	PUER-CEBPb-24h-ChIP-Seq	0.89	10.77	2.54	2	23	23	23	4,625,986	ChIP	yes
Heinz et al. 2010	mouse	PUER-CEBPb-48h-ChIP-Seq	0.9	8.09	1.95	2	23	23	23	5,022,074	ChIP	yes
Heinz et al. 2010	mouse	PUER-CEBPb-6h-ChIP-Seq	0.89	9.43	2.06	2	23	23	23	4,417,004	ChIP	yes
Heinz et al. 2010	mouse	PUER-PU.1-0h-ChIP-Seq	0.94	4.68	0.57	0	23	23	23	2,053,953	ChIP	yes
Heinz et al. 2010	mouse	PUER-PU.1-1h-ChIP-Seq	0.92	12.06	2.58	2	23	23	23	2,541,096	ChIP	yes
Heinz et al. 2010	mouse	PUER-PU.1-24h-ChIP-Seq	0.9	18.69	2.85	2	23	23	23	3,403,839	ChIP	yes
Heinz et al. 2010	mouse	PUER-PU.1-48h-ChIP-Seq	0.9	14.68	3.42	2	23	23	23	4,138,465	ChIP	yes
Heinz et al. 2010	mouse	PUER-PU.1-6h-ChIP-Seq	0.9	16.62	2.83	2	23	23	23	3,477,600	ChIP	yes
Heinz et al. 2010	mouse	RAG1KO-PU.1-ChIP-Seq	0.86	12.16	2.26	2	23	23	23	6,302,473	ChIP	yes
Heinz et al. 2010	mouse	ThioMac-CEBPa-ChIP-Seq	0.92	5.55	2.96	2	23	23	23	7,067,160	ChIP	yes
Heinz et al. 2010	mouse	ThioMac-input-ChIP-Seq	0.93	1.25	0.11	-2	23.67	25	22	5,491,097	Input	no
Heinz et al. 2010	mouse	ThioMac-PU.1-ChIP-Seq	0.97	5.1	3.4	2	23	23	23	5,289,667	ChIP	yes
Steger et al. 2010	mouse	3T3-L1-0hr-CEBPb	0.51	5.34	1.6	2	36	36	36	11,295,935	ChIP	yes
Steger et al. 2010	mouse	3T3-L1-0hr-Input	0.94	3.23	0.46	-1	36	36	36	5,129,801	Input	no
Steger et al. 2010	mouse	3T3-L1-240hr-Input	0.95	3.48	0.55	0	36	36	36	5,019,654	Input	no
Steger et al. 2010	mouse	3T3-L1-24hr-Input	0.81	6.84	1.26	1	36	36	36	4,731,402	Input	no
Steger et al. 2010	mouse	3T3-L1-6hr-CEBPb	0.87	3.05	1.09	1	36	36	36	10,746,117	ChIP	yes
Steger et al. 2010	mouse	3T3-L1-6hr-GR	0.86	1.79	0.75	0	36	36	36	10,761,593	ChIP	yes
Steger et al. 2010	mouse	3T3-L1-6hr-Input	0.9	1.4	0.56	0	36	36	36	11,352,790	Input	no
GSE21916	human	H9-IgG	0.93	1.53	0.33	-1	26	26	26	4,499,095	IgG	no
GSE21916	human	H9-Oct4-replicate-2	0.97	1.97	0.83	0	36	36	36	4,556,649	ChIP	yes
GSE21916	human	H9-Oct4-technical-replicate-1	0.92	1.81	0.48	-1	26	26	26	4,187,685	ChIP	yes
GSE21916	human	H9-Oct4-technical-replicate-2	0.95	1.85	0.6	0	36	36	36	4,119,980	ChIP	yes
Kassouf et al. 2010	mouse	RER-SCL	0.72	1.94	0.91	0	36	36	36	5,208,895	ChIP	no
Kassouf et al. 2010	mouse	RER-SCL-no-AB	0.51	9.5	1.38	1	36	36	36	4,571,728	IgG	no
Kassouf et al. 2010	mouse	WT-no-AB	0.81	5.94	1.05	1	36	36	36	5,312,397	IgG	no
Kassouf et al. 2010	mouse	WT-SCL	0.68	2.77	1.4	1	36	36	36	4,154,252	ChIP	yes
MacIsaac et al. 2010	mouse	CEBPa-3T3-L1	0.93	2.43	0.75	0	35	35	35	4,326,509	ChIP	yes
MacIsaac et al. 2010	mouse	CEBPa-liver	0.9	14.08	2.03	2	35	35	35	4,595,713	ChIP	yes
MacIsaac et al. 2010	mouse	E2F4-liver	0.92	16.44	2.26	2	35	35	35	2,214,727	ChIP	yes
MacIsaac et al. 2010	mouse	FOXA1-liver	0.54	12.93	3.19	2	35	35	35	3,968,403	ChIP	yes
MacIsaac et al. 2010	mouse	FOXA2-liver	0.95	6.49	1.99	2	35	35	35	6,593,622	ChIP	yes
MacIsaac et al. 2010	mouse	p300-3T3-L1	0.96	1.74	0.77	0	35	35	35	3,575,940	ChIP	yes
MacIsaac et al. 2010	mouse	p300-liver	0.95	6.6	2.79	2	35	35	35	4,718,264	ChIP	yes

Continued on next page

Table S1 – *Continued from previous page*

Source	Species	Library	Complexity	NSC	RSC	QC	Ave. Read Length	Max. Read Length	Min. Read Length	Mapped reads	Type	Should exhibit read clustering
MacIsaac et al. 2010	mouse	Sample-control-reads-3T3-L1	0.29	1.46	0.47	-1	35	35	35	2,767,084	Input	no
MacIsaac et al. 2010	mouse	Sample-control-reads-cerebellum	0.93	1.9	0.52	0	35	35	35	5,139,906	Input	no
MacIsaac et al. 2010	mouse	Sample-control-reads-liver	0.64	23.8	2.7	2	35	35	35	5,270,015	Input	no
Vivar et al. 2010	human	U2OS-ERb-Doxy-nonspecificAntibodyIgG-rep1	0.96	1.33	0.26	-1	26	26	26	2,576,564	IgG	no
Vivar et al. 2010	human	U2OS-ERb-Doxy-specificAntibody-rep1	0.95	2.8	0.7	0	26	26	26	2,749,749	ChIP	yes
Vivar et al. 2010	human	U2OS-ERb-DoxyE2-nonspecificAntibodyIgG-rep1	0.95	1.43	0.31	-1	26	26	26	2,880,960	IgG	no
Vivar et al. 2010	human	U2OS-ERb-DoxyE2-specificAntibody-rep1	0.95	6.1	1.13	1	26	26	26	2,638,244	ChIP	yes
Fortschegger et al. 2010	human	Input-DNA-Hs68+FBS	0.97	1.32	0.27	-1	40	40	40	8,279,525	Input	no
Fortschegger et al. 2010	human	Input-DNA-Hs68-FBS	0.97	1.34	0.3	-1	40	40	40	7,059,465	Input	no
Fortschegger et al. 2010	human	Normal-IgG-293T	0.94	1.21	0.22	-2	50	50	50	7,860,447	IgG	no
Fortschegger et al. 2010	human	Normal-IgG-HeLa	0.92	1.76	0.55	0	50	50	50	7,000,514	IgG	no
Fortschegger et al. 2010	human	PHF8-293T	0.96	1.71	0.71	0	50	50	50	7,015,757	ChIP	yes
Fortschegger et al. 2010	human	PHF8-HeLa	0.95	2.74	1.13	1	50	50	50	6,982,792	ChIP	yes
Fortschegger et al. 2010	human	PHF8-Hs68+FBS	0.94	1.89	0.69	0	40	40	40	7,339,329	ChIP	yes
Fortschegger et al. 2010	human	PHF8-Hs68-FBS	0.9	1.75	0.51	0	35	35	35	11,313,461	ChIP	yes
GSE15844	mouse	MEF-NFIC-KO-NFI	0.29	7.55	1.85	2	35	35	35	10,794,407	ChIP	no
GSE15844	mouse	MEF-WT-Input	0.74	1.42	0.28	-1	36	36	36	5,483,670	Input	no
GSE15844	mouse	MEF-WT-NFI	0.34	5.81	1.72	2	35	35	35	9,746,594	ChIP	yes
Kim et al. 2010	mouse	ChIP-CBP-ab2832-KCl-b1	0.88	2.46	0.88	0	33	33	33	1,742,367	ChIP	yes
Kim et al. 2010	mouse	ChIP-CBP-ab2832-KCl-b2	0.9	1.9	0.31	-1	33	33	33	1,350,494	ChIP	yes
Kim et al. 2010	mouse	ChIP-CBP-ab2832-un-b1	0.11	2.19	0.23	-2	33	33	33	13,062,901	ChIP	yes
Kim et al. 2010	mouse	ChIP-CBP-Millipore-KCl-b1	0.14	2.55	0.19	-2	33	33	33	21,475,816	ChIP	yes
Kim et al. 2010	mouse	ChIP-CBP-Millipore-un-b1	0.14	3.57	0.22	-2	33	33	33	12,767,854	ChIP	yes
Kim et al. 2010	mouse	ChIP-CREB-SC-KCl-b1	0.25	1.6	0.2	-2	33	33	33	12,606,497	ChIP	yes
Kim et al. 2010	mouse	ChIP-CREB-SC-KCl-b2	0.11	2.71	0.25	-1	33	33	33	14,186,880	ChIP	yes
Kim et al. 2010	mouse	ChIP-CREB-SC-un-b1	0.47	1.39	0.25	-2	33	33	33	11,723,416	ChIP	yes
Kim et al. 2010	mouse	ChIP-CREB-SC-un-b2	0.11	3.54	0.43	-1	33	33	33	11,668,187	ChIP	yes
Kim et al. 2010	mouse	ChIP-input-KCl-b1	0.22	2.19	0.19	-2	33	33	33	29,829,497	Input	no
Kim et al. 2010	mouse	ChIP-input-KCl-b2	0.55	1.29	0.3	-1	33	33	33	11,407,302	Input	no
Kim et al. 2010	mouse	ChIP-input-un-b1	0.57	1.86	0.16	-2	33	33	33	4,413,802	Input	no
Kim et al. 2010	mouse	ChIP-input-un-b2	0.59	1.28	0.39	-1	33	33	33	2,034,854	Input	no
Kim et al. 2010	mouse	ChIP-Npas4-KCl-b1	0.3	3.33	1.38	1	33	33	33	6,262,184	ChIP	yes
Kim et al. 2010	mouse	ChIP-Npas4-KCl-b2	0.7	2.39	0.92	0	33	33	33	3,474,756	ChIP	yes
Kim et al. 2010	mouse	ChIP-Npas4-un-b1	0.39	1.84	0.21	-2	33	33	33	12,918,805	ChIP	yes
Kim et al. 2010	mouse	ChIP-SRF-SC-KCl-b1	0.88	3.77	0.28	-1	33	33	33	1,953,844	ChIP	yes
Kim et al. 2010	mouse	ChIP-SRF-SC-KCl-b2	0.86	2.72	0.46	-1	33	33	33	7,001,063	ChIP	yes
Kim et al. 2010	mouse	ChIP-SRF-SC-un-b1	0.89	2.58	0.47	-1	33	33	33	2,076,216	ChIP	yes
Kim et al. 2010	mouse	ChIP-SRF-SC-un-b2	0.87	2.2	0.98	0	33	33	33	8,797,223	ChIP	yes
Lefterova et al. 2010	mouse	Lefterova-ad-PPARg	0.85	2.18	0.73	0	36	36	36	5,258,157	ChIP	yes
Lefterova et al. 2010	mouse	Lefterova-mac-CEBPb	0.67	11.03	1.5	2	36	36	36	5,717,739	ChIP	yes
Lefterova et al. 2010	mouse	Lefterova-mac-PPARg-1	0.87	1.59	0.69	0	36	36	36	10,646,239	ChIP	yes
Lefterova et al. 2010	mouse	Lefterova-mac-PU.1	0.86	13.27	1.64	2	36	36	36	6,261,063	ChIP	yes
Tallack et al. 2010	mouse	KLF1-Input-2	0.96	9.63	0.58	0	48	48	48	10,405,126	Input	no
Tallack et al. 2010	mouse	KLF1-2	0.68	1.28	0.18	-2	33	33	33	10,757,339	ChIP	yes
Tallack et al. 2010	mouse	KLF1-3	0.55	1.39	0.47	-1	48	48	48	17,728,355	ChIP	yes
Tallack et al. 2010	mouse	KLF1-Input-3	0.65	1.23	0.4	-1	33	33	33	548,382	Input	no
Rahl et al. 2010	mouse	mES-Ctr9	0.96	1.37	0.97	0	26	26	26	5,468,214	ChIP	yes
Rahl et al. 2010	mouse	mES-NelfA	0.7	2.42	1.42	1	36	36	36	3,643,555	ChIP	yes
Rahl et al. 2010	mouse	mES-Spt5	0.95	1.59	0.94	0	26	26	26	5,595,215	ChIP	yes
Ramos et al. 2010	human	T98G-CBP-T0	0.94	1.55	0.31	-1	32	32	32	4,047,183	ChIP	yes
Ramos et al. 2010	human	T98G-CBP-T30-1	0.94	1.63	0.4	-1	32	32	32	4,885,700	ChIP	yes
Ramos et al. 2010	human	T98G-CBP-T30-2	0.84	1.75	2.14	2	32	32	32	5,034,834	ChIP	yes
Ramos et al. 2010	human	T98G-p300-T0	0.96	1.79	0.85	0	32	32	32	5,119,057	ChIP	yes
Ramos et al. 2010	human	T98G-p300-T30-1	0.97	2.22	1.19	1	32	32	32	5,191,684	ChIP	yes
Ramos et al. 2010	human	T98G-p300-T30-2	0.87	1.95	3.44	2	32	32	32	5,159,200	ChIP	yes
Kunarso et al. 2010	human	hESC-CTCF	0.92	15.2	1.62	2	37	37	37	10,828,759	ChIP	yes
Kunarso et al. 2010	human	hESC-NANOG	0.94	3.91	1.42	1	36.18	37	36	10,240,400	ChIP	yes
Kunarso et al. 2010	human	hESC-Nanog-and-CTCF-control	0.96	1.25	0.19	-2	37	37	37	8,641,430	Input	no
Kunarso et al. 2010	human	hESC-OCT4	0.98	1.94	0.42	-1	30.07	36	26	11,288,800	ChIP	yes
Kunarso et al. 2010	human	hESC-Oct4-control	0.95	1.26	0.4	-1	36	36	36	8,560,581	Input	no
Johannes et al. 2010	human	HeLa-BTAF	0.79	13.25	2.7	2	33	33	33	2,654,681	ChIP	yes
Johannes et al. 2010	human	HeLa-GAPDH	0.84	2.51	0.02	-2	33	33	33	953,719	IgG	no
Hu et al. 2010	human	MCF7-E2-ER	0.8	10.66	1.28	1	36	36	36	1,656,740	ChIP	yes
Hu et al. 2010	human	MCF7-ethl-ER	0.81	3.76	0.87	0	36	36	36	2,857,720	ChIP	unknown
Heng et al. 2010	mouse	mESC-HA-1	0.97	1.25	0.83	0	35	35	35	14,266,600	IgG	no
Heng et al. 2010	mouse	mESC-HA-Nr5a2-1	0.85	1.54	0.36	-1	35	35	35	9,395,231	ChIP	yes
Chicas et al. 2010	human	IMR90-Growing-cells-pRb-experiment-1-1	0.78	2.94	0.41	-1	36	36	36	6,181,869	ChIP	yes

Continued on next page

Table S1 – *Continued from previous page*

Source	Species	Library	Complexity	NSC	RSC	QC	Ave. Read Length	Max. Read Length	Min. Read Length	Mapped reads	Type	Should exhibit read clustering
Chicas et al. 2010	human	IMR90-Mock-1	0.85	2.18	0.13	-2	36	36	36	3,317,485	IgG	no
Chicas et al. 2010	human	IMR90-Quiescent-cells-p130	0.92	4.12	0.95	0	36	36	36	3,753,591	ChIP	yes
Chicas et al. 2010	human	IMR90-Quiescent-cells-pRb-experiment-1-1	0.92	3.3	0.38	-1	36	36	36	1,441,212	ChIP	yes
Chicas et al. 2010	human	IMR90-Quiescent-cells-pRb-experiment-2	0.93	2.43	0.05	-2	36	36	36	4,608,677	ChIP	yes
Chicas et al. 2010	human	IMR90-Quiescent-cells-Rb-shRNA-p130	0.78	2.2	0.24	-2	36	36	36	5,348,557	ChIP	yes
Chicas et al. 2010	human	IMR90-Quiescent-cells-Rb-shRNA-Rb	0.85	2.42	0.38	-1	36	36	36	1,114,921	ChIP	no
Chicas et al. 2010	human	IMR90-Senescent-cells-p130	0.94	4.21	1.21	1	36	36	36	4,388,261	ChIP	yes
Chicas et al. 2010	human	IMR90-Senescent-cells-pRb-experiment-1-1	0.93	3.63	0.39	-1	36	36	36	3,867,162	ChIP	yes
Chicas et al. 2010	human	IMR90-Senescent-cells-pRb-experiment-2	0.91	2.29	0.22	-2	36	36	36	4,109,281	ChIP	yes
Chicas et al. 2010	human	IMR90-Senescent-cells-Rb-shRNA-p130	0.78	3.98	1.26	1	36	36	36	4,232,255	ChIP	yes
Chicas et al. 2010	human	IMR90-Senescent-cells-Rb-shRNA-pRb	0.89	2.02	0.23	-2	36	36	36	2,813,407	ChIP	no
Martinez et al. 2010	mouse	Input	0.51	2.26	2.59	2	36	36	36	14,617,059	Input	no
Martinez et al. 2010	mouse	RAP1-ko1-RAP1	0.09	20.55	2.72	2	36	36	36	11,542,127	ChIP	no
Martinez et al. 2010	mouse	RAP1-ko2-RAP1	0.18	21.54	3.88	2	36	36	36	7,585,528	ChIP	no
Martinez et al. 2010	mouse	WT-1-RAP1	0.26	11.02	2.45	2	36	36	36	11,249,746	ChIP	yes
Martinez et al. 2010	mouse	WT-2-RAP1	0.19	11.05	2.88	2	36	36	36	11,856,303	ChIP	yes
Qi et al. 2010	human	HeLa-PHF8	0.9	1.66	0.78	0	25	25	25	9,252,893	ChIP	yes
Woodfield et al. 2010	human	MCF7-IgG-control	0.97	1.38	0.63	0	40	40	40	8,158,903	IgG	no
Woodfield et al. 2010	human	MCF7-TFAP2C	0.93	7.1	1.74	2	40	40	40	8,188,674	ChIP	yes
Kagey et al. 2010	mouse	MEF-Med12-Rep1	0.85	1.58	0.46	-1	36	36	36	8,167,440	ChIP	yes
Kagey et al. 2010	mouse	MEF-Med1-Rep1	0.94	2.03	0.82	0	36	36	36	7,326,311	ChIP	yes
Kagey et al. 2010	mouse	MEF-Smc1-Rep1	0.62	6.04	2.96	2	36	36	36	9,601,525	ChIP	yes
Kagey et al. 2010	mouse	MEF-Smc1-Rep2	0.94	1.34	0.9	0	36	36	36	22,977,719	ChIP	yes
Kagey et al. 2010	mouse	mESC-CTCF-Rep1	0.94	7.28	1.29	1	36	36	36	3,966,359	ChIP	yes
Kagey et al. 2010	mouse	mESC-CTCF-Rep2	0.85	1.73	1.4	1	36	36	36	4,953,685	ChIP	yes
Kagey et al. 2010	mouse	mESC-Med12-051809-ChipSeq	0.84	7.16	1.9	2	36	36	36	22,763,608	ChIP	yes
Kagey et al. 2010	mouse	mESC-Med12-Rep2	0.63	1.8	1.12	1	36	36	36	12,861,074	ChIP	yes
Kagey et al. 2010	mouse	mESC-Med1-Rep1	0.92	2.27	1.68	2	36	36	36	18,346,720	ChIP	yes
Kagey et al. 2010	mouse	mESC-Med1-Rep2	0.94	1.73	1.04	1	36	36	36	18,725,724	ChIP	yes
Kagey et al. 2010	mouse	mESC-Nipbl-Rep1	0.26	1.33	0.54	0	36	36	36	6,241,538	ChIP	yes
Kagey et al. 2010	mouse	mESC-Nipbl-Rep2	0.96	1.54	0.99	0	36	36	36	12,668,428	ChIP	yes
Kagey et al. 2010	mouse	mESC-Smc1-Rep1	0.96	3.37	1.88	2	36	36	36	21,733,223	ChIP	yes
Kagey et al. 2010	mouse	mESC-Smc1-Rep2	0.95	3.29	1.57	2	36	36	36	4,936,893	ChIP	yes
Kagey et al. 2010	mouse	mESC-Smc3-Rep3	0.89	3.86	1.81	2	36	36	36	21,491,459	ChIP	yes
Kagey et al. 2010	mouse	mESC-Smc3-Rep4	0.89	4.34	2.26	2	36	36	36	21,522,393	ChIP	yes
Kagey et al. 2010	mouse	mES-WCE	0.93	1.38	0.31	-1	36	36	36	3,669,758	Input	no
Kouwenhoven et al. 2010	human	Keratinocytes-p63-1	0.96	19.62	2.46	2	32	32	32	2,722,489	ChIP	yes
Kouwenhoven et al. 2010	human	Keratinocytes-p63-2	0.96	4.51	2.51	2	32	32	32	5,588,217	ChIP	yes
Kouwenhoven et al. 2010	human	Keratinocytes-p63-3	0.81	9.05	5.19	2	35	35	35	20,435,516	ChIP	yes
Cao et al. 2010	human	RD-Input	0.82	1.34	0.33	-1	40	40	40	6,587,573	Input	no
Cao et al. 2010	human	RD-pFM2-1	0.76	1.9	0.63	0	40	40	40	8,593,218	ChIP	yes
Cao et al. 2010	human	Rh4-Input-1	0.82	1.35	0.36	-1	38.7	40	36	20,270,400	Input	no
Cao et al. 2010	human	Rh4-pFM2-1	0.74	2.33	0.9	0	38.83	40	36	20,920,563	ChIP	yes
Blow et al. 2010	mouse	Heart-p300	0.85	1.77	0.16	-2	36	36	36	1,531,274	ChIP	yes
Blow et al. 2010	mouse	Midbrain-p300	0.87	1.34	0.21	-2	36	36	36	6,406,542	ChIP	yes
Sehat et al. 2010	human	DFB-IGF1R	0.9	1.56	0.13	-2	36	36	36	3,664,071	ChIP	yes
Liu et al. 2010	human	E2F1-HeLa	0.96	1.91	0.81	0	36	36	36	8,595,301	ChIP	yes
Liu et al. 2010	human	PHF8-HeLa-unsyn	0.97	2.21	0.84	0	36	36	36	3,841,047	ChIP	yes
Liu et al. 2010	human	SMC4-HeLa-M	0.62	2.43	0.65	0	36	36	36	9,809,944	ChIP	yes
Tang et al. 2010	human	K562-PMA-Egr1	0.84	1.62	0.21	-2	33	33	33	3,581,558	ChIP	yes
Jung et al. 2010	mouse	iHoxc9-Day5	0.97	1.35	0.79	0	36	36	36	10,149,860	ChIP	yes
Jung et al. 2010	mouse	WCE-Day5	0.94	1.37	0.82	0	36	36	36	15,043,390	Input	no
Vermeulen et al. 2010	human	BAP18-GFP-HeLa-rep1	0.8	2.1	1.6	2	35	35	35	11,153,198	ChIP	yes
Vermeulen et al. 2010	human	BAP18-GFP-HeLa-rep2	0.83	1.91	2.1	2	35	35	35	28,580,771	ChIP	yes
Vermeulen et al. 2010	human	GATAD1-GFP-HeLa-rep1	0.9	1.71	2.69	2	35	35	35	5,413,596	ChIP	yes
Vermeulen et al. 2010	human	GATAD1-GFP-HeLa-rep2	0.81	2.4	1.83	2	35	35	35	12,596,319	ChIP	yes
Vermeulen et al. 2010	human	LRWD1-GFP-HeLa	0.88	2.29	0.98	0	35	35	35	11,634,470	ChIP	yes
Vermeulen et al. 2010	human	N-PAC-GFP-HeLa-rep1	0.85	2.01	2.46	2	35	35	35	5,436,726	ChIP	yes
Vermeulen et al. 2010	human	N-PAC-GFP-HeLa-rep2	0.77	2.78	2.81	2	35	35	35	12,669,139	ChIP	yes
Vermeulen et al. 2010	human	PHF8-GFP-HeLa-rep1	0.88	1.7	2.13	2	35	35	35	4,896,779	ChIP	yes
Vermeulen et al. 2010	human	PHF8-GFP-HeLa-rep2	0.83	1.79	2.2	2	35	35	35	29,180,126	ChIP	yes
Vermeulen et al. 2010	human	Sgf29-GFP-HeLa-rep1	0.87	2.36	1.45	1	35	35	35	12,636,931	ChIP	yes
Vermeulen et al. 2010	human	Sgf29-GFP-HeLa-rep2	0.87	1.84	1.98	2	35	35	35	29,275,648	ChIP	yes

Continued on next page

Table S1 – *Continued from previous page*

Source	Species	Library	Complexity	NSC	RSC	QC	Ave. Read Length	Max. Read Length	Min. Read Length	Mapped reads	Type	Should exhibit read clustering
Vermeulen et al. 2010	human	TRRAP-GFP-HeLa-rep1	0.74	3.43	3.94	2	35	35	35	7,851,229	ChIP	yes
Vermeulen et al. 2010	human	TRRAP-GFP-HeLa-rep2	0.87	1.71	1.76	2	35	35	35	29,410,330	ChIP	yes
Vermeulen et al. 2010	human	wt-negative-control-HeLa	0.87	1.88	1.09	1	35	35	35	10,851,096	Input	no
Chi et al. 2010	human	GIST48-ETV1	0.95	2.79	0.97	0	36	36	36	10,740,357	ChIP	yes
Chi et al. 2010	human	GIST48-Input	0.98	1.12	0.27	-1	36	36	36	15,177,140	Input	no
Chia et al. 2010	human	hESC-Input	0.98	1.48	0.82	0	35	35	35	17,097,337	Input	no
Chia et al. 2010	human	hESC-PRDM14	0.95	1.87	0.67	0	35	35	35	14,268,098	ChIP	no
Palii et al. 2010	human	Erythroid-TAL1	0.94	6.12	0.9	0	37	37	37	6,882,358	ChIP	yes
Palii et al. 2010	human	Jurkat-IgG	0.97	1.48	0.16	-2	37	37	37	4,760,148	IgG	no
Palii et al. 2010	human	Jurkat-TAL1	0.94	2.58	0.45	-1	37	37	37	6,151,678	ChIP	yes
Lee et al. 2010	human	GM06990-E2F4	0.95	1.99	0.5	0	36	36	36	2,845,819	ChIP	yes
Lee et al. 2010	human	GM06990-Input	0.98	1.09	0.1	-2	36	36	36	7,164,483	Input	no
Law et al. 2010	human	ATRX-Human-Erythroid	0.98	1.83	0.31	-1	36	36	36	1,481,778	ChIP	yes
Law et al. 2010	human	ATRX-Human-Erythroid-Input	0.86	1.69	0.44	-1	36	36	36	2,815,016	Input	no
Law et al. 2010	mouse	ATRX-Mouse-ES	0.86	1.52	0.39	-1	51	51	51	47,903,467	ChIP	yes
Law et al. 2010	mouse	ATRX-Mouse-ES-Input	0.46	1.61	2.14	2	51	51	51	24,366,842	Input	no
Yao et al. 2010	human	HeLa-Input	0.97	1.17	0.51	0	36	36	36	44,239,692	Input	no
Yao et al. 2010	human	HeLa-p68	0.88	1.39	0.53	0	36	36	36	29,417,892	ChIP	yes
Verzi et al. 2010	human	Caco2-differentiated-CDX2	0.86	3.41	1.53	2	40	40	40	12,916,083	ChIP	yes
Verzi et al. 2010	human	Caco2-differentiated-GATA6	0.86	2.75	0.73	0	40	40	40	14,079,635	ChIP	unknown
Verzi et al. 2010	human	Caco2-differentiated-HNF4A	0.9	6.15	1.42	1	40	40	40	5,599,576	ChIP	yes
Verzi et al. 2010	human	Caco2-Input	0.79	2.68	1.27	1	40	40	40	10,777,726	Input	no
Verzi et al. 2010	human	Caco2-proliferating-CDX2	0.87	3.04	1.24	1	40	40	40	11,527,010	ChIP	unknown
Verzi et al. 2010	human	Caco2-proliferating-GATA6	0.77	12.01	1.6	2	40	40	40	7,337,182	ChIP	yes
Verzi et al. 2010	human	Caco2-proliferating-HNF4A	0.76	6.64	1.55	2	40	40	40	9,186,141	ChIP	yes
Barish et al. 2010	mouse	macrophage-BCL6	0.73	3.1	1.36	1	42	42	42	14,741,775	ChIP	yes
Barish et al. 2010	mouse	macrophage-BCL6-LPS-1	0.59	2.47	1.38	1	36	36	36	12,161,935	ChIP	unknown
Barish et al. 2010	mouse	macrophage-BCL6-LPS-2	0.52	1.99	1.02	1	42	42	42	19,613,630	ChIP	unknown
Barish et al. 2010	mouse	macrophage-Bcl6-REP2	0.43	8.05	3.11	2	36	36	36	10,772,781	ChIP	yes
Barish et al. 2010	mouse	macrophage-IgG	0.59	3.32	1.14	1	36	36	36	11,046,455	IgG	no
Barish et al. 2010	mouse	macrophage-Input	0.96	1.33	0.5	-1	36	36	36	14,265,664	Input	no
Barish et al. 2010	mouse	macrophage-p65	0.85	3.3	2.23	2	42	42	42	13,878,454	ChIP	no
Barish et al. 2010	mouse	macrophage-p65-LPS-1	0.71	2.6	1.23	1	43	43	43	12,731,143	ChIP	yes
Barish et al. 2010	mouse	macrophage-p65-LPS-2	0.75	1.95	1.2	1	42	42	42	10,819,755	ChIP	yes
Mahony et al. 2010	mouse	HBG3-RAR-Day2+8hrsRA-1	0.67	1.6	0.6	0	26	26	26	16,947,890	ChIP	yes
Mahony et al. 2010	mouse	HBG3-RAR-Day2-1	0.7	1.87	0.76	0	26	26	26	19,693,750	ChIP	yes
Mahony et al. 2010	mouse	HBG3-WCE-Day2	0.92	1.52	0.15	-2	26	26	26	2,570,671	Input	no
Mahony et al. 2010	mouse	HBG3-WCE-Day3	0.94	1.44	0.2	-2	26	26	26	3,038,741	Input	no
Yu et al. 2010	human	HPC-GABPa	0.73	7	1.7	2	24	24	24	3,036,253	ChIP	yes
Yu et al. 2010	human	HPC-IgG	0.45	15.8	2.15	2	25	25	25	2,762,252	IgG	no
Rada-Iglesias et al. 2010	human	ESC-BRG1	0.95	1.66	1.37	1	36	36	36	16,085,353	ChIP	yes
Rada-Iglesias et al. 2010	human	ESC-input	0.95	1.33	0.98	0	36	36	36	14,508,164	Input	no
Rada-Iglesias et al. 2010	human	ESC-p300	0.92	2.01	2.76	2	36	36	36	12,822,655	ChIP	yes
Rada-Iglesias et al. 2010	human	NEC-input	0.97	1.34	0.65	0	36	36	36	21,774,646	Input	no
Rada-Iglesias et al. 2010	human	NEC-p300	0.94	1.71	0.58	0	36	36	36	13,264,013	ChIP	yes
Gu et al. 2010	human	MCF7-control-ERa	0.79	1.57	0.24	-2	36	36	36	4,385,795	ChIP	no
Gu et al. 2010	human	MCF7-E2-ERa	0.8	1.81	0.28	-1	36	36	36	5,785,635	ChIP	yes
Ma et al. 2010	mouse	mESC-FLAG-HA	0.93	1.59	0.59	0	36	36	36	6,257,485	IgG	no
Ma et al. 2010	mouse	mESC-Input	0.9	1.36	1.23	1	36	36	36	8,480,128	Input	no
Ma et al. 2010	mouse	mESC-Prdm14	0.84	5.1	2.59	2	36	36	36	10,899,040	ChIP	yes
Schlesinger et al. 2010	mouse	HL1-SRF	0.93	1.53	1.12	1	36	36	36	5,086,170	ChIP	yes
Li et al. 2010	mouse	Lin–Gata2	0.85	1.89	0.88	0	25	25	25	7,512,398	ChIP	yes
Li et al. 2010	mouse	Lin–IgG	0.75	1.87	0.2	-2	25	25	25	3,211,969	IgG	no
Li et al. 2010	mouse	Lin–Ldb1	0.71	5.7	1.95	2	25	25	25	4,251,705	ChIP	yes
Li et al. 2010	mouse	Lin–Tal1	0.81	4.15	1.77	2	36	36	36	11,482,776	ChIP	yes
Kong et al. 2010	human	ECC1-E2-ERa	0.95	1.65	0.37	-1	31.26	36	26	7,178,094	ChIP	yes
Kong et al. 2010	human	ECC1-EtOH-ERa	0.94	1.35	0.21	-2	26	26	26	11,049,926	ChIP	no
Kong et al. 2010	human	ECC1-Input	0.98	1.19	0.19	-2	30.16	36	26	7,631,501	Input	no
Kong et al. 2010	human	Ishikawa-E2-ERa	0.97	1.42	0.41	-1	30.73	36	26	10,438,320	ChIP	yes
Kong et al. 2010	human	Ishikawa-EtOH-ERa	0.97	1.38	0.36	-1	30.55	36	26	10,175,702	ChIP	no
Kong et al. 2010	human	Ishikawa-Input	0.98	1.19	0.37	-1	26	26	26	21,437,974	Input	no
Kong et al. 2010	human	MCF7-E2-ERa	0.95	5.96	1.63	2	26	26	26	9,652,711	ChIP	yes
Kong et al. 2010	human	MCF7-EtOH-ERa	0.97	1.23	0.4	-1	33.21	36	26	14,488,769	ChIP	no
Kong et al. 2010	human	MCF7-Input	0.97	1.17	0.18	-2	26	26	26	8,379,328	Input	no
Kong et al. 2010	human	T47D-E2-ERa	0.97	2.84	1.13	1	26	26	26	10,608,916	ChIP	yes
Kong et al. 2010	human	T47D-EtOH-ERa	0.97	1.21	0.29	-1	33.66	36	26	13,430,557	ChIP	no
Kong et al. 2010	human	T47D-Input	0.96	1.18	0.19	-2	26	26	26	12,933,672	Input	no
Yang et al. 2010	human	MCF7-IgG	0.1	14.93	1.71	2	36	36	36	5,500,498	IgG	no
Yang et al. 2010	human	MCF7-TDRD3	0.34	3.12	1.25	1	36	36	36	27,147,620	ChIP	yes
Fang et al. 2011	human	LN229-IgG	0.6	2.01	1.53	2	40	40	40	455,630	IgG	no
Fang et al. 2011	human	LN229-Sox2	0.65	1.8	1.04	1	40	40	40	932,166	ChIP	yes

Continued on next page

Table S1 – *Continued from previous page*

Source	Species	Library	Complexity	NSC	RSC	QC	Ave. Read Length	Max. Read Length	Min. Read Length	Mapped reads	Type	Should exhibit read clustering
van Heeringen et al. 2011	xaenopus	TBP-ChIPSeq	0.83	N/A	N/A	N/A	32	32	32	6,569,902	ChIP	yes
GSE26680	mouse	mES-MCAF1	0.86	1.63	1.04	1	36	36	36	13,907,040	ChIP	yes
GSE26680	mouse	mES-REST	0.86	2.61	1.01	1	26	26	26	4,159,486	ChIP	yes
GSE26680	mouse	mES-Ring1b	0.92	1.23	0.43	-1	26	26	26	3,785,138	ChIP	yes
Teo et al. 2011	human	hESC-48h-endodiff-EOMES-XL-eps1and2	0.74	4.08	1.67	2	36	36	36	33,687,700	ChIP	yes
Teo et al. 2011	human	hESC-Input-XL	0.98	1.37	0.44	-1	36	36	36	7,422,963	Input	no
Joseph et al. 2011; Kong et al. 2010	human	MCF7-DMSO-cFos-1	0.93	1.31	0.43	-1	36	36	36	18,781,755	ChIP	yes
Joseph et al. 2011; Kong et al. 2010	human	MCF7-DMSO-cJun-1	0.96	1.31	0.37	-1	36	36	36	14,827,454	ChIP	yes
Joseph et al. 2011; Kong et al. 2010	human	MCF7-DMSO-FOXA1-1	0.95	1.51	0.63	0	36	36	36	14,414,733	ChIP	yes
Joseph et al. 2011; Kong et al. 2010	human	MCF7-E2-cFos-1	0.95	1.48	0.68	0	31	31	31	12,684,762	ChIP	yes
Joseph et al. 2011; Kong et al. 2010	human	MCF7-E2-cJun-1	0.95	1.31	0.4	-1	36	36	36	18,012,142	ChIP	yes
Joseph et al. 2011; Kong et al. 2010	human	MCF7-E2-FOXA1-1	0.93	2.52	1.07	1	36	36	36	15,884,461	ChIP	yes
Joseph et al. 2011; Kong et al. 2010	human	T47D-DMSO-FOXA1	0.94	5.13	1.78	2	36	36	36	14,981,282	ChIP	yes
Joseph et al. 2011; Kong et al. 2010	human	T47D-E2-FOXA1-1	0.94	2.27	0.92	0	36	36	36	11,819,434	ChIP	yes
Novershtern et al. 2011	human	HSPC-Ikaros-rep1	0.74	2.04	0.36	-1	36	36	36	3,228,102	ChIP	yes
Novershtern et al. 2011	human	HSPC-Ikaros-rep2	0.78	1.98	0.31	-1	36	36	36	2,635,528	ChIP	yes
Novershtern et al. 2011	human	HSPC-MEIS1-rep1	0.46	1.85	0.34	-1	36	36	36	9,565,937	ChIP	yes
Novershtern et al. 2011	human	HSPC-MEIS1-rep2	0.29	1.84	0.36	-1	36	36	36	12,342,658	ChIP	yes
Novershtern et al. 2011	human	HSPC-MEIS1-rep3	0.46	1.78	0.34	-1	36	36	36	10,465,042	ChIP	yes
Novershtern et al. 2011	human	HSPC-Pu.1-rep1	0.6	3.44	1.05	1	36	36	36	4,940,474	ChIP	yes
Novershtern et al. 2011	human	HSPC-Pu.1-rep2	0.61	3.19	1.03	1	36	36	36	4,617,421	ChIP	yes
Novershtern et al. 2011	human	HSPC-TAL1-rep1	0.69	1.31	0.18	-2	36	36	36	8,788,837	ChIP	yes
Novershtern et al. 2011	human	HSPC-TAL1-rep2	0.72	1.36	0.19	-2	36	36	36	7,439,145	ChIP	yes
Novershtern et al. 2011	human	HSPC-WCE	0.95	1.23	0.24	-2	36	36	36	6,321,189	Input	no
GSE23581	mouse	mES-Acitvin-Input	0.93	1.44	0.56	0	35	35	35	9,674,331	Input	no
GSE23581	mouse	mES-Acitvin-pSmad2	0.81	1.98	1.31	1	35	35	35	11,730,560	ChIP	yes
GSE23581	mouse	mES-DMSO-Input	0.93	1.51	0.69	0	35	35	35	10,750,428	Input	no
GSE23581	mouse	mES-DMSO-pSmad2	0.77	2.26	1.47	1	35	35	35	11,288,314	ChIP	no
GSE23581	mouse	mES-SP-Input	0.92	1.65	0.73	0	35	35	35	9,325,370	Input	no
GSE23581	mouse	mES-SP-pSmad2	0.8	2.16	1.08	1	35	35	35	9,079,108	ChIP	no
GSE26136	mouse	mES-Dpy-30	0.69	1.69	1.54	2	36	36	36	24,620,668	ChIP	yes
Klisch et al. 2011	mouse	Cerebella-Atoh1.control	0.95	2.28	0.97	0	35	35	35	10,310,101	Input	no
Klisch et al. 2011	mouse	Cerebella-Atoh1.rep1	0.89	7.47	1.86	2	35	35	35	2,649,698	ChIP	yes
Klisch et al. 2011	mouse	Cerebella-Atoh1.rep2	0.92	3.36	0.96	0	35	35	35	7,166,233	ChIP	yes
Klisch et al. 2011	mouse	Cerebella-IgG.s-5	0.69	2.33	1.44	1	36	36	36	8,514,915	IgG	no
Yang et al. 2011	mouse	WTTh17STAT3	0.73	4.58	1.63	2	25	25	25	28,501,100	ChIP	yes
Yang et al. 2011	mouse	WTTh17STAT5	0.58	6.08	1.65	2	25	25	25	30,799,471	ChIP	yes
Ebert et al. 2011; McManus et al. 2011	mouse	DP-Tcell-CTCF	0.35	5.94	5.08	2	36	36	36	13,326,337	ChIP	yes
Ebert et al. 2011; McManus et al. 2011	mouse	Mature-Bcell-CTCF	0.66	4.24	6.97	2	36	36	36	14,505,107	ChIP	yes
Ebert et al. 2011; McManus et al. 2011	mouse	Pro-Bcell-Rad21	0.84	6.82	3.35	2	36	36	36	25,074,201	ChIP	yes
Ebert et al. 2011; McManus et al. 2011	mouse	Pro-Bcell-Rag2KO-CTCF	0.62	4.52	3.98	2	36	36	36	15,641,228	ChIP	yes
Zhao et al. 2011	mouse	Myb-activated-B1T1	0.81	3.23	0.98	0	36	36	36	7,467,313	ChIP	yes
Zhao et al. 2011	mouse	Myb-activated-B1T2	0.83	3.33	0.88	0	36	36	36	5,454,019	ChIP	yes
Zhao et al. 2011	mouse	Myb-activated-B2	0.86	3.03	1.07	1	36	36	36	7,331,382	ChIP	yes
Zhao et al. 2011	mouse	Myb-activated-IgG	0.73	1.98	0.58	0	36	36	36	6,724,529	IgG	no
Zhao et al. 2011	mouse	Myb-inactivated-B1	0.83	1.79	0.5	-1	36	36	36	5,729,128	ChIP	no
Zhao et al. 2011	mouse	Myb-inactivated-B2	0.87	1.79	0.51	0	36	36	36	5,734,826	ChIP	no
Zhao et al. 2011	mouse	Myb-inactivated-IgG	0.94	4.16	0.34	-1	36	36	36	766,809	IgG	no
Rey et al. 2011	mouse	BMAL1-ZT02-rep1	0.8	2.4	1.15	1	37	37	37	9,023,818	ChIP	unknown
Rey et al. 2011	mouse	BMAL1-ZT02-rep2	0.74	1.76	1.5	1	37	37	37	24,294,126	ChIP	unknown
Rey et al. 2011	mouse	BMAL1-ZT06-rep1	0.66	2.1	1.71	2	37	37	37	20,808,528	ChIP	unknown
Rey et al. 2011	mouse	BMAL1-ZT06-rep2	0.25	5.49	1.89	2	37	37	37	20,234,777	ChIP	unknown
Rey et al. 2011	mouse	BMAL1-ZT10-rep1	0.9	1.75	0.92	0	37	37	37	9,220,495	ChIP	unknown
Rey et al. 2011	mouse	BMAL1-ZT10-rep2	0.84	1.74	1.53	2	37	37	37	22,892,744	ChIP	unknown
Rey et al. 2011	mouse	BMAL1-ZT14-rep1	0.88	1.61	0.84	0	37	37	37	12,447,404	ChIP	unknown
Rey et al. 2011	mouse	BMAL1-ZT14-rep2	0.64	2.91	2.58	2	37	37	37	20,961,930	ChIP	unknown
Rey et al. 2011	mouse	BMAL1-ZT18-rep1	0.7	2.03	1.77	2	37	37	37	22,079,073	ChIP	unknown
Rey et al. 2011	mouse	BMAL1-ZT18-rep2	0.21	4.51	1.94	2	37	37	37	30,803,372	ChIP	unknown
Rey et al. 2011	mouse	BMAL1-ZT22-rep1	0.67	2.77	1.33	1	37	37	37	10,194,650	ChIP	unknown

Continued on next page

Source	Species	Library	Complexity	NSC	RSC	QC	Ave. Read Length	Max. Read Length	Min. Read Length	Mapped reads	Type	Should exhibit read clustering
Rey et al. 2011	mouse	BMAL1-ZT22-rep2	0.88	1.4	0.85	0	37	37	37	21,473,568	ChIP	unknown
Rey et al. 2011	mouse	Input-DNA	0.84	1.76	1.44	1	37	37	37	21,940,254	Input	no
Koeppel et al. 2011	human	Saos-2-ChIP-Input-control	0.96	1.2	0.44	-1	35	35	35	15,967,510	Input	no
Koeppel et al. 2011	human	Saos-2-p53-replicate1-1	0.86	2.41	1	1	35	35	35	14,932,313	ChIP	yes
Koeppel et al. 2011	human	Saos-2-p53-replicate2	0.95	2.26	1.68	2	35	35	35	14,969,104	ChIP	yes
Koeppel et al. 2011	human	Saos-2-TAp73alpha-replicate1	0.84	2.57	2.6	2	35	35	35	14,905,593	ChIP	yes
Koeppel et al. 2011	human	Saos-2-TAp73alpha-replicate2	0.93	2.05	1.34	1	35	35	35	14,626,232	ChIP	yes
Koeppel et al. 2011	human	Saos-2-TAp73beta-replicate1	0.96	5.1	4.12	2	32	32	32	4,927,558	ChIP	yes
Koeppel et al. 2011	human	Saos-2-TAp73beta-replicate2	0.94	6.37	2.61	2	35	35	35	16,272,496	ChIP	yes
He et al. 2011	mouse	HL1-BirA-control-1	0.3	8.52	9.14	2	40	40	40	15,388,943	IgG	no
He et al. 2011	mouse	HL1-Gata4-1	0.57	2.11	2.92	2	37.77	40	35	21,352,298	ChIP	yes
He et al. 2011	mouse	HL1-Input-control	0.92	1.94	1.84	2	36	36	36	13,770,246	Input	no
He et al. 2011	mouse	HL1-Mef2a-1	0.92	1.34	1	1	38.18	40	35	20,160,274	ChIP	yes
He et al. 2011	mouse	HL1-Nkx2-5-1	0.91	1.69	3.09	2	38.59	40	36	24,181,076	ChIP	yes
He et al. 2011	mouse	HL1-P300	0.9	1.83	1.28	1	36	36	36	15,446,431	ChIP	yes
He et al. 2011	mouse	HL1-Srf-1	0.8	1.81	2.37	2	38.29	40	36	25,881,877	ChIP	yes
He et al. 2011	mouse	HL1-Tbx5-1	0.9	2.1	2.13	2	37.91	40	36	11,074,980	ChIP	yes
Bugge et al. 2011	mouse	Liver-HDAC3-ZT10	0.49	2.56	1.67	2	36.93	38	36	38,211,882	ChIP	unknown
Bugge et al. 2011	mouse	Liver-HDAC3-ZT22	0.76	1.34	1.09	1	39.03	40	38	38,822,996	ChIP	unknown
Bugge et al. 2011	mouse	Liver-input-Mnase-ZT10	0.81	4.86	1.96	2	38	38	38	20,627,184	Input	no
Bugge et al. 2011	mouse	Liver-input-Mnase-ZT22	0.8	4.49	1.52	2	38	38	38	18,828,586	Input	no
Bugge et al. 2011	mouse	Liver-input-ZT10	0.58	1.35	1.13	1	40	40	40	18,254,032	Input	no
Bugge et al. 2011	mouse	Liver-input-ZT22	0.64	2.1	2.06	2	40	40	40	14,072,057	Input	no
Bugge et al. 2011	mouse	Liver-NCoR-ZT10	0.72	2.5	1.24	1	38	38	38	10,955,647	ChIP	unknown
Bugge et al. 2011	mouse	Liver-NCoR-ZT22	0.8	1.4	0.86	0	38	38	38	18,218,400	ChIP	unknown
Bugge et al. 2011	mouse	Liver-Rev-erba-ZT10	0.54	3.5	1.52	2	36	36	36	23,266,910	ChIP	unknown
Bugge et al. 2011	mouse	Liver-Rev-erba-ZT22	0.38	1.8	0.87	0	36	36	36	26,701,376	ChIP	unknown
Siersbæk et al. 2011	mouse	CEBPbeta-2-hours	0.86	4.2	4.51	2	36	36	36	13,391,765	ChIP	yes
Siersbæk et al. 2011	mouse	CEBPbeta-4-hours	0.82	3.94	5.44	2	36	36	36	14,184,719	ChIP	yes
Siersbæk et al. 2011	mouse	CEBPbeta-day-0	0.69	4.74	5.95	2	36	36	36	13,823,228	ChIP	yes
Siersbæk et al. 2011	mouse	CEBPbeta-day-2	0.77	3.59	2.97	2	36	36	36	11,535,365	ChIP	yes
Siersbæk et al. 2011	mouse	CEBPdelta-4-hours	0.63	6.24	3.44	2	40	40	40	11,803,122	ChIP	yes
Siersbæk et al. 2011	mouse	CEBPdelta-day-0	0.59	5.05	4.73	2	40	40	40	12,036,027	ChIP	yes
Siersbæk et al. 2011	mouse	GR-4-hours	0.41	3.12	1.97	2	24	24	24	9,694,597	ChIP	yes
Siersbæk et al. 2011	mouse	Input	0.92	1.27	0.71	0	36	36	36	12,904,842	Input	no
Siersbæk et al. 2011	mouse	PPARgamma-day-2	0.29	2.31	0.96	0	40	40	40	13,429,961	ChIP	yes
Siersbæk et al. 2011	mouse	PPARgamma-day-6	0.3	2.28	1.64	2	40	40	40	14,620,856	ChIP	yes
Siersbæk et al. 2011	mouse	RXR-4-hours	0.7	3.12	2.85	2	40	40	40	12,219,467	ChIP	yes
Siersbæk et al. 2011	mouse	Stat5a-4-hours	0.62	4.33	5.62	2	36	36	36	13,644,334	ChIP	yes
Smeenk et al. 2011	human	U2OS-p53-ActD	0.97	5.72	1.81	2	32	32	32	6,940,755	ChIP	yes
Smeenk et al. 2011	human	U2OS-p53-Eto	0.97	4.28	1.27	1	32	32	32	7,272,634	ChIP	yes
Smeenk et al. 2011	human	U2OS-p53-pS15-ActD	0.96	1.74	0.54	0	32	32	32	4,742,221	ChIP	yes
Smeenk et al. 2011	human	U2OS-p53-pS15-Eto	0.94	1.8	0.66	0	32	32	32	6,590,995	ChIP	yes
Smeenk et al. 2011	human	U2OS-p53-pS46-ActD	0.92	1.63	0.29	-1	32	32	32	5,408,031	ChIP	yes
Smeenk et al. 2011	human	U2OS-p53-pS46-Eto	0.94	1.84	0.47	-1	32	32	32	5,748,594	ChIP	yes
Ceol et al. 2011	human	WM262-MCAF1	0.45	3.9	1.5	1	36	36	36	13,346,938	ChIP	yes
Ceol et al. 2011	human	WM262-SetDB1	0.54	1.49	0.62	0	36	36	36	5,307,748	ChIP	yes
Ceol et al. 2011	human	Wm451-lu-SetDB1	0.39	1.39	0.67	0	36	36	36	6,295,121	ChIP	yes
Wu et al. 2011a; Wu et al. 2011b	mouse	mES-IgG-exp1-no-KD	0.67	1.62	1.07	1	25	25	25	23,036,303	IgG	no
Wu et al. 2011a; Wu et al. 2011b	mouse	mES-IgG-exp2-mock-KD	0.52	2.04	1.4	1	36	36	36	8,283,677	IgG	no
Wu et al. 2011a; Wu et al. 2011b	mouse	mES-Tet1-exp1-no-KD	0.91	1.33	1.25	1	25	25	25	28,536,436	ChIP	yes
Wu et al. 2011a; Wu et al. 2011b	mouse	mES-Tet1-exp2-mock-KD	0.89	1.54	1	0	36	36	36	9,521,384	ChIP	yes
Wu et al. 2011a; Wu et al. 2011b	mouse	mES-Tet1-exp3-Tet1-KD	0.9	1.33	1.06	1	25	25	25	28,297,858	ChIP	no
Wu et al. 2011a; Wu et al. 2011b	mouse	mES-Tet1-exp4-Tet1-KD	0.56	1.71	0.5	0	25	25	25	4,286,921	ChIP	no
Horiuchi et al. 2011	mouse	Th1-1-Input	0.84	1.4	0.36	-1	36	36	36	5,981,126	Input	no
Horiuchi et al. 2011	mouse	Th1-2-Input	0.88	2.25	1.22	1	36	36	36	4,609,331	Input	no
Horiuchi et al. 2011	mouse	Th1-GATA3	0.94	1.81	0.62	0	36	36	36	7,219,267	ChIP	no
Horiuchi et al. 2011	mouse	Th1-IgG	0.78	2.37	1.02	1	36	36	36	11,136,690	IgG	no
Horiuchi et al. 2011	mouse	Th2-1-Input	0.81	1.58	0.42	-1	36	36	36	5,428,949	Input	no
Horiuchi et al. 2011	mouse	Th2-2-Input	0.88	2.28	1.33	1	36	36	36	4,095,787	Input	no
Horiuchi et al. 2011	mouse	Th2-GATA3	0.91	1.49	0.41	-1	36	36	36	6,332,390	ChIP	yes
Horiuchi et al. 2011	mouse	Th2-IgG	0.77	1.26	0.25	-2	36	36	36	11,532,524	IgG	no
Soccio et al. 2011	human	Human-Adipocytes-Input-rep1-GAII	0.38	1.43	0.56	0	38	38	38	23,331,240	Input	no
Soccio et al. 2011	human	Human-Adipocytes-PPARg-rep1-GAII	0.21	2.69	0.65	0	38	38	38	17,934,158	ChIP	yes

Continued on next page

Table S1 – *Continued from previous page*

Source	Species	Library	Complexity	NSC	RSC	QC	Ave. Read Length	Max. Read Length	Min. Read Length	Mapped reads	Type	Should exhibit read clustering
Soccio et al. 2011	human	Human-Adipocytes-PPARg-rep2-GAII	0.71	1.95	0.56	0	40	40	40	19,418,441	ChIP	yes
Soccio et al. 2011	human	Human-Liver-FOXA2-rep1-GAI-1	0.89	2.46	0.26	-1	32	32	32	3,597,158	ChIP	yes
Soccio et al. 2011	human	Human-Liver-FOXA2-rep1-GAII	0.87	1.84	0.29	-1	36	36	36	6,591,761	ChIP	yes
Soccio et al. 2011	human	Human-Liver-FOXA2-rep2-GAI-1	0.89	2.41	0.26	-1	32	32	32	3,559,308	ChIP	yes
Soccio et al. 2011	human	Human-Liver-FOXA2-rep2-GAII	0.8	2.73	0.44	-1	36	36	36	6,415,023	ChIP	yes
Soccio et al. 2011	human	Human-Liver-Input-rep1-GAI	0.98	1.57	0.13	-2	36	36	36	4,853,927	Input	no
Soccio et al. 2011	human	Human-Liver-Input-rep1-GAII	0.96	1.27	0.11	-2	32	32	32	2,775,576	Input	no
Soccio et al. 2011	human	Human-Liver-Input-rep2-GAI	0.96	1.49	0.11	-2	32	32	32	2,636,496	Input	no
Soccio et al. 2011	mouse	Mouse-Adipocytes-PPARg-rep2-GAII	0.68	1.69	0.91	0	36	36	36	16,907,011	ChIP	yes
Soccio et al. 2011	mouse	Mouse-Liver-FOXA2-rep1-GAI	0.87	3.47	0.35	-1	36	36	36	2,288,906	ChIP	yes
Soccio et al. 2011	mouse	Mouse-Liver-FOXA2-rep2-GAI	0.77	4.67	0.6	0	36	36	36	2,986,172	ChIP	yes
Soccio et al. 2011	mouse	Mouse-Liver-FOXA2-rep3-GAI	0.56	3.79	0.46	-1	36	36	36	7,770,167	ChIP	yes
Soccio et al. 2011	mouse	Mouse-Liver-FOXA2-rep3-GAII	0.81	3.35	0.83	0	36	36	36	2,686,815	ChIP	yes
Soccio et al. 2011	mouse	Mouse-Liver-FOXA2-rep4-GAI	0.46	3.39	0.32	-1	36	36	36	7,311,631	ChIP	yes
Soccio et al. 2011	mouse	Mouse-Liver-FOXA2-rep4-GAII	0.84	5.04	1.06	1	36	36	36	1,701,117	ChIP	yes
Soccio et al. 2011	mouse	Mouse-Liver-Input-rep1-GAI	0.91	2.15	0.32	-1	36	36	36	3,658,469	Input	no
Soccio et al. 2011	mouse	Mouse-Liver-Input-rep2-GAI	0.91	2.34	0.4	-1	36	36	36	3,808,896	Input	no
Soccio et al. 2011	mouse	Mouse-Liver-Input-rep3-GAI	0.89	2.36	0.4	-1	36	36	36	3,849,533	Input	no
Ang et al. 2011	mouse	CCE-mES-Input	0.94	1.18	0.37	-1	36	36	36	20,085,978	Input	no
Ang et al. 2011	mouse	CCE-mES-Negative	0.78	4.01	1.06	1	36	36	36	5,894,488	Input	no
Ang et al. 2011	mouse	CCE-mES-Oct4	0.97	1.58	0.55	0	36	36	36	4,368,039	ChIP	yes
Ang et al. 2011	mouse	CCE-mES-Rbbp5	0.85	1.1	0.14	-2	36	36	36	20,687,485	ChIP	yes
Ang et al. 2011	mouse	CCE-mES-Wdr5	0.39	2.95	1.05	1	36	36	36	18,192,088	ChIP	yes
Ang et al. 2011	mouse	CCE-mES-WDR5-FL	0.93	2.09	0.89	0	36	36	36	9,435,450	ChIP	yes
Verzi et al. 2011	mouse	Jejunum-villus-cells-Cdx2	0.87	3.18	1.4	1	40	40	40	5,335,016	ChIP	yes
Verzi et al. 2011	mouse	Jejunum-villus-cells-Input	0.91	1.92	0.76	0	40	40	40	5,579,736	Input	no
Wang et al. 2011	human	LNCaP-AR-dht-siCTRL	0.94	1.31	0.3	-1	25	25	25	12,537,593	ChIP	yes
Wang et al. 2011	human	LNCaP-AR-dht-siFoxA1	0.96	2.25	1.64	2	25	25	25	7,690,074	ChIP	unknown
Wang et al. 2011	human	LNCaP-FoxA1-dht-siCTRL	0.95	5.46	5.19	2	22	22	22	7,796,027	ChIP	yes
Wang et al. 2011	human	LNCaP-FoxA1-vehicle-siCTRL	0.95	3.88	3	2	22	22	22	7,780,805	ChIP	yes
Wang et al. 2011	human	LNCaP-input-dht-1	0.98	1.48	0.58	0	36	36	36	4,211,736	Input	no
Wang et al. 2011	human	LNCaP-MED12-dht-siCTRL	0.98	1.53	0.45	-1	36	36	36	4,305,257	ChIP	yes
Wang et al. 2011	human	LNCaP-MED12-dht-siFoxA1	0.96	1.55	0.46	-1	28	28	28	17,506,375	ChIP	yes
Wang et al. 2011	human	LNCaP-p300-dht-siCTRL	0.98	1.59	0.51	0	36	36	36	3,133,925	ChIP	yes
Wang et al. 2011	human	LNCaP-p300-dht-siFoxA1	0.98	1.62	0.53	0	36	36	36	3,120,380	ChIP	yes
Nitzsche et al. 2011	mouse	mESC-CTCF-GFP	0.95	2.74	2.78	2	35	35	35	9,433,929	ChIP	yes
Nitzsche et al. 2011	mouse	mESC-IgG	0.94	1.92	0.8	0	35	35	35	9,008,251	IgG	no
Nitzsche et al. 2011	mouse	EB-Rad21-GFP	0.93	2.51	1.93	2	35	35	35	9,039,705	ChIP	yes
Nitzsche et al. 2011	mouse	EB-Rad21-GFP-IgG	0.91	2.25	1.19	1	35	35	35	8,488,336	IgG	no
Nitzsche et al. 2011	mouse	mESC-Rad21-GFP	0.92	2.38	2.86	2	35	35	35	20,118,696	ChIP	yes
Nitzsche et al. 2011	mouse	mESC-Rad21-GFP-IgG	0.91	2.12	1.86	2	35	35	35	18,171,398	IgG	no
Kim et al. 2011	human	Endoderm-FOXH1-pool	0.95	6.88	1.72	2	36	36	36	11,630,871	ChIP	yes
Kim et al. 2011	human	Endoderm-Input	0.97	1.45	0.69	0	36	36	36	16,775,681	Input	no
Kim et al. 2011	human	Endoderm-SMAD2-3-A-pool	0.98	1.69	0.71	0	36	36	36	10,591,855	ChIP	yes
Kim et al. 2011	human	Endoderm-SMAD2-3-B-rep1	0.98	2.65	1.02	1	36	36	36	6,467,438	ChIP	yes
Kim et al. 2011	human	Endoderm-SMAD3-rep1	0.98	1.86	0.63	0	36	36	36	6,664,422	ChIP	yes
Kim et al. 2011	human	Endoderm-SMAD4-rep1	0.98	2.4	0.98	0	36	36	36	6,664,039	ChIP	yes
Kim et al. 2011	human	hESC-FOXH1-pool-1	0.97	3.21	1.37	1	36	36	36	11,570,426	ChIP	yes
Kim et al. 2011	human	hESC-Input-1	0.65	1.53	0.51	0	36	36	36	30,699,298	Input	no
Kim et al. 2011	human	hESC-SMAD2-3-A-pool	0.98	1.88	0.9	0	36	36	36	11,364,210	ChIP	yes
Kim et al. 2011	human	hESC-SMAD2-3-B-rep1	0.97	2.3	0.93	0	36	36	36	9,667,298	ChIP	yes
Kim et al. 2011	human	hESC-SMAD3-rep1	0.98	1.78	0.81	0	36	36	36	7,743,314	ChIP	yes
Kim et al. 2011	human	hESC-SMAD4-rep1	0.96	1.89	0.75	0	36	36	36	10,007,703	ChIP	yes
Lo et al. 2011	human	Adipocytes-CEBPa	0.4	15.56	0.55	0	35	35	35	1,285,131	ChIP	yes
Lo et al. 2011	human	Adipocytes-E2F4	0.89	13.68	0.2	-2	35	35	35	64,667	ChIP	yes
Lo et al. 2011	human	Adipocytes-HSF1	0.74	6.03	0.05	-2	35	35	35	177,695	ChIP	yes
Lo et al. 2011	human	Adipocytes-IgG	0.71	21.81	0.12	-2	35	35	35	282,753	IgG	no
Tijssen et al. 2011	human	Megakaryocytes-FLI1	0.95	2.17	0.9	0	54	54	54	12,154,848	ChIP	yes
Tijssen et al. 2011	human	Megakaryocytes-GATA1	0.92	2.75	1.05	1	37	37	37	12,848,211	ChIP	yes
Tijssen et al. 2011	human	Megakaryocytes-GATA2	0.95	2.3	0.83	0	54	54	54	8,984,141	ChIP	yes
Tijssen et al. 2011	human	Megakaryocytes-rIgG	0.68	2.11	0.89	0	37	37	37	13,241,658	IgG	no
Tijssen et al. 2011	human	Megakaryocytes-RUNX1	0.97	8.42	2.6	2	54	54	54	10,822,021	ChIP	yes
Tijssen et al. 2011	human	Megakaryocytes-SCL	0.96	1.34	0.26	-1	54	54	54	11,782,604	ChIP	yes
Tan et al. 2011	human	MCF7-E2-AP2g	0.94	3.36	1.94	2	36	36	36	13,328,869	ChIP	yes
Tan et al. 2011	human	MCF7-E2-FoxA1	0.93	6.24	2.32	2	36	36	36	14,308,936	ChIP	yes
Tan et al. 2011	human	MCF7-EtOH-AP2g	0.95	3.31	2.03	2	36	36	36	13,306,339	ChIP	yes
Tan et al. 2011	human	MCF7-EtOH-FoxA1	0.92	6.76	2.13	2	36	36	36	17,586,631	ChIP	yes
Handoko et al. 2011	mouse	E14-mES-CTCF	0.8	26.13	2.04	2	37	37	37	14,006,006	ChIP	yes
Handoko et al. 2011	mouse	E14-mES-Input	0.96	1.17	0.18	-2	37	37	37	9,567,449	Input	no

Continued on next page

Table S1 – *Continued from previous page*

Source	Species	Library	Complexity	NSC	RSC	QC	Ave. Read Length	Max. Read Length	Min. Read Length	Mapped reads	Type	Should exhibit read clustering
Handoko et al. 2011	mouse	E14-mES-LaminB	0.89	1.45	0.8	0	36	36	36	15,336,482	ChIP	yes
Handoko et al. 2011	mouse	E14-mES-p300	0.96	1.35	0.73	0	37	37	37	17,677,307	ChIP	yes
Hu et al. 2011	human	CD34-WT-Brg1-1	0.9	2	0.77	0	25	25	25	6,821,309	ChIP	yes
Hu et al. 2011	human	CD34-WT-CTCF	0.93	2.76	0.96	0	25	25	25	6,413,538	ChIP	yes
Hu et al. 2011	human	CD34-WT-input	0.94	1.85	0.44	-1	25	25	25	3,838,343	Input	no
Hu et al. 2011	human	CD34-WT-TAL1	0.87	1.69	0.23	-2	25	25	25	3,089,084	ChIP	yes
Hu et al. 2011	human	CD36-shBrg1-CTCF	0.9	12.03	2.57	2	24.63	25	24	10,427,559	ChIP	yes
Hu et al. 2011	human	CD36-shbrg1-GATA1	0.92	7.21	1.78	2	32.67	35	25	10,380,913	ChIP	yes
Hu et al. 2011	human	CD36-shBrg1-input	0.8	1.34	0.46	-1	25	25	25	8,880,654	Input	no
Hu et al. 2011	human	CD36-shbrg1-TAL1	0.97	10.44	1.8	2	25	25	25	10,119,729	ChIP	yes
Hu et al. 2011	human	CD36-shLuc-CTCF	0.81	12.76	2.53	2	25	25	25	9,434,898	ChIP	yes
Hu et al. 2011	human	CD36-shLuc-GATA1	0.89	6.8	1.84	2	25	25	25	13,602,919	ChIP	yes
Hu et al. 2011	human	CD36-shLuc-input	0.62	1.36	0.46	-1	25	25	25	10,984,175	Input	no
Hu et al. 2011	human	CD36-shLuc-TAL1	0.97	12.67	2	2	25	25	25	10,455,880	ChIP	yes
Hu et al. 2011	human	CD36-WT-Brg1-1	0.85	1.66	0.56	0	25	25	25	13,673,639	ChIP	yes
Hu et al. 2011	human	CD36-WT-input-1	0.8	2.53	1.05	1	25	25	25	10,309,351	Input	no
Zhao et al. 2011	human	IB4-EBNA2-rep1	0.94	1.91	0.3	-1	36	36	36	5,803,658	ChIP	yes
Zhao et al. 2011	human	IB4-EBNA2-rep2	0.95	2.87	1.03	1	40	40	40	5,536,068	ChIP	yes
Zhao et al. 2011	human	IB4-Input-rep1	0.98	1.38	0.3	-1	40	40	40	4,144,311	Input	no
Zhao et al. 2011	human	IB4-Input-rep2	0.96	1.27	0.3	-1	40	40	40	10,404,527	Input	no
Zhao et al. 2011	human	IB4-RBPJ-rep1	0.98	2.48	0.6	0	36	36	36	2,919,539	ChIP	yes
Zhao et al. 2011	human	IB4-RBPJ-rep2	0.93	3.03	1.26	1	40	40	40	7,475,552	ChIP	yes
Rao et al. 2011	human	HeLaB2-GR-DMSO-GRKD	0.39	8.06	6.32	2	35	35	35	25,313,813	ChIP	no
Rao et al. 2011	human	HeLaB2-GR-DMSO-p65KD	0.96	4.84	4.97	2	35	35	35	12,286,932	ChIP	unknown
Rao et al. 2011	human	HeLaB2-GR-DMSO-WT	0.6	1.35	0.51	0	35	35	35	26,883,356	ChIP	unknown
Rao et al. 2011	human	HeLaB2-GR-TA-WT	0.95	1.82	1.14	1	35	35	35	13,061,670	ChIP	unknown
Rao et al. 2011	human	HeLaB2-GR-TA+TNFa-GRKD	0.55	3.84	3.05	2	35	35	35	23,851,932	ChIP	unknown
Rao et al. 2011	human	HeLaB2-GR-TA+TNFa-p65KD	0.96	5.27	2.96	2	35	35	35	13,570,984	ChIP	unknown
Rao et al. 2011	human	HeLaB2-GR-TA+TNFa-WT	0.66	1.63	0.99	0	35	35	35	27,313,718	ChIP	unknown
Rao et al. 2011	human	HeLaB2-GR-TNFa-WT	0.96	1.29	0.47	-1	35	35	35	13,022,367	ChIP	unknown
Rao et al. 2011	human	HeLaB2-p65-DMSO-GRKD	0.4	7.86	7.04	2	35	35	35	25,556,594	ChIP	no
Rao et al. 2011	human	HeLaB2-p65-DMSO-p65KD	0.92	5.12	3.33	2	35	35	35	15,380,858	ChIP	no
Rao et al. 2011	human	HeLaB2-p65-DMSO-WT	0.52	1.73	0.99	0	35	35	35	17,693,337	ChIP	no
Rao et al. 2011	human	HeLaB2-p65-TA-WT	0.93	1.66	1.03	1	35	35	35	16,120,222	ChIP	yes
Rao et al. 2011	human	HeLaB2-p65-TA+TNFa-GRKD	0.58	4.86	4.49	2	35	35	35	25,972,505	ChIP	yes
Rao et al. 2011	human	HeLaB2-p65-TA+TNFa-p65KD	0.93	3.97	4.16	2	35	35	35	16,624,445	ChIP	no
Rao et al. 2011	human	HeLaB2-p65-TA+TNFa-WT	0.67	2.29	1.67	2	35	35	35	26,290,176	ChIP	yes
Rao et al. 2011	human	HeLaB2-p65-TNFa-WT	0.93	2.31	1.83	2	35	35	35	16,380,803	ChIP	yes
Wang et al. 2011	human	CUTLL-Input-1	0.98	1.24	0.42	-1	40	40	40	19,896,199	Input	no
Wang et al. 2011	human	CUTLL-Input-2	0.98	1.29	0.51	0	40	40	40	20,712,816	Input	no
Wang et al. 2011	human	CUTLL-Notch1-1	0.97	2.44	1.03	1	40	40	40	19,820,660	ChIP	yes
Wang et al. 2011	human	CUTLL-Notch1-2	0.93	4.98	1.27	1	40	40	40	15,252,998	ChIP	yes
Wang et al. 2011	human	CUTLL-RBPJ-1	0.97	1.57	0.69	0	40	40	40	20,226,038	ChIP	yes
Wang et al. 2011	human	CUTLL-RBPJ-2	0.9	3.1	1.06	1	40	40	40	17,569,147	ChIP	yes
Wang et al. 2011	human	CUTLL-ZNF143	0.8	7.23	1.9	2	40	40	40	25,444,869	ChIP	yes
Wang et al. 2011	mouse	G4A2-Input	0.6	1.99	1.14	1	39	39	39	21,212,246	Input	no
Wang et al. 2011	mouse	G4A2-Notch1	0.73	2.24	1.62	2	39	39	39	27,613,376	ChIP	yes
Wang et al. 2011	mouse	G4A2-RBPJ	0.89	1.53	1.03	1	40	40	40	12,929,417	ChIP	yes
Wang et al. 2011	mouse	T6E-Input	0.96	1.24	0.76	0	38	38	38	24,179,307	Input	no
Wang et al. 2011	mouse	T6E-Notch1	0.92	2.22	1.15	1	38	38	38	21,336,323	ChIP	yes
Wang et al. 2011	mouse	T6E-RBPJ	0.93	1.74	0.87	0	38	38	38	16,046,706	ChIP	yes
Costessi et al. 2011	human	K562-NFYA	0.78	4.8	6.12	2	35	35	35	11,661,523	ChIP	yes
Costessi et al. 2011	human	K562-NFYB	0.58	5.4	6.13	2	35	35	35	15,460,623	ChIP	yes
Costessi et al. 2011	human	K562-PRAME	0.87	1.76	1.32	1	35	35	35	6,685,161	ChIP	yes
Costessi et al. 2011	human	K562-Preimmune	0.9	1.67	0.84	0	35	35	35	6,366,475	IgG	no
Miyazaki et al. 2011	mouse	E2A-Day0	0.94	1.39	0.26	-1	36	36	36	9,650,009	ChIP	yes
Miyazaki et al. 2011	mouse	E2A-Day2	0.94	1.34	0.19	-2	36	36	36	8,529,512	ChIP	unknown
Miyazaki et al. 2011	mouse	Input	0.89	1.4	0.24	-2	36	36	36	11,673,268	Input	no
GSE26711	mouse	C2C12-FLAG	0.95	1.76	0.19	-2	26	26	26	2,144,135	IgG	no
GSE26711	mouse	C2C12-FLAG-Msx1	0.74	2.6	1.35	1	32.58	26	36	4,769,291	ChIP	yes
Sun et al. 2011	mouse	MEF-Input	0.85	1.37	0.8	0	36	36	36	17,709,015	Input	no
Sun et al. 2011	mouse	MEF-NelfB	0.75	1.99	1.52	2	36	36	36	16,971,968	ChIP	yes
Heikkinen et al. 2011	human	THP1-calcitriol-VDR	0.41	1.63	0.58	0	36	36	36	26,125,837	ChIP	yes
Heikkinen et al. 2011	human	THP1-IgG	0.46	1.4	0.28	-1	36	36	36	26,578,895	IgG	no
Heikkinen et al. 2011	human	THP1-unstimulated-VDR	0.4	1.37	0.32	-1	36	36	36	22,822,851	ChIP	no
Yoon et al. 2011	xaenopus	Input	0.59	N/A	N/A	N/A	36	36	36	3,219,500	Input	no
Yoon et al. 2011	xaenopus	Smad2-3	0.93	N/A	N/A	N/A	36	36	36	8,168,342	ChIP	yes
Mullen et al. 2011	human	BGO3-Oct4	0.94	2.13	1.51	2	36	36	36	7,835,807	ChIP	yes
Mullen et al. 2011	human	BGO3-Smad3	0.9	2.21	0.73	0	36	36	36	10,206,400	ChIP	yes
Mullen et al. 2011	human	BGO3-WCE	0.99	1.41	0.55	0	36	36	36	8,589,186	Input	no
Mullen et al. 2011	mouse	ESC-Activin-Smad3	0.85	1.98	0.36	-1	36	36	36	3,469,014	ChIP	yes

Continued on next page

Table S1 – *Continued from previous page*

Source	Species	Library	Complexity	NSC	RSC	QC	Ave. Read Length	Max. Read Length	Min. Read Length	Mapped reads	Type	Should exhibit read clustering
Mullen et al. 2011	mouse	ESC-Smad2-3-Activin	0.85	1.97	0.35	-1	36	36	36	3,521,351	ChIP	yes
Mullen et al. 2011	mouse	ESC-Smad3	0.92	1.86	0.3	-1	26	26	26	3,650,000	ChIP	unknown
Mullen et al. 2011	mouse	mESC-NoMyod1-Day2-Smad3	0.84	1.67	1.74	2	36	36	36	8,780,818	ChIP	yes
Mullen et al. 2011	mouse	mESC-NoMyod1-Day5-Smad3	0.64	2.84	1.43	1	36	36	36	7,935,259	ChIP	yes
Mullen et al. 2011	mouse	mESC-PlusMyod1-Day2-Smad3	0.71	1.89	1.48	1	36	36	36	13,783,301	ChIP	yes
Mullen et al. 2011	mouse	mESC-PlusMyod1-Day5-MyoD-H2Flag	0.67	2.13	1.19	1	36	36	36	12,790,865	ChIP	yes
Mullen et al. 2011	mouse	mESC-PlusMyod1-Day5-Smad3	0.75	1.73	1	0	36	36	36	8,585,103	ChIP	yes
Mullen et al. 2011	mouse	Myotubes-IgG	0.65	1.98	0.75	0	36	36	36	5,056,829	IgG	no
Mullen et al. 2011	mouse	Myotubes-MyoD1-Rep1	0.78	6.25	2.23	2	36	36	36	4,485,416	ChIP	yes
Mullen et al. 2011	mouse	Myotubes-MyoD1-Rep2	0.54	3.75	1.62	2	36	36	36	14,493,250	ChIP	yes
Mullen et al. 2011	mouse	Myotubes-Smad3-Rep1	0.68	3.39	2.15	2	36	36	36	14,630,938	ChIP	yes
Mullen et al. 2011	mouse	Myotubes-Smad3-Rep2	0.19	2.5	2.03	2	36	36	36	11,953,645	ChIP	yes
Mullen et al. 2011	mouse	Pro-Bcells-IgG	0.76	3.51	1.09	1	36	36	36	22,066,974	IgG	no
Mullen et al. 2011	mouse	Pro-Bcells-PU.1-Rep1	0.61	4	1.75	2	36	36	36	11,557,346	ChIP	yes
Mullen et al. 2011	mouse	Pro-Bcells-PU.1-Rep2	0.76	6.98	1.47	1	36	36	36	21,066,565	ChIP	yes
Mullen et al. 2011	mouse	Pro-Bcells-Smad3-Rep1	0.68	1.84	1.26	1	36	36	36	13,801,014	ChIP	yes
Mullen et al. 2011	mouse	Pro-Bcells-Smad3-Rep2	0.74	4.61	2.45	2	36	36	36	13,745,867	ChIP	yes
Wei et al. 2011	mouse	CD4-Gata3	0.5	3.55	1.68	2	25	26	25	5,311,260	ChIP	yes
Wei et al. 2011	mouse	CD8-Fli1	0.64	1.78	0.91	0	25	25	25	4,267,162	ChIP	unknown
Wei et al. 2011	mouse	CD8-Gata3	0.8	1.9	1.08	1	25	25	25	4,827,087	ChIP	yes
Wei et al. 2011	mouse	CD8-Gata3-KO-Fli1	0.95	1.49	0.69	0	25	25	25	3,152,001	ChIP	unknown
Wei et al. 2011	mouse	CD8-Gata3-KO-Gata3	0.92	1.32	0.4	-1	25	25	25	1,997,286	ChIP	yes
Wei et al. 2011	mouse	DN-Gata3	0.66	2.87	1.68	2	25	25	25	6,301,966	ChIP	yes
Wei et al. 2011	mouse	DP-Gata3	0.76	1.86	1.14	1	25	25	25	6,402,211	ChIP	yes
Wei et al. 2011	mouse	DP-Gata3-replicate	0.08	14.33	7.09	2	25	25	25	20,563,880	ChIP	yes
Wei et al. 2011	mouse	iTreg-Gata3	0.26	2.42	1.28	1	25	25	25	7,299,209	ChIP	yes
Wei et al. 2011	mouse	NKT-Gata3	0.21	16.16	2.81	2	25	25	25	4,716,486	ChIP	yes
Wei et al. 2011	mouse	nTreg-Gata3	0.69	5.83	2.01	2	25	25	25	4,163,536	ChIP	yes
Wei et al. 2011	mouse	Th17-Gata3	0.32	1.64	0.68	0	25	25	25	5,051,835	ChIP	unknown
Wei et al. 2011	mouse	Th1-Gata3	0.67	2.79	1.53	2	25	25	25	6,296,541	ChIP	yes
Wei et al. 2011	mouse	Th2-Ets1	0.37	3.54	1.87	2	25	25	25	1,620,989	ChIP	yes
Wei et al. 2011	mouse	Th2-Fli1	0.81	4.76	0.02	-2	24	24	24	444,327	ChIP	yes
Wei et al. 2011	mouse	Th2-Gata3	0.86	2.86	2.47	2	25	25	25	7,514,211	ChIP	yes
Wei et al. 2011	mouse	Th2-Gata3-replicate	0.86	2.86	2.47	2	25	25	25	7,514,211	ChIP	yes
Liu et al. 2011	mouse	mES-TAF1	0.7	1.04	0.11	-2	36	36	36	42,959,794	ChIP	yes
Liu et al. 2011	mouse	mES-TAF1-IgG	0.66	1.1	0.27	-1	36	36	36	38,486,238	IgG	no
Liu et al. 2011	mouse	mES-TAF3	0.48	1.76	0.94	0	36	36	36	37,109,895	ChIP	yes
Liu et al. 2011	mouse	mES-TAF3-IgG	0.38	1.11	0.2	-2	36	36	36	41,265,618	IgG	no
Liu et al. 2011	mouse	mES-TBP	0.64	2.1	0.93	0	36	36	36	34,110,153	ChIP	yes
Liu et al. 2011	mouse	mES-TBP-IgG	0.31	1.21	0.17	-2	36	36	36	33,960,211	IgG	no
Kong et al. 2011	human	MCF7-DMSO-GATA3	0.92	2.27	1.02	1	36	36	36	16,110,797	ChIP	yes
Kong et al. 2011	human	MCF7-DMSO-p300	0.94	1.49	0.53	0	36	36	36	16,598,044	ChIP	yes
Kong et al. 2011	human	MCF7-E2-GATA3	0.94	3.42	1.43	1	36	36	36	22,771,157	ChIP	yes
Kong et al. 2011	human	MCF7-E2-p300	0.92	1.54	0.46	-1	36	36	36	12,820,747	ChIP	yes
GSE31951	mouse	0hrKCl-Input-sampleB1	0.87	2.26	0.91	0	33	33	33	21,405,879	Input	no
GSE31951	mouse	0hrKCl-Input-sampleB2	0.61	1.21	0.17	-2	33	33	33	11,303,008	Input	no
GSE31951	mouse	0hrKCl-MeCP2IP-sampleB1	0.84	3.88	2.11	2	33	33	33	34,260,253	ChIP	yes
GSE31951	mouse	0hrKCl-MeCP2IP-sampleB2	0.8	1.97	1.68	2	33	33	33	14,827,886	ChIP	yes
GSE31951	mouse	2hrKcl-Input-sampleB1	0.88	1.97	0.32	-1	33	33	33	8,725,472	Input	no
GSE31951	mouse	2hrKCl-Input-sampleB2	0.77	1.15	0.16	-2	33	33	33	45,501,766	Input	no
GSE31951	mouse	2hrKCl-MeCP2IP-sampleB1	0.8	4.79	2.65	2	33	33	33	13,050,973	ChIP	yes
GSE31951	mouse	2hrKCl-MeCP2IP-sampleB2	0.47	1.9	1.33	1	33	33	33	9,573,633	ChIP	yes
GSE31951	mouse	2hrKCl-pS421MeCP2IP-sampleB2	0.89	1.86	0.67	0	33	33	33	3,733,245	ChIP	yes
Norton et al. 2011	rat	H4IIE-input	0.75	3.28	1.41	1	40	40	40	22,889,534	Input	no
Norton et al. 2011	rat	H4IIE-TCF7L2	0.81	3.08	1.14	1	40	40	40	21,999,570	ChIP	yes
Bernt et al. 2011	mouse	MLL-AF9	0.76	2.2	2.05	2	36	36	36	20,979,495	ChIP	yes
Sahu et al. 2011	human	LNCaP-AR-rep1	0.96	2.15	0.84	0	30	30	30	13,178,048	ChIP	yes
Sahu et al. 2011	human	LNCaP-AR-rep2	0.97	1.71	0.69	0	30	30	30	13,295,369	ChIP	yes
Sahu et al. 2011	human	LNCaP-AR-siFoxA1-rep1	0.97	2.45	1.13	1	30	30	30	16,070,383	ChIP	yes
Sahu et al. 2011	human	LNCaP-AR-siFoxA1-rep2	0.96	2.64	1.18	1	30	30	30	16,077,043	ChIP	yes
Sahu et al. 2011	human	LNCaP-FoxA1-rep1	0.98	3.05	1.14	1	30	30	30	7,592,193	ChIP	yes
Sahu et al. 2011	human	LNCaP-FoxA1-rep2	0.98	3.56	1.38	1	30	30	30	8,058,879	ChIP	yes
Sahu et al. 2011	human	LNCaP-FoxA1-siFoxA1-rep1	0.97	1.77	0.44	-1	30	30	30	5,946,745	ChIP	no
Sahu et al. 2011	human	LNCaP-FoxA1-siFoxA1-rep2	0.97	1.76	0.42	-1	30	30	30	5,835,884	ChIP	no
Sahu et al. 2011	human	LNCaP-GR	0.97	1.78	0.83	0	36	36	36	22,124,446	ChIP	yes
Sahu et al. 2011	human	LNCaP-GR-siFoxA1	0.93	1.68	0.91	0	36	36	36	17,943,158	ChIP	yes
Sahu et al. 2011	human	LNCaP-rIgG	0.95	1.16	0.21	-2	30	30	30	16,327,209	IgG	no
An et al. 2011	mouse	C2C12-Input-rep1	0.95	1.13	0.48	-1	37	37	37	17,130,843	Input	no
An et al. 2011	mouse	C2C12-Input-rep2	0.94	1.1	0.5	-1	40	40	40	24,457,563	Input	no
An et al. 2011	mouse	C2C12-Sox6-rep1	0.92	2.16	0.39	-1	40	40	40	2,989,595	ChIP	yes

Continued on next page

Table S1 – *Continued from previous page*

Source	Species	Library	Complexity	NSC	RSC	QC	Ave. Read Length	Max. Read Length	Min. Read Length	Mapped reads	Type	Should exhibit read clustering
An et al. 2011	mouse	C2C12-Sox6-rep2	0.96	2.37	0.26	-1	40	40	40	1,470,144	ChIP	yes
Shukla et al. 2011	human	BJAB-CTCF	0.85	3.53	1.88	2	35	35	35	20,488,614	ChIP	yes
Shukla et al. 2011	human	BJAB-Rabbit-IgG	0.82	3.24	2.1	2	35	35	35	17,746,364	IgG	no
Shukla et al. 2011	human	BL41-CTCF	0.81	3.63	2.27	2	35	35	35	27,623,415	ChIP	yes
Shukla et al. 2011	human	BL41-Rabbit-IgG	0.68	4.44	3.71	2	35	35	35	29,655,822	IgG	no
Trompouki et al. 2011*	mouse	Gata1-G1ERbmp-r1-100914-4	0.97	1.5	0.99	0	36	36	36	18,435,160	ChIP	yes
Trompouki et al. 2011*	mouse	Gata2-G1Ebmp-r1-101201-3	0.82	12.68	1.59	2	36	36	36	8,484,282	ChIP	yes
Trompouki et al. 2011*	mouse	Smad1-G1Ebmp-r1-100914-6	0.8	4.78	1.52	2	36	36	36	14,561,496	ChIP	yes
Trompouki et al. 2011*	mouse	Smad1-G1ERbmp-r1-100914-5	0.85	4.16	1.38	1	36	36	36	16,186,687	ChIP	yes
Trompouki et al. 2011*	mouse	WCE-G1Ebmp-r1-101201-2	0.97	1.43	0.92	0	36	36	36	14,429,966	Input	no
Trompouki et al. 2011*	mouse	WCE-G1ERbio-r1-100914-1	0.97	1.53	0.98	0	36	36	36	17,835,267	Input	no
Trompouki et al. 2011*	human	GATA1-CD34eryth-bio-r1-101103-6	0.73	4.25	0.05	-2	36	36	36	94,232	ChIP	yes
Trompouki et al. 2011*	human	GATA1-CD34eryth-bio-r2-101103-7	0.22	21.87	0.14	-2	36	36	36	744,924	ChIP	yes
Trompouki et al. 2011*	human	GATA1-CD34eryth-bmp-r1-100922-4	0.57	10.86	0.14	-2	36	36	36	667,864	ChIP	yes
Trompouki et al. 2011*	human	GATA1-CD34eryth-bmp-r2-101105-1	0.49	10.81	0.15	-2	36	36	36	900,792	ChIP	yes
Trompouki et al. 2011*	human	GATA2-CD34prog-bmp-r1-101201-1	0.59	2.91	0.05	-2	36	36	36	479,725	ChIP	yes
Trompouki et al. 2011*	human	SMAD1-CD34eryth-bmp-r1-100922-5	0.65	6.9	0.07	-2	36	36	36	634,638	ChIP	yes
Trompouki et al. 2011*	human	SMAD1-CD34eryth-bmp-r2-101103-7	0.62	9.05	0.07	-2	36	36	36	730,479	ChIP	yes
Trompouki et al. 2011*	human	SMAD1-CD34prog-bmp-r1-100901-1	0.68	4.26	0.06	-2	36	36	36	322,324	ChIP	yes
Trompouki et al. 2011*	human	SMAD1-CD34prog-bmp-r2-101105-3	0.68	3.35	0.06	-2	36	36	36	620,959	ChIP	yes
Trompouki et al. 2011*	human	TCF7L2-CD34prog-bio-r1-100826-7	0.72	3.01	0.06	-2	36	36	36	339,258	ChIP	yes
Trompouki et al. 2011*	human	TCF7L2-CD34prog-bio-r2-101105-4	0.64	2.96	0.07	-2	36	36	36	529,093	ChIP	yes
Trompouki et al. 2011*	human	WCE-CD34eryth-bio-r1-101103-4	0.59	19.41	0.28	-1	36	36	36	79,783	Input	no
Trompouki et al. 2011*	human	WCE-CD34eryth-bio-r1-101201-4	0.54	3.54	0.05	-2	36	36	36	502,346	Input	no
Trompouki et al. 2011*	human	WCE-CD34eryth-bio-r2-101103-5	0.69	8.49	0.04	-2	36	36	36	340,786	Input	no
Trompouki et al. 2011*	human	WCE-CD34eryth-bmp-r1-100922-3	0.69	11.24	0.05	-2	36	36	36	246,281	Input	no
Trompouki et al. 2011*	human	WCE-CD34eryth-bmp-r2-101105-2	0.68	10.7	0.05	-2	36	36	36	300,293	Input	no
Trompouki et al. 2011*	human	WCE-CD34prog-bio-r1-100826-6	0.65	2.88	0.04	-2	36	36	36	356,819	Input	no
Trompouki et al. 2011*	human	WCE-CD34prog-bio-r1-101201-1	0.59	2.91	0.05	-2	36	36	36	479,725	Input	no
Trompouki et al. 2011*	human	WCE-CD34prog-bio-r2-101105-5	0.65	2.75	0.04	-2	36	36	36	283,167	Input	no
Trompouki et al. 2011*	human	WCE-CD34prog-bmp-r1-101201-7	0.6	3.13	0.05	-2	36	36	36	430,773	Input	no
Trompouki et al. 2011*	human	CEBPA-U937bio-r1-100709-5	0.58	26.28	0.3	-1	35	35	35	4,430,334	ChIP	yes
Trompouki et al. 2011*	human	CEBPA-U937dmso-r1-100505-5	0.41	23.29	0.18	-2	36	36	36	151,538	ChIP	yes
Trompouki et al. 2011*	human	CEBPA-K562-CEBPA-bmp4	0.46	10.99	0.34	-1	35	35	35	2,662,588	ChIP	yes
Trompouki et al. 2011*	human	CEBPA-U937-bmp4	0.64	3.16	0.44	-1	36	36	36	228,810	ChIP	yes
Trompouki et al. 2011*	human	GATA1-K562bio-r1-110325-6	0.49	11.55	0.09	-2	39	39	39	245,220	ChIP	yes
Trompouki et al. 2011*	human	GATA1-K562bmp-r1-110325-4	0.67	11.66	0.16	-2	36	36	36	335,062	ChIP	yes
Trompouki et al. 2011*	human	GATA1-K562	0.57	3.41	0.11	-2	36	36	36	371,785	ChIP	yes
Trompouki et al. 2011*	human	GATA2-K562bio-r1-110325-5	0.51	5.76	0.13	-2	39	39	39	190,367	ChIP	yes
Trompouki et al. 2011*	human	GATA2-K562bmp-r1-110325-3	0.27	5.97	0.14	-2	36	36	36	405,703	ChIP	yes
Trompouki et al. 2011*	human	GATA2-K562	0.47	10.43	0.02	-2	36	36	36	451,795	ChIP	yes
Trompouki et al. 2011*	human	Input-K562-CEBPA-bmp4	0.08	27.04	0.2	-2	39	39	39	248,035	Input	no
Trompouki et al. 2011*	human	SMAD1-K562bmp4-r1-100608-2	0.75	8	0.09	-2	35	35	35	834,331	ChIP	yes
Trompouki et al. 2011*	human	SMAD1-K562campk-r1-110323-2	0.71	2.48	0.14	-2	36	36	36	645,936	ChIP	yes
Trompouki et al. 2011*	human	SMAD1-U937bmp4-r1-100608-1	0.69	12.93	0.24	-2	36	36	36	1,184,890	ChIP	yes
Trompouki et al. 2011*	human	SMAD1-K562-CEBPA-bmp4	0.83	16.08	0.21	-2	36	36	36	3,126,161	ChIP	yes
Trompouki et al. 2011*	human	TCF7L2-K562bio-r1-100106-7	0.75	10.1	0.05	-2	36	36	36	88,763	ChIP	yes
Trompouki et al. 2011*	human	TCF7L2-K562bio-r2-Childrens	0.73	5.78	0.07	-2	40	40	40	116,187	ChIP	yes
Trompouki et al. 2011*	human	TCF7L2-U937bio-r1-100505-7	0.39	11.03	0.18	-2	36	36	36	145,311	ChIP	yes
Trompouki et al. 2011*	human	WCE-K562bio-r1-100106-5	0.72	8.07	0.02	-2	36	36	36	163,001	Input	no
Trompouki et al. 2011*	human	WCE-K562bio-r1-100608-2	0.67	5.23	0.02	-2	36	36	36	314,395	Input	no
Trompouki et al. 2011*	human	WCE-K562bmp4-r1-100608-1	0.67	8.04	0.05	-2	36	36	36	340,757	Input	no
Trompouki et al. 2011*	human	WCE-U937bio-r1-100505-6	0.61	4.26	0.07	-2	36	36	36	294,327	Input	no
Trompouki et al. 2011*	human	WCE-U937bio-r1-100608-5	0.64	3.52	0.06	-2	36	36	36	326,001	Input	no
Trompouki et al. 2011*	human	WCE-U937bio-r1-100709-4	0.62	3.02	0.06	-2	36	36	36	308,988	Input	no
Trompouki et al. 2011*	human	WCE-U937bio-r1-100709-6	0.62	2.64	0.06	-2	36	36	36	299,190	Input	no
Trompouki et al. 2011*	human	WCE-U937bmp4-r1-100608-3	0.65	3.73	0.04	-2	36	36	36	314,568	Input	no
Trompouki et al. 2011*	human	WCE-U937dmso-r1-100505-3	0.63	4.64	0.07	-2	36	36	36	272,327	Input	no
Ceschin et al. 2011	human	H3396-CARM1-E2	0.88	2.05	0.19	-2	49	49	49	99,711	ChIP	yes
Ceschin et al. 2011	human	H3396-CARM1-EtOH	0.88	2.15	0.2	-2	49	49	49	105,031	ChIP	yes
Ceschin et al. 2011	human	H3396-CBP-E2	0.51	4.35	0.02	-2	36	36	36	152,170	ChIP	yes
Ceschin et al. 2011	human	H3396-CBP-EtOH-1	0.33	8.51	0.06	-2	36	36	36	289,897	ChIP	yes
Ceschin et al. 2011	human	H3396-CBPR2151m-E2	0.44	7.62	0.03	-2	36	36	36	168,286	ChIP	yes
Ceschin et al. 2011	human	H3396-CBPR2151m-EtOH	0.46	8.58	0.03	-2	36	36	36	149,173	ChIP	yes
Ceschin et al. 2011	human	H3396-CBPR742m-E2-1	0.39	1.93	0.07	-2	36	36	36	383,127	ChIP	yes
Ceschin et al. 2011	human	H3396-CBPR768m-E2	0.41	10.17	0.04	-2	40	40	40	168,599	ChIP	yes
Ceschin et al. 2011	human	H3396-CBPR768m-EtOH	0.4	3.59	0.12	-2	40	40	40	157,261	ChIP	yes
Ceschin et al. 2011	human	H3396-ERa-E2	0.47	8.8	0.01	-2	36	36	36	105,766	ChIP	yes
Ceschin et al. 2011	human	H3396-ERa-EtOH	0.51	10.95	0.02	-2	36	36	36	73,181	ChIP	no
Ceschin et al. 2011	human	H3396-Input-E2-rep1-1	0.71	9.46	0.03	-2	36	36	36	92,932	Input	no
Ceschin et al. 2011	human	H3396-RAC3-E2	0.52	6.03	0.02	-2	36	36	36	110,731	ChIP	yes

Continued on next page

Source	Species	Library	Complexity	NSC	RSC	QC	Ave. Read Length	Max. Read Length	Min. Read Length	Mapped reads	Type	Should exhibit read clustering
Mendoza-Parra et al. 2011	mouse	mouse-F9-WCE	0.87	8.28	2.79	2	36	36	36	6,377,439	Input	no
Mendoza-Parra et al. 2011	mouse	RARg-24h-ATRA	0.87	4.27	1.95	2	36	36	36	5,864,836	ChIP	yes
Mendoza-Parra et al. 2011	mouse	RARg-2h-ATRA	0.87	4.27	1.92	2	36	36	36	6,545,542	ChIP	yes
Mendoza-Parra et al. 2011	mouse	RARg-48h-ATRA	0.91	3.46	1.82	2	36	36	36	3,543,638	ChIP	yes
Mendoza-Parra et al. 2011	mouse	RARg-48h-EtOH	0.8	4.55	0.93	0	36	36	36	6,281,297	ChIP	unknown
Mendoza-Parra et al. 2011	mouse	RARg-6h-ATRA	0.65	5.31	1.93	2	36	36	36	6,353,453	ChIP	yes
Mendoza-Parra et al. 2011	mouse	RXRa-24h-ATRA	0.67	4.42	1.29	1	36	36	36	6,444,150	ChIP	yes
Mendoza-Parra et al. 2011	mouse	RXRa-2h-ATRA	0.56	9.77	3.79	2	36	36	36	6,676,769	ChIP	yes
Mendoza-Parra et al. 2011	mouse	RXRa-48h-ATRA	0.6	11.1	3.89	2	36	36	36	5,869,783	ChIP	yes
Mendoza-Parra et al. 2011	mouse	RXRa-48h-EtOH	0.7	5.14	1.32	1	36	36	36	6,631,973	ChIP	unknown
Mendoza-Parra et al. 2011	mouse	RXRa-6h-ATRA	0.54	7.61	3.08	2	36	36	36	5,834,436	ChIP	yes
Mendoza-Parra et al. 2011	mouse	rxra-ko-RXRa-48h-ATRA	0.89	2.86	0.88	0	36	36	36	4,573,205	ChIP	yes
Schmitz et al. 2011	mouse	mESC-Jarid1b-1	0.87	1.25	0.34	-1	34	34	34	3,996,359	ChIP	yes
Schmitz et al. 2011	mouse	mESC-Jarid1b-2	0.88	1.24	0.36	-1	26	26	26	3,488,817	ChIP	yes
Bergsland et al. 2011	mouse	C2C12-Sox3-transfected-Sox3	0.77	1.44	0.85	0	53	53	53	29,894,751	ChIP	yes
Bergsland et al. 2011	mouse	Early-formed-neurons-IgG	0.93	2.05	0.2	-2	33	33	33	2,107,025	IgG	no
Bergsland et al. 2011	mouse	Early-formed-neurons-Sox11-rep1	0.94	2.15	0.27	-1	33	33	33	2,103,532	ChIP	yes
Bergsland et al. 2011	mouse	Early-formed-neurons-Sox11-rep2	0.95	1.99	0.27	-1	33	33	33	2,328,712	ChIP	yes
Bergsland et al. 2011	mouse	Early-formed-neurons-Sox11-rep3	0.96	1.67	0.34	-1	33	33	33	2,668,012	ChIP	yes
Bergsland et al. 2011	mouse	NPC-Sox2-rep1	0.9	1.38	0.29	-1	38	38	38	6,840,926	ChIP	yes
Bergsland et al. 2011	mouse	NPC-Sox2-rep2	0.74	1.62	0.69	0	38	38	38	12,391,326	ChIP	yes
Bergsland et al. 2011	mouse	NPC-Sox2-rep3	0.79	1.9	1.49	1	38	38	38	15,894,900	ChIP	yes
Bergsland et al. 2011	mouse	NPC-Sox3-rep1	0.88	2.68	1.34	1	34	34	34	3,339,224	ChIP	yes
Bergsland et al. 2011	mouse	NPC-Sox3-rep2	0.93	2.47	0.5	0	34	34	34	1,464,673	ChIP	yes
Bergsland et al. 2011	mouse	NPC-Sox3-rep3	0.87	2.87	2.24	2	34	34	34	3,496,087	ChIP	yes
Marban et al. 2011	human	Jurkat-Input	0.96	3.11	0.77	0	76	76	76	15,973,065	Input	no
Marban et al. 2011	human	Jurkat-Tat	0.94	4.07	1.04	1	76	76	76	18,900,158	ChIP	yes
Quenneville et al. 2011	mouse	mESC-HA	0.67	7.09	10.49	2	37.45	38	37	47,077,818	IgG	no
Quenneville et al. 2011	mouse	mESC-HAZFP57-HA	0.76	4.65	6.75	2	37.35	38	37	40,511,425	ChIP	yes
Quenneville et al. 2011	mouse	mESC-KAP1	0.63	4.31	7.86	2	49.63	76	38	58,793,249	ChIP	yes
Mullican et al. 2011	mouse	Macrophage-BSA-HDAC3	0.84	1.7	1.31	1	38	38	38	18,260,410	ChIP	yes
Mullican et al. 2011	mouse	Macrophage-IL4-HDAC3	0.89	1.64	1.18	1	38	38	38	17,042,856	ChIP	yes
Mullican et al. 2011	mouse	Macrophage-Input	0.95	1.09	0.36	-1	36	36	36	19,136,736	Input	no
Brown et al. 2011	human	hESC-D0-Smad-XL-rep1	0.95	1.67	0.42	-1	38	38	38	5,323,799	ChIP	yes
Brown et al. 2011	human	hESC-D0-Smad-XL-rep2	0.72	1.44	0.51	0	36	36	36	30,063,231	ChIP	yes
Brown et al. 2011	human	hESC-D3-Smad-XL-rep1	0.97	1.62	0.36	-1	38	38	38	6,844,734	ChIP	yes
Brown et al. 2011	human	hESC-D3-Smad-XL-rep2	0.75	1.44	0.42	-1	36	36	36	29,936,111	ChIP	yes
Brown et al. 2011	human	hESC-Input-XL	0.98	1.37	0.44	-1	36	36	36	7,422,963	Input	no
Mazzoni et al. 2011	mouse	Progenitor-Motor-Neurons-Day4-iOlig2-V5	0.92	3.13	1.85	2	36	36	36	3,330,651	ChIP	yes
Mazzoni et al. 2011	mouse	Progenitor-Motor-Neurons-Day4-Olig2	0.9	5	1.52	2	36	36	36	8,348,180	ChIP	yes
Mazzoni et al. 2011	mouse	Progenitor-Motor-Neurons-Day4-V5-control	0.93	1.48	0.48	-1	36	36	36	13,581,601	Input	no
Mazzoni et al. 2011	mouse	Progenitor-Motor-Neurons-Day5-iFlag-Hoxc9	0.87	3.68	2.59	2	36	36	36	29,775,081	ChIP	yes
Mazzoni et al. 2011	mouse	Progenitor-Motor-Neurons-Day5-iHoxc9-V5	0.71	2.48	2.42	2	69.05	76	36	28,150,488	ChIP	yes
Tan et al. 2011	human	LNCap-DHT-AR-1	0.83	11.17	1.68	2	36	36	36	13,158,813	ChIP	yes
Tan et al. 2011	human	LNCap-DHT-FoxA1-1	0.89	9.94	2.58	2	36	36	36	18,910,797	ChIP	yes
Tan et al. 2011	human	LNCap-DHT-NKX3-1	0.93	1.98	0.62	0	36	36	36	11,840,488	ChIP	yes
Tan et al. 2011	human	LNCap-EtOH-AR-1	0.92	2.71	0.92	0	36	36	36	10,786,161	ChIP	unknown
Tan et al. 2011	human	LNCap-EtOH-FoxA1	0.96	9.35	2.52	2	36	36	36	5,367,267	ChIP	yes
Tan et al. 2011	human	LNCap-EtOH-NKX3-1	0.91	1.59	0.51	0	36	36	36	16,850,974	ChIP	yes
Tan et al. 2011	human	LNCaP-Genomic-Input-1	0.95	1.54	0.51	0	36	36	36	10,550,285	Input	no
Shen et al. 2011	mouse	Heart-input1	0.87	1.86	0.47	-1	36	36	36	5,928,909	Input	no
Shen et al. 2011	mouse	Heart-input2	0.95	1.38	0.41	-1	36	36	36	6,264,090	Input	no
Shen et al. 2011	mouse	Heart-input3	0.94	1.21	0.48	-1	36	36	36	10,837,874	Input	no
Shen et al. 2011	mouse	Heart-Tbx20-GFP	0.95	1.9	0.63	0	36	36	36	23,754,878	ChIP	yes
Seitz et al. 2011	human	BL41-Input	0.98	1.19	0.1	-2	31	31	31	1,972,404	Input	no

Continued on next page

Source	Species	Library	Complexity	NSC	RSC	QC	Ave. Read Length	Max. Read Length	Min. Read Length	Mapped reads	Type	Should exhibit read clustering
Seitz et al. 2011	human	BL41-Myc	0.86	3.51	1.22	1	33	33	33	2,719,977	ChIP	yes
Seitz et al. 2011	human	Blue1-Input	0.99	1.27	0.12	-2	31	31	31	1,765,339	Input	no
Seitz et al. 2011	human	Blue1-Myc	0.98	2.46	0.66	0	31	31	31	1,884,244	ChIP	yes
Seitz et al. 2011	human	CA46-Input	0.98	1.2	0.13	-2	31	31	31	1,492,644	Input	no
Seitz et al. 2011	human	CA46-Myc	0.93	1.4	0.14	-2	71	71	71	1,734,564	ChIP	yes
Seitz et al. 2011	human	Raji-Input	0.95	1.26	0.16	-2	34	34	34	3,027,850	Input	no
Seitz et al. 2011	human	Raji-Myc	0.82	1.22	0.25	-2	34	34	34	2,186,015	ChIP	yes
Seitz et al. 2011	human	Ramos-Input	0.98	1.2	0.14	-2	31	31	31	1,880,856	Input	no
Seitz et al. 2011	human	Ramos-Myc	0.94	2.09	0.61	0	33	33	33	3,293,975	ChIP	yes
Little et al. 2011	human	C4-2B-Input	0.93	1.05	0.33	-1	50	50	50	85,985,363	Input	no
Little et al. 2011	human	C4-2B-Runx2	0.18	2.9	3.54	2	50	50	50	63,645,646	ChIP	yes
Whyte et al. 2011	mouse	mES-CoREST	0.94	1.37	0.39	-1	36	36	36	9,515,699	ChIP	yes
Whyte et al. 2011	mouse	mES-HDAC1	0.33	4.48	2.65	2	36	36	36	17,775,205	ChIP	yes
Whyte et al. 2011	mouse	mES-HDAC1-rep2	0.13	2.33	1.22	1	36	36	36	27,399,530	ChIP	yes
Whyte et al. 2011	mouse	mES-HDAC2	0.69	2.49	1.65	2	36	36	36	14,740,848	ChIP	yes
Whyte et al. 2011	mouse	mES-HDAC2-rep2	0.16	2.29	1.74	2	36	36	36	25,056,680	ChIP	yes
Whyte et al. 2011	mouse	mES-LSD1	0.94	1.54	0.79	0	36	36	36	3,907,159	ChIP	yes
Whyte et al. 2011	mouse	mES-LSD1-rep2	0.93	2.25	1.23	1	36	36	36	24,506,916	ChIP	yes
Whyte et al. 2011	mouse	mES-Mi-2	0.42	1.54	0.56	0	36	36	36	24,712,531	ChIP	yes
Whyte et al. 2011	mouse	mES-Mi-2b	0.95	1.27	0.45	-1	36	36	36	10,665,386	ChIP	yes
Whyte et al. 2011	mouse	mES-REST	0.73	3.31	1.57	2	36	36	36	24,569,235	ChIP	yes
Whyte et al. 2011	mouse	WCE-DMSO-t0	0.71	2.01	0.9	0	36	36	36	11,409,350	Input	no
Whyte et al. 2011	mouse	WCE-DMSO-t48	0.77	2.31	1.4	1	36	36	36	13,324,722	Input	no
Whyte et al. 2011	mouse	WCE-TCP-48	0.76	2.37	1.41	1	36	36	36	12,021,728	Input	no
GSE25426	human	THP-1-Control	0.94	1.29	0.36	-1	36	36	36	21,074,660	Input	no
GSE25426	human	THP-1-PPARg	0.96	1.82	0.63	0	36	36	36	14,473,006	ChIP	yes
GSE25426	human	THP-1-PU.1	0.95	4.78	2.12	2	35	35	35	13,571,315	ChIP	yes
GSE25426	human	THP-1-RXR	0.98	1.46	0.29	-1	35	35	35	6,999,922	ChIP	yes
Yildirim et al. 2011	mouse	mESC-Brg1-KD-Mbd3	0.96	2.73	0.15	-2	36	36	36	1,656,511	ChIP	unknown
Yildirim et al. 2011	mouse	mESC-Mbd3-rep1	0.94	2.21	0.33	-1	36	36	36	2,189,692	ChIP	yes
Yildirim et al. 2011	mouse	mESC-Mbd3-rep2	0.85	1.23	0.61	0	36	36	36	15,055,944	ChIP	yes
Yildirim et al. 2011	mouse	mESC-Tet1-KD-Mbd3	0.97	1.56	0.25	-1	36	36	36	3,626,622	ChIP	unknown
Botcheva et al. 2011	human	IMR90-Input	0.87	1.27	0.21	-2	36	36	36	9,286,134	Input	no
Botcheva et al. 2011	human	IMR90-p53	0.7	2.66	0.61	0	36	36	36	5,285,892	ChIP	yes
Stadler et al. 2011	mouse	ES-CTCF-rep1	0.64	28.28	1.67	2	37	37	37	10,466,451	ChIP	yes
Stadler et al. 2011	mouse	ES-CTCF-rep2	0.44	26.16	2.87	2	38	38	38	13,296,384	ChIP	yes
Stadler et al. 2011	mouse	ES-CTCF-rep3	0.49	9.15	8.28	2	38	38	38	9,587,128	ChIP	yes
Stadler et al. 2011	mouse	ES-Input-rep1	0.82	1.77	1.05	1	38	38	38	11,095,374	Input	no
Stadler et al. 2011	mouse	ES-Input-rep2	0.83	1.94	2.94	2	38	38	38	29,650,665	Input	no
Stadler et al. 2011	mouse	TKO-CTCF-rep1	0.63	10.83	3.72	2	36	36	36	34,828,958	ChIP	yes
Stadler et al. 2011	mouse	TKO-CTCF-rep2	0.91	5.48	3.61	2	36	36	36	2,836,169	ChIP	yes
Holmstrom et al. 2011	mouse	Pancreas-Input	0.97	1.46	0.88	0	36	36	36	11,479,285	Input	no
Holmstrom et al. 2011	mouse	Pancreas-Lrh1	0.84	4.4	1.89	2	36	36	36	13,587,564	ChIP	yes
Xu et al. 2011	zebrafish	Mxtx2-4.5hpf	0.77	1.7	1.39	1	36	36	36	11,341,093	ChIP	yes
Xu et al. 2011	zebrafish	Nanog-like-3.5hpf	0.63	1.89	1.44	1	36	36	36	7,535,238	ChIP	yes
Xu et al. 2011	zebrafish	Nanog-like-4.5hpf	0.84	3.94	1.22	1	36	36	36	10,103,194	ChIP	yes
Xu et al. 2011	zebrafish	WCE-Mxtx2-4.5hpf	0.97	1.24	0.59	0	36	36	36	18,309,687	Input	no
Xu et al. 2011	zebrafish	WCE-Nanog-like-3.5hpf	0.91	1.63	0.9	0	36	36	36	11,252,453	Input	no
Xu et al. 2011	zebrafish	WCE-Nanog-like-4.5hpf	0.98	1.36	0.67	0	36	36	36	15,831,173	Input	no
Tiwari et al. 2011a; Tiwari et al. 2011b	mouse	ES-JNK13-biological-replicate-a	0.82	4.27	2.79	2	38	38	38	8,462,462	ChIP	yes
Tiwari et al. 2011a; Tiwari et al. 2011b	mouse	ES-JNK13-biological-replicate-b	0.51	10.19	3.89	2	38	38	38	8,175,875	ChIP	yes
Tiwari et al. 2011a; Tiwari et al. 2011b	mouse	ES-NFYA-biological-replicate-a	0.79	2.98	5.24	2	38	38	38	19,929,924	ChIP	yes
Tiwari et al. 2011a; Tiwari et al. 2011b	mouse	ES-NFYA-biological-replicate-b	0.66	3.93	7.62	2	38	38	38	24,051,713	ChIP	yes
Tiwari et al. 2011a; Tiwari et al. 2011b	mouse	Input	0.82	1.77	1.05	1	38	38	38	11,095,374	Input	no
Tiwari et al. 2011a; Tiwari et al. 2011b	mouse	NP-JNK13-biological-replicate-a	0.69	8.11	5.07	2	38	38	38	8,802,240	ChIP	yes
Tiwari et al. 2011a; Tiwari et al. 2011b	mouse	NP-JNK13-biological-replicate-b	0.92	2.07	1.37	1	38	38	38	9,691,977	ChIP	yes
Tiwari et al. 2011a; Tiwari et al. 2011b	mouse	NP-NFYA-biological-replicate-a	0.85	2.18	3.96	2	38	38	38	23,674,653	ChIP	yes
Tiwari et al. 2011a; Tiwari et al. 2011b	mouse	NP-NFYA-biological-replicate-b	0.86	1.97	3.33	2	38	38	38	21,717,487	ChIP	yes
Tiwari et al. 2011a; Tiwari et al. 2011b	mouse	TN-DMSO-JNK1-3	0.17	11.6	12.92	2	36	36	36	38,425,945	ChIP	yes
Tiwari et al. 2011a; Tiwari et al. 2011b	mouse	TN-JNK1-3-biological-replicate-a	0.35	17.31	5.6	2	38	38	38	8,678,605	ChIP	yes

Continued on next page

Table S1 – *Continued from previous page*

Source	Species	Library	Complexity	NSC	RSC	QC	Ave. Read Length	Max. Read Length	Min. Read Length	Mapped reads	Type	Should exhibit read clustering
Tiwari et al. 2011a; Tiwari et al. 2011b	mouse	TN-JNK1-3-biological-replicate-b	0.29	20.52	7.67	2	38	38	38	6,897,900	ChIP	yes
Tiwari et al. 2011a; Tiwari et al. 2011b	mouse	TN-JNKi-JNK1-3	0.35	5.86	12.97	2	36	36	36	42,637,275	ChIP	yes
Tiwari et al. 2011a; Tiwari et al. 2011b	mouse	TN-NFYA-biological-replicate-a	0.68	3.06	5.64	2	38	38	38	27,386,709	ChIP	yes
Tiwari et al. 2011a; Tiwari et al. 2011b	mouse	TN-NFYA-biological-replicate-b	0.84	2.15	3.33	2	38	38	38	25,748,779	ChIP	yes
Zhang et al. 2011	mouse	F-Bcl6-rep1-G51	0.91	2.05	1.74	2	36	36	36	7,810,319	ChIP	yes
Zhang et al. 2011	mouse	F-Bcl6-rep2-G65-M1	0.9	6.17	0.58	0	36	36	36	6,073,003	ChIP	yes
Zhang et al. 2011	mouse	F-Bcl6-rep3-G65-M2	0.75	2.83	1.16	1	36	36	36	6,266,286	ChIP	yes
Zhang et al. 2011	mouse	F-Bcl6-rep4-G65-M3	0.93	3.23	0.42	-1	36	36	36	12,764,985	ChIP	yes
Zhang et al. 2011	mouse	FH-STAT5-rep1-G66-M1	0.83	6.19	3.78	2	36	36	36	6,691,463	ChIP	yes
Zhang et al. 2011	mouse	FH-STAT5-rep2-G66-M2	0.69	5.59	2.97	2	36	36	36	6,110,031	ChIP	yes
Zhang et al. 2011	mouse	FH-STAT5-rep3-G66-M3	0.65	9.48	4.33	2	36	36	36	13,444,170	ChIP	yes
Zhang et al. 2011	mouse	FL-STAT5-rep1-G52	0.19	2.48	1.94	2	36	36	36	5,389,553	ChIP	yes
Zhang et al. 2011	mouse	FL-STAT5-rep2-G70-M3	0.52	3.27	1.47	1	36	36	36	5,884,969	ChIP	yes
Zhang et al. 2011	mouse	FL-STAT5-rep3-G72-M1	0.63	2.55	0.92	0	36	36	36	2,627,103	ChIP	yes
Zhang et al. 2011	mouse	IgG-control	0.62	1.66	0.81	0	35	35	35	11,562,651	IgG	no
Zhang et al. 2011	mouse	M-Bcl6-rep1-G49	0.49	3.13	1.72	2	35	35	35	18,985,967	ChIP	yes
Zhang et al. 2011	mouse	M-Bcl6-rep2-G50	0.8	2.86	2.36	2	35	35	35	14,452,480	ChIP	yes
Zhang et al. 2011	mouse	M-Bcl6-rep3-G71-M2	0.76	2.33	0.74	0	35	35	35	14,149,114	ChIP	yes
Zhang et al. 2011	mouse	MH-STAT5-rep1-G36	0.62	4.29	2.69	2	35	35	35	15,997,841	ChIP	yes
Zhang et al. 2011	mouse	MH-STAT5-rep2-G41	0.76	3.64	2.47	2	35	35	35	12,841,332	ChIP	yes
Zhang et al. 2011	mouse	MH-STAT5-rep3-G42	0.64	3.39	2.67	2	36	36	36	9,528,779	ChIP	yes
Zhang et al. 2011	mouse	ML-STAT5-rep1-G35	0.71	2.18	2.17	2	36	36	36	16,024,096	ChIP	yes
Zhang et al. 2011	mouse	ML-STAT5-rep2-G40	0.84	1.69	1.42	1	36	36	36	5,688,929	ChIP	yes
Smith et al. 2011	mouse	mES-ELL	0.79	1.71	0.79	0	40	40	40	16,754,758	ChIP	yes
Smith et al. 2011	mouse	mES-Input	0.96	1.38	0.76	0	40	40	40	19,454,353	Input	no
Nakayamada et al. 2011	mouse	CD4+-Tbet	0.56	3.09	1.78	2	36	36	36	23,421,318	ChIP	yes
Lu et al. 2012	human	IgG-1-BCBL1	0.94	2.23	1.07	1	36	36	36	19,209,840	IgG	no
Lu et al. 2012	human	IgG-2-BCBL1	0.81	1.44	0.3	-1	36	36	36	12,604,075	IgG	no
Lu et al. 2012	human	LANA-1-BCBL1	0.46	1.6	0.53	0	36	36	36	19,777,228	ChIP	yes
Lu et al. 2012	human	LANA-2-BCBL1	0.86	1.44	0.27	-1	36	36	36	11,880,205	ChIP	yes
Meyer et al. 2012	human	LS180-bCat-125-1	0.43	13.11	1.87	2	35.43	36	35	39,712,224	ChIP	yes
Meyer et al. 2012	human	LS180-bCat-Veh-1	0.43	6.85	2.83	2	35.1	36	35	25,024,509	ChIP	yes
Meyer et al. 2012	human	LS180-CDX2-125-1	0.57	4.36	1.81	2	35.59	36	35	38,267,118	ChIP	yes
Meyer et al. 2012	human	LS180-CDX2-Veh-1	0.56	4.57	1.71	2	35.6	36	35	34,581,066	ChIP	yes
Meyer et al. 2012	human	LS180-CEBPb-125-1	0.81	8.48	1.8	2	35	35	35	24,978,947	ChIP	yes
Meyer et al. 2012	human	LS180-CEBPb-Veh-1	0.05	8.15	1.82	2	35.75	36	35	78,542,681	ChIP	yes
Meyer et al. 2012	human	LS180-Input-1	0.15	1.83	1.02	1	35.66	36	35	54,134,263	Input	no
Meyer et al. 2012	human	LS180-RXR-125-1	0.09	7.72	1.64	2	36	36	36	29,948,896	ChIP	yes
Meyer et al. 2012	human	LS180-RXR-Veh-1	0.1	7.3	1.79	2	36	36	36	26,448,441	ChIP	yes
Meyer et al. 2012	human	LS180-TCF4-125-2	0.28	10.15	1.78	2	45.01	50	35	49,453,419	ChIP	yes
Meyer et al. 2012	human	LS180-TCF4-Veh-2	0.36	10.26	1.9	2	42.43	50	35	20,780,670	ChIP	yes
Meyer et al. 2012	human	LS180-VDR-125-1	0.23	5.74	3.37	2	36	36	36	4,734,750	ChIP	yes
Meyer et al. 2012	human	LS180-VDR-Veh-1	0.18	11.61	5.58	2	35.79	36	35	72,061,937	ChIP	unknown
Ntziachristos et al. 2012	mouse	DP-mnase-input-replicate-1	0.92	2.74	1.13	1	34	34	34	15,457,880	Input	no
Ntziachristos et al. 2012	mouse	DP-mnase-input-replicate-2	0.9	6.08	3.06	2	34	34	34	12,676,911	Input	no
Ntziachristos et al. 2012	mouse	T-ALL-mnase-input-replicate-1	0.58	1.7	0.18	-2	34	34	34	9,970,383	Input	no
Ntziachristos et al. 2012	mouse	T-ALL-mnase-input-replicate-2	0.86	2.17	0.68	0	34	34	34	12,351,316	Input	no
Ntziachristos et al. 2012	mouse	T-ALL-Notch1	0.75	2.23	1.97	2	34	34	34	15,248,670	ChIP	yes
Ntziachristos et al. 2012	mouse	T-ALL-sonicated-input	0.7	1.28	0.17	-2	34	34	34	12,479,110	Input	no
Cheng et al. 2012	human	Gdown1-Control	0.97	1.69	0.66	0	36	36	36	3,798,010	ChIP	yes
Cheng et al. 2012	human	Gdown1-Flavo	0.93	1.77	0.74	0	36	36	36	7,869,560	ChIP	yes
GSE33128	human	Gdown1-IMR90	0.67	2.83	1.46	1	36	36	36	13,781,340	ChIP	yes
GSE33128	human	IgG-IMR90	0.67	7.4	2.05	2	36	36	36	7,308,478	IgG	no
GSE33128	human	Input-IMR90	0.96	1.47	0.69	0	36	36	36	14,239,395	Input	no
GSE35109	human	ERa-ChIP-seq-1	0.86	1.46	1.99	2	51	51	51	48,891,564	ChIP	yes
GSE35109	human	ERa-ChIP-seq-2	0.7	2.02	3.17	2	51	51	51	52,808,583	ChIP	yes
GSE35109	human	ERa-ChIP-seq-3	0.3	5.75	5.72	2	51	51	51	46,155,863	ChIP	yes
GSE35109	human	ERa-ChIP-seq-4	0.8	1.64	2.76	2	51	51	51	57,965,746	ChIP	yes
Canella et al. 2012	mouse	INPUT-Rep1	0.8	1.37	1.93	2	75	75	75	31,537,710	Input	no
Canella et al. 2012	mouse	INPUT-Rep2	0.8	1.38	1.96	2	75	75	75	33,328,402	Input	no
Canella et al. 2012	mouse	RPB2-Rep1	0.8	1.55	1.74	2	75	75	75	35,847,372	ChIP	yes
Canella et al. 2012	mouse	RPB2-Rep2	0.83	1.94	1.75	2	75	75	75	30,551,646	ChIP	yes
Canella et al. 2012	mouse	RPC1-Rep1	0.9	1.9	1.48	1	75	75	75	23,033,105	ChIP	yes
Canella et al. 2012	mouse	RPC1-Rep2	0.91	1.72	1.31	1	75	75	75	22,145,329	ChIP	yes
Canella et al. 2012	mouse	RPC4-Rep1	0.87	1.9	1.49	1	75	75	75	25,973,018	ChIP	yes
Canella et al. 2012	mouse	RPC4-Rep2	0.86	1.83	1.6	2	75	75	75	31,517,301	ChIP	yes
Sadasivam et al. 2012	human	BMyb-HeLa-Rep1	0.88	1.36	0.32	-1	36	36	36	15,389,344	ChIP	yes

Continued on next page

Source	Species	Library	Complexity	NSC	RSC	QC	Ave. Read Length	Max. Read Length	Min. Read Length	Mapped reads	Type	Should exhibit read clustering
Sadasivam et al. 2012	human	BMyb-HeLa-Rep2	0.77	1.81	0.07	-2	36	36	36	1,052,761	ChIP	yes
Sadasivam et al. 2012	human	Input-HeLa-Rep1	0.71	1.79	0.69	0	36	36	36	17,569,472	Input	no
Sadasivam et al. 2012	human	Input-HeLa-Rep2	0.64	1.56	0.08	-2	36	36	36	1,586,928	Input	no
Sadasivam et al. 2012	human	LIN9-HeLa-Rep1	0.92	1.39	0.49	-1	36	36	36	17,000,309	ChIP	yes
Sadasivam et al. 2012	human	LIN9-HeLa-Rep2	0.88	1.41	0.05	-2	36	36	36	1,798,161	ChIP	yes
Boergesen et al. 2012	mouse	LXR-WT-Bexarotene	0.93	1.94	2.05	2	35	35	35	6,469,307	ChIP	yes
Boergesen et al. 2012	mouse	LXR-WT-Control	0.9	1.91	1.45	1	35	35	35	6,086,575	ChIP	unknown
Boergesen et al. 2012	mouse	LXR-WT-T0901317	0.93	2.21	3.81	2	35	35	35	6,773,502	ChIP	unknown
Boergesen et al. 2012	mouse	PPARalpha-LXRdKO-Control	0.69	4.14	4.68	2	35	35	35	12,603,632	ChIP	yes
Boergesen et al. 2012	mouse	PPARalpha-WT-Control	0.66	4.02	10.05	2	35	35	35	13,493,293	ChIP	yes
Boergesen et al. 2012	mouse	RXR-LXRdKO-Bexarotene	0.96	2.71	2	2	34	34	34	4,499,835	ChIP	yes
Boergesen et al. 2012	mouse	RXR-LXRdKO-Control	0.92	2.7	1.99	2	32	32	32	5,011,146	ChIP	yes
Boergesen et al. 2012	mouse	RXR-LXRdKO-T0901317	0.94	2.14	1.9	2	32	32	32	5,048,268	ChIP	unknown
Boergesen et al. 2012	mouse	RXR-WT-Bexarotene	0.94	2.57	1.82	2	34	34	34	4,819,549	ChIP	yes
Boergesen et al. 2012	mouse	RXR-WT-Control	0.95	1.96	1.33	1	32	32	32	5,847,078	ChIP	yes
Boergesen et al. 2012	mouse	RXR-WT-T0901317	0.93	3.08	2.13	2	32	32	32	5,510,973	ChIP	unknown
Schödel et al. 2012	human	HIF-1beta	0.37	3.41	1.44	1	51	51	51	7,729,167	ChIP	yes
Schödel et al. 2012	human	HIF-2alpha	0.64	3.11	1.21	1	51	51	51	1,885,345	ChIP	yes
Schödel et al. 2012	human	Pre-immune-control	0.29	4.94	1.87	2	51	51	51	5,806,061	IgG	no
Pehkonen et al. 2012	human	IgG-control	0.72	1.41	0.24	-2	36	36	36	15,281,888	IgG	no
Pehkonen et al. 2012	human	LXR-T09	0.87	1.47	0.29	-1	36	36	36	14,265,491	ChIP	yes
Pehkonen et al. 2012	human	LXR-vehicle	0.9	1.42	0.27	-1	36	36	36	14,289,777	ChIP	unknown
GSE30919	mouse	CapH2-Ab1-DMSO-NOT-NORMALIZED-mES-MM8	0.69	1.61	0.91	0	36	36	36	16,534,945	ChIP	yes
GSE30919	mouse	CapH2-Ab1-FLAVO-NOT-NORMALIZED-mES-MM8	0.71	1.61	0.83	0	36	36	36	15,830,789	ChIP	yes
GSE30919	mouse	CapH2-Ab1-WT-mES-MM8	0.66	1.73	0.92	0	36	36	36	16,607,056	ChIP	yes
GSE30919	mouse	CapH2-Ab2-WT-mES-MM8	0.9	1.41	0.78	0	36	36	36	17,717,075	ChIP	yes
GSE30919	mouse	Smc1-DMSO-NOT-NORMALIZED-mES-MM8	0.84	5.22	1.99	2	36	36	36	19,206,320	ChIP	yes
GSE30919	mouse	Smc1-FLAVO-NOT-NORMALIZED-mES-MM8	0.78	4.43	1.88	2	36	36	36	19,650,774	ChIP	yes
Gao et al. 2012	human	CBX2	0.83	1.51	0.52	0	46	46	46	11,796,622	ChIP	yes
Gao et al. 2012	human	FH-CBX2.HA	0.89	1.22	0.37	-1	36	36	36	20,303,587	ChIP	yes
Gao et al. 2012	human	FH-PCGF1.HA	0.82	1.35	0.35	-1	36	36	36	18,667,442	ChIP	yes
Gao et al. 2012	human	FH-PCGF2.HA	0.66	1.9	0.67	0	36	36	36	18,549,373	ChIP	yes
Gao et al. 2012	human	FH-PCGF4.HA	0.31	2.44	0.81	0	36	36	36	18,274,491	ChIP	yes
Gao et al. 2012	human	FH-PCGF5.HA	0.66	1.78	0.65	0	36	36	36	18,930,930	ChIP	yes
Gao et al. 2012	human	FH-PCGF6.HA	0.8	1.48	0.43	-1	36	36	36	19,548,786	ChIP	yes
Gao et al. 2012	human	FH-RING1B.HA	0.43	1.94	1.58	2	36	36	36	19,398,688	ChIP	yes
Gao et al. 2012	human	FH-RYBP.HA	0.83	1.32	0.36	-1	36	36	36	16,950,286	ChIP	yes
Gao et al. 2012	human	input	0.78	1.23	0.22	-2	36	36	36	19,426,459	Input	no
Gao et al. 2012	human	PCGF4	0.93	1.16	0.19	-2	46	46	46	14,654,954	ChIP	yes
Gao et al. 2012	human	RING1B	0.9	1.18	0.24	-2	46	46	46	19,431,342	ChIP	yes
Gao et al. 2012	human	RYBP	0.91	1.36	0.44	-1	46	46	46	15,442,467	ChIP	yes
Yu et al. 2012	mouse	CBFb-induced-1	0.62	1.62	2.88	2	39.24	40	36	58,627,013	ChIP	yes
Yu et al. 2012	mouse	CBFb-thymocyte-control	0.25	1.63	0.62	0	40	40	40	8,637,405	ChIP	yes
Yu et al. 2012	mouse	CBFb-thymocyte-Runx1KO	0.35	1.4	0.54	0	40	40	40	7,518,656	ChIP	yes
Yu et al. 2012	mouse	CBFb-uninduced-1	0.73	1.58	2.28	2	36	36	36	26,748,905	ChIP	yes
Yu et al. 2012	mouse	IgG-induced-1	0.13	4.73	3.85	2	39.39	40	36	60,744,963	IgG	no
Yu et al. 2012	mouse	IgG-thymocyte-control	0.11	7.03	0.93	0	40	40	40	10,387,710	IgG	no
Yu et al. 2012	mouse	IgG-thymocyte-Runx1KO	0.04	9.33	0.8	0	40	40	40	11,696,369	IgG	no
Yu et al. 2012	mouse	IgG-uninduced-1	0.14	3.57	1.51	2	36	36	36	30,535,688	IgG	no
Yu et al. 2012	mouse	Ring1b-alt-ab	0.11	8.57	1.74	2	40	40	40	17,104,492	ChIP	yes
Yu et al. 2012	mouse	Ring1b-induced-1	0.67	1.5	2.59	2	39.31	40	36	65,911,236	ChIP	yes
Yu et al. 2012	mouse	Ring1b-thymocyte-control	0.21	3.38	0.58	0	40	40	40	7,476,406	ChIP	yes
Yu et al. 2012	mouse	Ring1b-thymocyte-Runx1KO	0.1	8.31	1.1	1	40	40	40	11,067,615	ChIP	yes
Yu et al. 2012	mouse	Ring1b-uninduced-1	0.38	2.1	3.58	2	36	36	36	31,481,625	ChIP	yes
Yu et al. 2012	mouse	Runx1-for-Ring1b-alt-ab	0.2	6.16	2.13	2	40	40	40	14,979,699	ChIP	yes
Yu et al. 2012	mouse	Runx1-induced-1	0.45	1.72	3.1	2	39.26	40	36	65,873,746	ChIP	yes
Yu et al. 2012	mouse	Runx1-thymocyte-control	0.24	5.51	1.23	1	40	40	40	8,075,699	ChIP	yes
Yu et al. 2012	mouse	Runx1-thymocyte-Runx1KO	0.17	4.65	0.84	0	40	40	40	9,234,112	ChIP	no
Yu et al. 2012	mouse	Runx1-uninduced-1	0.65	1.55	1.73	2	36	36	36	23,915,032	ChIP	yes
GSE29180	human	Jurkat-GATA3	0.75	4.85	0.85	0	36	36	36	4,308,315	ChIP	yes
GSE29180	human	Jurkat-Input-Rep1	0.97	1.19	0.42	-1	36	36	36	12,308,677	Input	no
GSE29180	human	Jurkat-RUNX1-Rep1	0.61	2.83	0.82	0	36	36	36	5,791,954	ChIP	yes
GSE29180	human	Jurkat-RUNX1-Rep2	0.53	2.44	0.53	0	36	36	36	5,800,696	ChIP	yes
GSE29180	human	Jurkat-RUNX1-Rep3	0.44	4.33	0.89	0	36	36	36	3,795,121	ChIP	yes
GSE29180	human	Jurkat-TAL1-Rep1	0.92	1.62	0.38	-1	36	36	36	5,485,444	ChIP	yes
GSE29180	human	Jurkat-TAL1-Rep2	0.86	2.59	0.68	0	36	36	36	6,350,195	ChIP	yes
GSE29180	human	Jurkat-TCF12	0.81	1.53	0.38	-1	36	36	36	8,594,457	ChIP	yes

Continued on next page

Source	Species	Library	Complexity	NSC	RSC	QC	Ave. Read Length	Max. Read Length	Min. Read Length	Mapped reads	Type	Should exhibit read clustering
GSE29180	human	Jurkat-TCF3	0.84	1.51	0.24	-2	36	36	36	5,398,758	ChIP	yes
Sakabe et al. 2012	mouse	input-1	0.95	1.86	0.47	-1	36	36	36	6,264,090	Input	no
Sakabe et al. 2012	mouse	input-2	0.94	1.38	0.41	-1	36	36	36	10,837,874	Input	no
Sakabe et al. 2012	mouse	input-3	0.95	1.21	0.48	-1	36	36	36	23,754,878	Input	no
Sakabe et al. 2012	mouse	Tbx20-GFP	0.87	1.9	0.63	0	36	36	36	5,928,909	ChIP	yes
Miller et al. 2012	human	HCC-1428-LTED-ER	0.92	1.2	0.36	-1	43	43	43	23,589,680	ChIP	yes
Miller et al. 2012	human	MCF-7-LTED-ER	0.93	1.15	0.55	0	43	43	43	27,118,853	ChIP	yes
Hutchins et al. 2012	mouse	PEC-IL10-treated-Input	0.97	1.39	0.25	-1	49	49	49	4,244,316	Input	no
Hutchins et al. 2012	mouse	PEC-IL10-treated-STAT3	0.71	5.06	1.09	1	49	49	49	3,841,121	ChIP	yes
Hutchins et al. 2012	mouse	PEC-Untreated-Input	0.97	1.39	0.17	-2	49	49	49	4,321,159	Input	no
Hutchins et al. 2012	mouse	PEC-Untreated-STAT3	0.83	3.12	0.73	0	49	49	49	4,189,247	ChIP	unknown
Trowbridge et al. 2012	mouse	MLL1	0.96	1.44	0.35	-1	36	36	36	4,933,023	ChIP	yes
Xiao et al. 2012	mouse	E14-IgG	0.58	2.13	0.42	-1	100	100	100	3,823,799	IgG	no
Xiao et al. 2012	mouse	E14-TAF1	0.95	1.07	0.37	-1	75	75	75	22,675,646	ChIP	yes
Xiao et al. 2012	pig	piPSC-IgG	0.62	2.33	0.45	-1	75	75	75	3,237,532	IgG	no
Xiao et al. 2012	pig	piPSC-NANOG	0.79	1.06	0.2	-2	75	75	75	15,130,135	ChIP	yes
Xiao et al. 2012	pig	piPSC-OCT4	0.84	1.58	0.63	0	75	75	75	4,150,813	ChIP	yes
Xiao et al. 2012	pig	piPSC-p300	0.86	1.07	0.16	-2	75	75	75	27,328,401	ChIP	yes
Xiao et al. 2012	pig	piPSC-TAF1	0.76	1.06	0.23	-2	75	75	75	8,822,964	ChIP	yes
Doré et al. 2012; Chlon et al. 2012	mouse	G1ME-ETS1	0.91	1.37	1.33	1	36	36	36	31,187,821	ChIP	yes
Doré et al. 2012; Chlon et al. 2012	mouse	G1ME-GATA1	0.52	1.77	2.14	2	36	36	36	35,032,324	ChIP	yes
Doré et al. 2012; Chlon et al. 2012	mouse	G1ME-GATA2	0.96	1.8	1.22	1	36	36	36	10,496,766	ChIP	yes
Doré et al. 2012; Chlon et al. 2012	mouse	G1ME-INPUT-GAII	0.93	1.23	0.19	-2	36	36	36	10,209,628	Input	no
Doré et al. 2012; Chlon et al. 2012	mouse	G1ME-INPUT-GAIIx	0.63	1.42	1.59	2	36	36	36	20,517,340	Input	no
Li et al. 2012	mouse	Input-seq-Adr8h	0.86	1.98	1.75	2	35	35	35	22,456,496	Input	no
Li et al. 2012	mouse	Input-seq-untreated	0.68	3.73	4.81	2	35	35	35	24,631,682	Input	no
Li et al. 2012	mouse	p53-Adr8h	0.74	8.66	2.17	2	35	35	35	22,316,127	ChIP	yes
Li et al. 2012	mouse	p53-untreated	0.95	5.29	1.57	2	35	35	35	9,544,532	ChIP	unknown
Li et al. 2012	mouse	p53S18P-Adr8h	0.92	14.22	1.65	2	35	35	35	9,487,356	ChIP	yes
Li et al. 2012	mouse	p53S18P-untreated	0.91	2.65	1.59	2	35	35	35	15,417,989	ChIP	unknown
Bugge et al. 2012; Feng et al. 2012	mouse	Reverb-alpha-null-5pm	0.63	1.91	1.73	2	50	50	50	82,551,235	ChIP	yes
Bugge et al. 2012; Feng et al. 2012	mouse	Reverb-beta-5am	0.83	1.7	0.73	0	36	36	36	7,098,042	ChIP	unknown
Bugge et al. 2012; Feng et al. 2012	mouse	Reverb-beta-5pm	0.18	2.08	1.77	2	36	36	36	39,165,327	ChIP	unknown
Gowher et al. 2012	human	HA-flag-Vezf1-Rep1	0.91	1.22	0.5	0	36	36	36	41,807,364	ChIP	yes
Gowher et al. 2012	human	HA-flag-Vezf1-Rep2	0.94	1.22	0.38	-1	36	36	36	10,730,653	ChIP	yes
Gowher et al. 2012	human	Input-HELA-Rep1	0.91	1.34	0.96	0	36	36	36	39,886,595	Input	no
Gowher et al. 2012	human	Input-HELA-Rep2	0.96	1.32	0.58	0	36	36	36	10,704,869	Input	no
Gowher et al. 2012	mouse	Input-mm9ES-wt	0.96	1.31	0.86	0	36	36	36	14,112,421	Input	no
Gowher et al. 2012	mouse	Input-Vezf1-ko	0.96	1.35	0.83	0	36	36	36	13,596,489	Input	no
GSE33346	mouse	CapD3-Nocodazole-mES	0.66	1.78	0.96	0	36	36	36	23,506,234	ChIP	unknown
GSE33346	mouse	CapD3-WT-mES	0.77	2.75	1.33	1	36	36	36	20,944,575	ChIP	yes
GSE33346	mouse	CapG-Nocodazole-mES	0.72	1.45	0.63	0	36	36	36	22,267,698	ChIP	unknown
GSE33346	mouse	CapG-WT-mES	0.73	1.62	1.11	1	36	36	36	23,314,867	ChIP	yes
GSE33346	mouse	CapH2-Nocodazole-mES	0.42	2.65	1.94	2	36	36	36	19,469,725	ChIP	unknown
GSE33346	mouse	CapH2-shGFP-mES	0.81	1.57	1.18	1	36	36	36	22,027,077	ChIP	yes
GSE33346	mouse	CapH2-shNipbl-mES	0.49	2.23	1.31	1	36	36	36	21,121,437	ChIP	unknown
GSE33346	mouse	Rad21-rep1-WT-mES	0.93	12.57	1.37	1	36	36	36	14,695,398	ChIP	yes
GSE33346	mouse	Rad21-rep2-WT-mES	0.85	13.29	2.19	2	36	36	36	20,290,096	ChIP	yes
GSE33346	mouse	WCE-Nocodazole-mES	0.61	1.54	0.97	0	36	36	36	22,934,718	Input	no
GSE33346	mouse	WCE-shGFP-mES	0.9	1.32	0.93	0	36	36	36	20,882,926	Input	no
GSE33346	mouse	WCE-shNipbl-mES	0.81	1.47	0.68	0	36	36	36	8,493,397	Input	no
GSE33850	human	E2A-CCRF-CEM	0.2	3.3	0.46	-1	40	40	40	9,580,539	ChIP	yes
GSE33850	human	GATA3-CCRF-CEM	0.14	4.27	0.54	0	40	40	40	8,433,815	ChIP	yes
GSE33850	human	HEB-CCRF-CEM-Rep1	0.11	5.07	0.79	0	39	39	39	11,256,332	ChIP	yes
GSE33850	human	HEB-CCRF-CEM-Rep2	0.15	4.66	0.66	0	40	40	40	13,394,868	ChIP	yes
GSE33850	human	Input-WCE-CCRF-CEM-Rep1	0.21	3.28	0.24	-2	39	39	39	3,524,267	Input	no
GSE33850	human	Input-WCE-CCRF-CEM-Rep2	0.81	1.33	0.08	-2	40	40	40	4,060,683	Input	no
GSE33850	human	Input-WCE-Prima2-T-ALL-Rep1	0.08	2.97	1.63	2	39	39	39	11,209,256	Input	no
GSE33850	human	Input-WCE-Prima2-T-ALL-Rep2	0.22	1.72	0.53	0	40	40	40	11,519,126	Input	no
GSE33850	human	Input-WCE-Prima5-T-ALL-Rep1	0.21	2.32	0.69	0	39	39	39	9,218,972	Input	no
GSE33850	human	Input-WCE-Prima5-T-ALL-Rep2	0.46	1.54	0.46	-1	40	40	40	10,635,018	Input	no
GSE33850	human	LMO1-Jurkat-Rep1	0.27	1.67	0.12	-2	40	40	40	6,940,746	ChIP	yes
GSE33850	human	LMO1-Jurkat-Rep2	0.57	1.79	0.27	-1	36	36	36	5,951,620	ChIP	yes

Continued on next page

Source	Species	Library	Complexity	NSC	RSC	QC	Ave. Read Length	Max. Read Length	Min. Read Length	Mapped reads	Type	Should exhibit read clustering
GSE33850	human	LMO2-CCRF-CEM-Rep1	0.11	2.74	0.28	-1	39	39	39	10,129,558	ChIP	yes
GSE33850	human	LMO2-CCRF-CEM-Rep2	0.15	2.72	0.25	-1	40	40	40	6,136,649	ChIP	yes
GSE33850	human	RUNX1-CCRF-CEM-Rep1	0.03	82.73	4.18	2	39	39	39	8,181,063	ChIP	yes
GSE33850	human	RUNX1-CCRF-CEM-Rep2	0.38	2.56	0.46	-1	40	40	40	12,118,147	ChIP	yes
GSE33850	human	TAL1-CCRF-CEM-Rep1	0.14	5.99	1.07	1	39	39	39	8,072,878	ChIP	yes
GSE33850	human	TAL1-CCRF-CEM-Rep2	0.17	3.12	0.68	0	40	40	40	17,651,204	ChIP	yes
GSE33850	human	TAL1-Prima2-T-ALL-Rep1	0.08	10.81	1.54	2	40	40	40	4,774,060	ChIP	yes
GSE33850	human	TAL1-Prima2-T-ALL-Rep2	0.05	10.97	1.65	2	39	39	39	7,554,079	ChIP	yes
GSE33850	human	TAL1-Prima5-T-ALL-Rep1	0.07	6.54	1.44	1	40	40	40	6,603,228	ChIP	yes
GSE33850	human	TAL1-Prima5-T-ALL-Rep2	0.05	7.04	1.56	2	39	39	39	9,252,579	ChIP	yes
Avvakumov et al. 2012	human	HBO1	0.96	1.24	1	0	36	36	36	31,901,032	ChIP	yes
Avvakumov et al. 2012	human	input	0.98	1.19	0.55	0	36	36	36	31,414,277	Input	no
Hunkapiller et al. 2012	mouse	InputDNA-Pcl3-shRNA	0.64	4.68	1.18	1	30	30	30	14,811,561	Input	no
Hunkapiller et al. 2012	mouse	InputDNA-Pcl3-shRNA6	0.95	1.2	0.4	-1	36	36	36	15,249,656	Input	no
Hunkapiller et al. 2012	mouse	InputDNA-Pcl3-shRNA7	0.95	1.22	0.55	0	36	36	36	19,965,283	Input	no
Hunkapiller et al. 2012	mouse	InputDNA-scramble	0.58	5.36	1.45	1	30	30	30	11,650,029	Input	no
Hunkapiller et al. 2012	mouse	Pcl3-shRNA6	0.88	1.28	1.38	1	36	36	36	14,295,321	ChIP	no
Hunkapiller et al. 2012	mouse	Pcl3-shRNA7	0.86	1.41	0.8	0	36	36	36	10,534,049	ChIP	no
Hunkapiller et al. 2012	mouse	Suz12-Pcl3-shRNA	0.78	1.41	1.43	1	30	30	30	13,893,316	ChIP	unknown
Hunkapiller et al. 2012	mouse	Suz12-scramble	0.78	1.77	1.38	1	30	30	30	11,020,925	ChIP	yes
Remeseiro et al. 2012	mouse	Input	0.89	1.22	0.56	0	40	40	40	25,401,900	Input	no
Remeseiro et al. 2012	mouse	InputMEFs	0.93	1.14	0.44	-1	40	40	40	27,631,354	Input	no
Remeseiro et al. 2012	mouse	KO-SA1	0.86	1.35	0.64	0	40	40	40	20,865,198	ChIP	no
Remeseiro et al. 2012	mouse	KO-SA2	0.79	1.49	0.95	0	40	40	40	26,737,423	ChIP	unknown
Remeseiro et al. 2012	mouse	SMC1-KO.R1	0.78	4.13	1.66	2	40	40	40	9,276,356	ChIP	unknown
Remeseiro et al. 2012	mouse	SMC1-KO.R2	0.82	3.98	1.93	2	40	40	40	12,183,058	ChIP	unknown
Remeseiro et al. 2012	mouse	SMC1-WT	0.91	1.91	1.38	1	40	40	40	22,390,032	ChIP	yes
Remeseiro et al. 2012	mouse	SMC3-KO	0.88	2.54	2.01	2	40	40	40	27,111,387	ChIP	unknown
Remeseiro et al. 2012	mouse	SMC3-WT	0.9	1.48	1.03	1	40	40	40	25,310,295	ChIP	yes
Remeseiro et al. 2012	mouse	WT-SA1	0.78	4.43	2.46	2	40	40	40	26,143,843	ChIP	yes
Remeseiro et al. 2012	mouse	WT-SA2	0.65	2.12	1.59	2	40	40	40	25,387,005	ChIP	yes
GSE36561	mouse	Brd4-mES	0.94	1.47	1.14	1	36	36	36	18,715,973	ChIP	yes
GSE36561	mouse	Brg1-mES	0.92	1.62	0.42	-1	36	36	36	4,204,507	ChIP	yes
GSE36561	mouse	SA1-mES-Rep1	0.95	13.34	2.23	2	36	36	36	6,935,496	ChIP	yes
GSE36561	mouse	SA1-mES-Rep2	0.84	21.27	2.29	2	36	36	36	18,853,613	ChIP	yes
GSE36561	mouse	SA2-mES-Rep1	0.94	16.15	2.13	2	36	36	36	7,883,128	ChIP	yes
GSE36561	mouse	SA2-mES-Rep2	0.84	15.88	2.29	2	36	36	36	18,512,023	ChIP	yes
Vilagos et al. 2012	mouse	EBF1-8246.2	0.96	3.03	1.04	1	36	36	36	5,435,592	ChIP	yes
Vilagos et al. 2012	mouse	EBF1-8246.6	0.96	3.1	1.13	1	36	36	36	7,748,856	ChIP	yes
Vilagos et al. 2012	mouse	EBF1-mature-B-8271	0.94	1.89	0.41	-1	36	36	36	5,327,224	ChIP	yes
Vilagos et al. 2012	mouse	EBF1-mature-B-9842	0.51	2.34	1.05	1	36	36	36	16,361,104	ChIP	yes
Vilagos et al. 2012	mouse	Rag2.Pro-B.input-8091.5	0.72	2.43	0.08	-2	36	36	36	2,188,795	Input	no
Vilagos et al. 2012	mouse	Rag2.Pro-B.input-8091.6	0.73	2.22	0.08	-2	36	36	36	2,267,935	Input	no
Vilagos et al. 2012	mouse	Rag2.Pro-B.input-8112.1	0.97	1.39	0.17	-2	36	36	36	4,627,018	Input	no
Vilagos et al. 2012	mouse	Rag2.Pro-B.input-8112.6	0.96	1.28	0.19	-2	36	36	36	6,424,234	Input	no
Vilagos et al. 2012	mouse	Rag2.Pro-B.input-8123.2	0.51	2.4	0.08	-2	36	36	36	2,220,931	Input	no
Vilagos et al. 2012	mouse	Rag2.Pro-B.input-8149.8.301DTAAXX	0.81	3.37	0.19	-2	36	36	36	1,534,780	Input	no
Vilagos et al. 2012	mouse	Rag2.Pro-B.input-8149.8.30222AAXX	0.86	2.37	0.57	0	36	36	36	5,533,095	Input	no
Vilagos et al. 2012	mouse	WT.Mature-B.Input-8042.5.208KBAAXX	0.87	1.32	0.1	-2	34	34	34	4,297,854	Input	no
Vilagos et al. 2012	mouse	WT.Mature-B.Input-8042.7.207JYAAXX	0.89	1.57	0.12	-2	36	36	36	3,133,666	Input	no
Vilagos et al. 2012	mouse	WT.Mature-B.Input-8042.7.20CUYAAXX	0.88	1.66	0.13	-2	36	36	36	3,220,120	Input	no
Vilagos et al. 2012	mouse	WT.Mature-B.Input-8042.8.208KDAAXX	0.87	3.9	0.2	-2	36	36	36	1,082,634	Input	no
Vilagos et al. 2012	mouse	WT.Mature-B.Input-8087	0.97	1.6	0.18	-2	32	32	32	4,201,759	Input	no
Vilagos et al. 2012	mouse	WT.Mature-B.Input-8089	0.96	1.59	0.22	-2	36	36	36	4,101,017	Input	no
Vilagos et al. 2012	mouse	WT.Mature-B.Input-8094	0.96	1.53	0.16	-2	36	36	36	3,717,876	Input	no
Vilagos et al. 2012	mouse	WT.Mature-B.Input-8096	0.95	1.33	0.17	-2	36	36	36	5,480,836	Input	no
Cardamone et al. 2012	human	GPS2	0.89	1.77	1.2	1	76	76	76	8,251,524	ChIP	yes
Cardamone et al. 2012	human	NCOR-siCTL	0.67	3	2.11	2	36.01	44	36	6,572,892	ChIP	yes
Cardamone et al. 2012	human	NCOR-siGPS2	0.72	2.5	0.78	0	36.01	44	36	5,121,903	ChIP	unknown
Cardamone et al. 2012	human	TBL1	0.87	1.87	1.93	2	36	36	36	9,798,221	ChIP	yes
Fan et al. 2012	mouse	HoxB4-day-16	0.95	2.1	1.08	1	41	41	41	8,877,542	ChIP	yes
Fan et al. 2012	mouse	HoxB4-day-26	0.94	4.72	1.79	2	41	41	41	10,871,546	ChIP	yes
Fan et al. 2012	mouse	HoxB4-day-6	0.92	2.36	1.63	2	36	36	36	6,336,688	ChIP	yes
Fan et al. 2012	mouse	Input-day-16	0.97	1.41	0.72	0	41	41	41	12,098,959	Input	no
Fan et al. 2012	mouse	Input-day-26	0.97	1.45	0.79	0	41	41	41	11,607,750	Input	no

Continued on next page

Table S1 – *Continued from previous page*

Source	Species	Library	Complexity	NSC	RSC	QC	Ave. Read Length	Max. Read Length	Min. Read Length	Mapped reads	Type	Should exhibit read clustering
Fan et al. 2012	mouse	Input-day-6	0.97	1.22	0.3	-1	36	36	36	8,817,894	Input	no
Fong et al. 2012	mouse	MM-MyoD	0.84	8.25	1.83	2	39	39	39	21,182,386	ChIP	yes
Fong et al. 2012	mouse	MM-NeuroD2	0.92	5.14	1.67	2	39	39	39	13,996,908	ChIP	yes
Fong et al. 2012	mouse	P19-control	0.97	1.42	0.56	0	38	39	37	8,903,023	IgG	no
Fong et al. 2012	mouse	P19-MyoD	0.92	12.89	1.94	2	39	39	39	12,117,729	ChIP	yes
Fong et al. 2012	mouse	P19-NeuroD2	0.94	7.18	1.67	2	39	39	39	14,558,083	ChIP	yes
Ptasinska et al. 2012	human	Input	0.88	1.35	0.2	-2	40	40	40	5,280,044	Input	no
Ptasinska et al. 2012	human	RUNX1-Kasumi-1	0.97	1.37	0.83	0	43.34	80	36	17,904,797	ChIP	yes
Ptasinska et al. 2012	human	RUNX1-non-t-8-21	0.91	3.67	1.81	2	36	36	36	30,747,325	ChIP	yes
Ptasinska et al. 2012	human	RUNX1ETO-control	0.95	1.79	0.97	0	75.95	80	40	7,462,090	ChIP	yes
Ptasinska et al. 2012	human	RUNX1ETO-siMM	0.94	1.65	0.97	0	73.57	80	40	12,843,591	ChIP	yes
Ptasinska et al. 2012	human	RUNX1ETO-siRE	0.82	2.82	1.2	1	67.36	80	40	5,525,324	ChIP	no
Cho et al. 2012	mouse	liver-input	0.78	1.54	1.25	1	42	42	42	29,085,894	Input	no
Cho et al. 2012	mouse	REV-ERBalpha	0.89	2.05	1.69	2	42	42	42	32,677,790	ChIP	yes
Cho et al. 2012	mouse	REV-ERBbeta	0.65	2.15	2.84	2	42	42	42	28,812,418	ChIP	yes
Wu et al. 2012	mouse	input-RUNX1	0.97	1.26	0.58	0	34	34	34	11,771,941	Input	no
Wu et al. 2012	mouse	input-TCF7	0.96	1.2	0.82	0	36	36	36	22,172,123	Input	no
Wu et al. 2012	mouse	RUNX1-Rep1	0.71	3.8	2.2	2	34	34	34	9,285,076	ChIP	yes
Wu et al. 2012	mouse	RUNX1-Rep2	0.68	4.01	2.32	2	34	34	34	10,064,029	ChIP	yes
Wu et al. 2012	mouse	TCF7	0.83	1.85	1	1	36	36	36	13,877,190	ChIP	yes
Barish et al. 2012	mouse	Bcl6-KO-macrophage-NCoR	0.66	1.75	1.37	1	42	42	42	25,491,046	ChIP	yes
Barish et al. 2012	mouse	Bcl6-KO-macrophage-SMRT	0.81	1.51	1.14	1	42	42	42	25,610,348	ChIP	yes
Barish et al. 2012	mouse	WT-macrophage-NCoR	0.84	1.81	1.79	2	43	43	43	24,281,787	ChIP	yes
Barish et al. 2012	mouse	WT-macrophage-SMRT	0.62	2.05	2.21	2	43	43	43	27,456,911	ChIP	yes

* Note: datasets from Trompouki et al. 2011 were excluded from figures as the vast majority of them had a very low number of mapped reads.
