# Supplementary material for: Large-Scale Quality Analysis of Published ChIP-seq Data
Source: G3 (Bethesda). 2013 Dec 17;4(2):209–23. doi: 10.1534/g3.113.008680 (PMC3931556; doi:10.1534/g3.113.008680)
Supplement: Supporting Information [file supp_g3.113.008680_TableS2.pdf]

Table S2: Dataset QC evaluation and mapping statistics for MyoD and myogenin datasets

Source	Species	Library	Complexity	NSC	RSC	QC	Ave. Read Length	Min. Read Length	Max. Read Length	Mapped reads	Type	Should exhibit read clustering
Wold Lab	mouse	C2C12 60h MyoD	0.90	12.39	1.65	2	36	36	36	6,771,837	ChIP	yes
Wold Lab	mouse	C2C12 60h myogenin 1	0.88	9.21	1.93	2	36	36	36	10,385,089	ChIP	yes
Wold Lab	mouse	C2C12 60h myogenin 2	0.97	6.95	1.32	1	36	36	36	1,198,656	ChIP	yes
Wold Lab	mouse	C2C12 60h myogenin 3	0.93	1.20	0.40	-1	36	36	36	19,600,577	ChIP	yes
Wold Lab	mouse	C2C12 60h 1%FA Input 3	0.94	1.22	0.46	-1	36	36	36	17,856,564	ChIP	no
Wold Lab	mouse	C2C12 60h 1%FA+EGS Input 3	0.87	4.88	1.52	2	36	36	36	9,092,000	ChIP	no
